# Taxonomic revision and phylogenetic analysis of the flightless Mancallinae (Aves, Pan-Alcidae)

**DOI:** 10.3897/zookeys.91.709

**Published:** 2011-04-20

**Authors:** Neil Adam Smith

**Affiliations:** Department of Entomology, University of Arizona, Tucson, Arizona 85721-0036, USA

**Keywords:** Cenozoic, convergence, diversity, evolution, fossil, *Miomancalla*

## Abstract

Although flightless alcids from the Miocene and Pliocene of the eastern Pacific Ocean have been known for over 100 years, there is no detailed evaluation of diversity and systematic placement of these taxa. This is the first combined analysis of morphological and molecular data to include all extant alcids, the recently extinct Great Auk *Pinguinus impennis*, the mancalline auks, and a large outgroup sampling of 29 additional non-alcid charadriiforms. Based on the systematic placement of Mancallinae outside of crown clade Alcidae, the clade name Pan-Alcidae is proposed to include all known alcids. An extensive review of the Mancallinae fossil record resulted in taxonomic revision of the clade, and identification of three new species. In addition to positing the first hypothesis of inter-relationships between Mancallinae species, phylogenetic results support placement of Mancallinae as the sister taxon to all other Alcidae, indicating that flightlessness evolved at least twice in the alcid lineage. Convergent osteological characteristics of Mancallinae, the flightless Great Auk, and Spheniscidae are summarized, and implications of Mancallinae diversity, radiation, and extinction in the context of paleoclimatic changes are discussed.

## Introduction

Alcidae Leach 1820 is a clade of pelagic wing-propelled-diving Charadriiformes Huxley 1867 including 23 extant species with an exclusively northern hemisphere distribution (del Hoyo et al. 1996). The fossil record indicates that alcid diversity during the Late Miocene (11.6–5.3 mya) and Early Pliocene (5.3–3.6 mya) equaled or exceeded extant alcid diversity (Olson 1985; Olson and Rasmussen 2001; Dyke and Walker 2005; Smith et al. 2007), although systematic evaluation of fossils referred to Alcidae is needed to refine estimates of paleodiversity in the clade. Additionally, the systematic position of most extinct species referred to Alcidae have yet to be evaluated in a phylogenetic analysis.

Primarily owing to the penguin-like characteristics of the flightless Great Auk *Pinguinus impennis* (Linnaeus 1758), alcids were grouped systematically with penguins and other ‘waterbirds’ including loons, grebes, and ducks by many ornithologists in the 18th and 19th centuries (Linnaeus 1758; Vigors 1825; Brandt 1837; Swainson 1837; Coues 1868), and this misconception lingered well into the 20th century (Verheyen 1958). However, there is consensus among modern classifications with regard to the placement of Alcidae in a monophyletic Charadriiformes (Ridgway 1919; Storer 1960; American Ornithologists’ Union 1998). Analyses of morphological (Strauch 1978; Björklund 1994; Chu 1995; Livezey and Zusi 2006, 2007; Livezey 2009, 2010; Mayr 2011) and molecular data (Sibley and Ahlquist 1972; Sibley and Ahlquist 1990; Ericson et al. 2003; Paton et al. 2003l; Thomas et al. 2004; Cracraft et al. 2004; Paton and Baker 2006; Fain and Houde 2007) support the charadriiform affinities of Alcidae. Furthermore, phylogenetic analyses of molecular data with dense taxonomic sampling for Alcidae support the monophyly of an extant alcid clade (Thomas et al. 2004; Baker et al. 2007; Pereira and Baker 2008). Previous morphology based analyses of alcid relationships have been limited with respect to taxon sampling. The compatability analysis of Strauch (1978) included only three alcid species and the subsequent analysis of alcid relationships (Strauch1985) did not include any outgroup taxa. The parsimony based analysis of alcid relationships by Chandler (1990a) was limited to a hypothetical outgroup terminal. The recent morphology based analyses of (Livezey and Zusi (2006, 2007) (Livezey (2009, 2010) and Mayr (2011) included Alcidae as a single, taxon level terminal.

**Figure 1.**
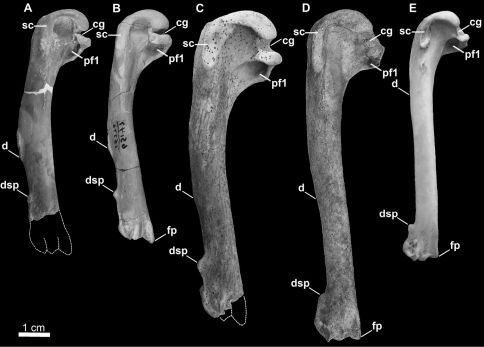
Comparison of alcid humeri in posterior view. Previously recognized Mancallinae holotype humeri along with examples of *Pinguinus impennis* and volant *Alca torda* humeri for comparison (dotted lines represent reconstructed parts of humeri). **A** Holotype specimen of *Mancalla californiensis* (USNM 4976) **B** Holotype humerus of *Mancalla cedrosensis* (LACM 15373) **C** Holotype specimen of *Miomancalla wetmorei* (LACM 42653) **D** *Pinguinus impennis* (USNM 623465) **E** *Alca torda* (NCSM 20058). Anatomical abbreviations: **cg** capital groove **d** deltopectoral crest **dsp** dorsal supracondylar process **fp** flexor process **pf1** primary pneumotricipital fossa **sc** supracoracoidal crest.

Although all extant alcids are volant, two lineages of extinct flightless auks are known. These flightless auks superficially resemble penguins, and share many morphological features convergent with those southern hemisphere wing-propelled divers such as an elongated first metacarpal and humeri with anteriorly rotated humeral heads (Miller and Howard 1949; see Appendix 1). During the Miocene and Pliocene a diverse assemblage of alcids including the flightless Great Auk *Pinguinus* Bonnaterre, 1790, and other volant auks such as *Alca* Linnaeus, 1758 and *Miocepphus* Wetmore, 1940 were present in the Atlantic Ocean (Olson and Rasmussen 2001; Wijnker and Olson 2009). Similarly, during the Miocene and Pliocene the Pacific was inhabited by a lineage of flightless alcids known as the Mancallinae Brodkorb 1967. Although Mancallinae(contents = *Mancalla* Lucas, 1901 + *Praemancalla* Howard, 1966; sensu Brodkorb 1967; Olson 1985) and *Pinguinus* share several morphological characteristics related to extreme adaptation for wing-propelled diving and the subsequent loss of aerial flight (Fig. 1), phylogenetic results indicate that these taxa are not closely related within Alcidae. *Pinguinus* is consistently recovered as the sister taxon to *Alca* (Chandler 1990a; Moum et. al. 2002; Baker et. al. 2007; Pereira and Baker 2008), and previousphylogenetic analyses place Mancallinae as the sister taxon to all other Alcidae (Chandler 1990a; Smith 2008), suggesting that flightlessness evolved separately in Mancallinaeand *Pinguinus*.

**Figure 2.**
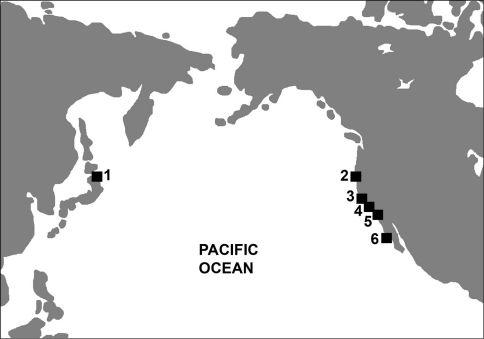
Map depicting Mancallinae fossil localities. **1** Shiriya, Honshu, Japan **2** Humboldt County, CA, USA **3** Los Angeles, CA, USA **4** Laguna Hills, and Laguna Niguel, CA, USA **5** San Diego, CA, USA **6** Cedros Island, Baja California, Mexico.

Fossil records of Mancallinae are restricted to the northern Pacific Ocean basin. Miocene and Pleistocene aged fossils have been reported from Japan (Hasegawa et al. 1988; Kohno 1997; Fig. 2), although these remains have not been systematically described or figured in publication. In contrast to the sparse record of the clade from the western Pacific Ocean, thousands of mostly isolated remains are known from California, USA and northern Baja California, Mexico (Miller and Howard 1949; Howard 1966, 1968, 1970, 1971, 1976, 1978, 1981, 1982; Chandler 1990b; Fig. 2), and range in age from Late Mioceneto Late Pleistocene (Table 1). The northernmost occurrence is in Humboldt County California (Howard 1970; Kohl 1974) and the southernmost occurrence is in Baja California, Mexico (Howard 1971).

**Table 1.** Mancallinaeholotype material. See Appendix 1 for details of the taxonomic revision.

Taxon	Holotypematerial	Provenience	Age	Reference	Taxonomic Status
*Mancalla californiensis*	Humerus	Los Angeles,CA	EarlyPliocene	Lucas 1901	*Mancalla californiensis*
*Mancalla diegensis*	Femur	San Diego,CA	EarlyPliocene	Miller 1937	Pan-Alcidae incertae sedis
*Praemancalla lagunensis*	Distal Humerus	Laguna Hills,CA	LateMiocene	Howard 1966	Mancallinae incertae sedis
*Alcodes ulnulus*	Ulna	Laguna Hills,CA	MiddleMiocene	Howard 1968	Pan-Alcidae incertae sedis
*Mancalla milleri*	Femur	San Diego,CA	EarlyPliocene	Howard 1970	Pan-Alcidae incertae sedis
*Mancalla cedrosensis*	PartialSkeleton	Baja Calif.,Mexico	LateMiocene	Howard 1971	*Mancalla cedrosensis*
*Praemancalla wetmorei*	Humerus	Laguna Niguel,CA	LateMiocene	Howard 1976	*Praemancalla wetmorei*
*Mancalla emlongi*	Ulna	San Diego,CA	EarlyPliocene	Olson 1981	Mancallinae incertae sedis
*Miomancalla howardi*	PartialSkeleton	San Diego,CA	LateMiocene	Smith 2011	*Miomancalla howardi*
*Mancall alucasi*	PartialSkeleton	San Diego,CA	EarlyPliocene	Smith 2011	*Mancall alucasi*
*Mancalla vegrandis*	PartialSkeleton	San Diego,CA	EarlyPliocene	Smith 2011	*Mancalla vegrandis*

Discovery of an articulated partial skeleton referable to Mancallinae (SDSNH 68312) from the Early Pliocene Capistrano Formation of Orange County California prompted a re-examination of diversity and morphological variation within this clade. Previously reported Mancallinae remains are reviewed (Appendix 1), and the results of an extensive survey of Mancallinae remains are reported. Three new species of Mancallinae are described, and the systematic placement of Mancallinae within Alcidae, as well as the inter-relationships of Mancallinae species is evaluated in a combined phylogenetic analyses of morphological and molecular sequence data. This study represents the first time that relationships among all 23 extant alcids and 29 other charadriiform outgroup taxa have been assessed in the context of a combined phylogenetic analysis.

## Materials and methods

### Anatomical terminology and taxonomic conventions

Description of anatomical features primarily follows the English equivalents of the Latin osteological nomenclature summarized by Baumel and Witmer (1993). The terminology of Howard (1929) is followed for features not treated by Baumel and Witmer (1993). Measurements follow those proposed by Von den Driesch (1976). All measurements were taken using digital calipers and rounded to the nearest tenth of a millimeter. Ages of geologic time intervals are based on the International Geologic Timescale (Gradstein et al. 2004; Ogg et al. 2008).

With the exception of species names (e.g., *Fratercula arctica*), which follow the 7th edition of the Checklist of North American Birds (American Ornithologists’ Union 1998) for extant species, all taxonomic designations (e.g., *Fratercula*) are intended as clade names as defined by the International Code of Phylogenetic Nomenclature (i.e., The PhyloCode v.4c; Cantino and de Queiroz 2010), regardless of use of italics or previous rank recognized by other authors, and are not intended to convey rank under the Linnaean system of nomenclature. The PhyloCode recommendation that all scientific names be italicized (Recommendation 6.1A) was not followed here. Only species names are italicized herein. Pursuant to Article 21.2 of the PhyloCode, the first word of species names are considered prenomen, not genus names (see also Dryat et al. 2008).

### Taxon and character sampling

All extinct taxa were evaluated by direct observation of holotype and referred specimens. Whenever available, a total of five or more specimens of each extant species (Appendix 2) including both sexes were evaluated to account for intraspecific character variation and sexual dimorphism respectively. Only adult specimens, assessed based upon degree of ossification (Chapman 1965), were evaluated for osteological characters, and when available, specimens from multiple locations within the geographic range of extant species (i.e., subspecies) were examined to account for geographic variation within species. Reproductive, chick integument, dietary, and some myological characters were scored from published sources (Appendix 3). Descriptions of anatomical characteristics are followed by character numbers and character state symbols from Appendix 3 (e.g., 23:0 = character number 23, character state 0).

The cladistic matrix (Appendix 4) includes 72 terminals, scored for a maximum of 344 morphological characters (284 binary; 60 multistate;15 ordered). All 23 extant alcids, the recently extinct Great Auk *Pinguinus impennis* Linnaeus, 1758, 18 Mancallinae specimens, and a Mancallinae supraspecific terminal are included in the matrix. Twenty-nine other extant charadriiforms comprise the remainder of the taxa analyzed, and provide a dense outgroup taxonomic sample to test the monophyly of extant and extinct alcids. with respect to other charadriiforms.Morphological characters include osteological (*n* = 223), integumentary (*n* = 32), ethological (*n* = 16), myological (*n* = 24) and micro-feather (*n* = 52). One hundred and fifty-five characters were newly identified for this analysis. The other 189 characters were drawn from the work of Hudson et al. (1969; *n* = 24), (Strauch (1978, 1985; *n* = 39), Chandler (1990a; *n* = 63), Chu (1998; *n* = 11), and Dove (2000; *n* = 34). Only 34 of the 38 characters used by Dove (2000) varied in the taxa examined in this study. Of the 34 used in this analysis, eighteen were modified (i.e., split into 2 separate characters) according to the philosophy of character independence proposed by Hawkins et al. (1997), resulting in a total of 52 microfeather characters.

The cladistic matrix also includes a molecular sequence alignment of 11,601 base pairs from eight DNA sequence types (including gaps). See Appendix 5 for details of sequence availability, inclusion for each species, and sequence authorship. Molecular sequence data (mitochondrial: ND2, ND5, ND6, CO1, CYTB; ribosomal RNA: 12S, 16S; and nuclear: RAG1) were downloaded from GenBank. Preliminary sequence alignments for each gene were obtained using the program ClustalX v2.0.6 (Thompson et al. 1997), and then manually adjusted using the program Se-Al v2.0A11 (Rambaut 2002).

### Phylogenetic analyses

A combined approach of phylogeny estimation was used to evaluate the systematic position of Mancallinae species. Simulations show that the combination of molecular and morphological data often provides a more accurate estimate of phylogeny with respect to both extant and extinct organisms (Wiens 2009). Phylogenetic analyses employed the parsimony criterion of phylogenetic inference as implemented in PAUP* v4.0b10 (Swofford 2002). Parsimony tree search criteria are as follows: heuristic search strategy; 10,000 random taxon addition sequences; tree bisection-reconnection branch swapping; random starting trees (primary analysis only); all characters equally weighted; minimum length branches = 0 collapsed; multistate (e.g., 0&1) scorings used only for polymorphism. Bootstrap values and descriptive tree statistics including consistency index (CI), retention index (RI), and rescaled consistency index (RC) were calculated using PAUP* v4.0b10 (Swofford 2002). Bootstrap value calculation parameters included 1,000 heuristic replicates, 100 random addition sequences per replicate. All other settings were the same as the primary analysis. Bremer support values were calculated using a script generated in MacClade v4.08 (Maddison and Maddison 2005) and analyzed with PAUP* v4.0b10 (Swofford 2002). Based on the results of previous phylogenetic analyses of charadriiform relationships (Strauch 1978; Sibley and Ahlquist 1990; Chu 1995; Ericson et al. 2003; Paton et al. 2003; Thomas et al. 2004;Baker et al. 2007) resultant trees were rooted with the clade represented by exemplars of *Charadrius vociferus* Linnaeus 1758 and *Charadrius wilsonia* Ord, 1814.

### Institutional abbreviations

AMNH—American Museum of Natural History, New York, NY, USA; GCVP—Georgia College and State University Vertebrate Paleontology Collection, Milledgeville, GA, USA; IVPP—Institute of Vertebrate Paleontology and Paleoanthropology, Beijing, China; LACM—Natural History Museum of Los Angeles County, Los Angeles, CA., USA; LM—Loye Miller Collection, location presently unknown; NSM PO—National Museum of Nature and Science Paleontology Osteological Collection, Tokyo, Japan; NCSM—North Carolina Museum of Natural Sciences, Raleigh, NC, USA; SDSNH—San Diego Natural History Museum, San Diego, CA, USA; TMM—Texas Natural Science Center Vertebrate Paleontology Laboratory, Austin, TX, USA; UCMP—University of California Museum of Paleontology, Berkeley, CA, USA; USNM—National Museum of Natural History, Smithsonian Institution, Washington, D.C., USA.

## Systematic Paleontology

**AVES Linnaeus, 1758**

**CHARADRIIFORMES Huxley, 1867**

**PAN-ALCIDAE new taxon.**

Pan-Alcidae (contents = Alcidae Leach, 1820 (i.e., the alcid crown clade) + Mancallinae) is differentiated from all other Charadriiformes by the following characteristics: quadrate apneumatic (38:1); reduced pneumatic foramen of anterior sternum (59:0); omal extremity of furcula angled sharply rather than gently curving as in other charadriiforms (75:1); coracoidal tuberosity of furcula, positioned anterior to coracoidal facet(77:1); dorsoventral compression of humeral shaft exceeds that of all other charadriiforms (141:1/2); bicipital tubercle of radius distally elongated rather than in the form of a rounded tubercle as in other charadriiforms (162:1). Apomorphies of the alcid crown clade, Alcidae, are provided in Table 4.

### Mancallinae Brodkorb, 1967

Mancallinae(contents = *Mancalla* + *Miomancalla* gen. n.) is referable to Pan-Alcidae based upon dorsoventral compression of the humeral shaft (141:2). The humeral shafts of Pan-Alcidae are more dorsoventrally compressed than in all other Charadriiformes. Mancallinae is differentiated from all other alcids on the basis of the following unambiguously optimized humeral apomorphies: deltopectoral crest extends past the midway point of the humeral shaft rather than restricted to the proximal half of the humeral shaft (104:2); presence of a ‘mancalline muscle scar’ extending distally from the primary pneumotricipital fossa (discussed below; 120:1); capital groove communicates with transverse ligament sulcus resulting a notched rather than rounded appearance of ventral margin of the humeral head in anterior view (136:2); humeral head rotated anterodorsally rather than in-line with humeral shaft (139:1); humeral shaft arced rather than sigmoidal (140:1); presence of fossae in tricipital sulci (150:1); anterior surface of the ventral condyle rounded rather than flattened (153:0). Additional proposed apomorphies of Mancallinae include distal elongation (184:1) and anterior flattening of the first metacarpal (185:1). These characteristics are present in *Mancalla cedrosensis* Howard, 1971, *Miomancalla howardi* sp. n.,and two additional associated specimens referable to Mancallinae (SDSNH 77966 and LACM 107028). Although these two characters are also diagnostic for Alcini Storer, 1960, the clade composed of *Alca*, *Pinguinus*, *Alle* Link, 1806, and *Uria* Brisson, 1760, the degree of distal elongation and anterior flattening in *Mancalla* exceeds that observed in Alcini.

#### Mancalla

Lucas, 1901

##### Original diagnosis

(sensu Lucas, 1901)—Referable to Alcidae based upon dorsoventral compression of the humeral shaft. Differs from other Alcidae in the following characteristics: humerus short, with arced rather than sigmoid lengthwise curvature; anterior rotation of the humeral head; ventral margin of m. brachialis scar a distinct ridge.

##### Amended diagnosis.

*Mancalla* is differentiated from *Miomancalla* on the basis of the following humeral characteristics: supracoracoidial crest does not broaden proximally (113:2); distal margin of the primary pneumotricipital fossa convex rather than concave (126:0); ventral margin of the ventral tubercle narrow and ventrally expanded (i.e., convex) rather than wide and deeply grooved (134:0); capital groove constricted rather than wide (137:1). Additional proposed apomorphies which are present in *Mancalla cedrosensis* and two additional associated specimens (SDSNH 77966 and LACM 128870) referable to *Mancalla* but not to species include: ulna shorter than carpometacarpus (180:1); ulna and radius more dorsoventrally compressed than other alcids; extension of the dorsal ulnar condyle farther distally to the ventral ulnar condyle than in other alcids (182:0); pisiform process of carpometacarpus reduced or absent (188:1).

#### Mancalla lucasi sp. n.

urn:lsid:zoobank.org:act:31389B4B-0A03-48E5-8A0B-C71CDBCE7164

##### Holotype.

**Figure 3.**
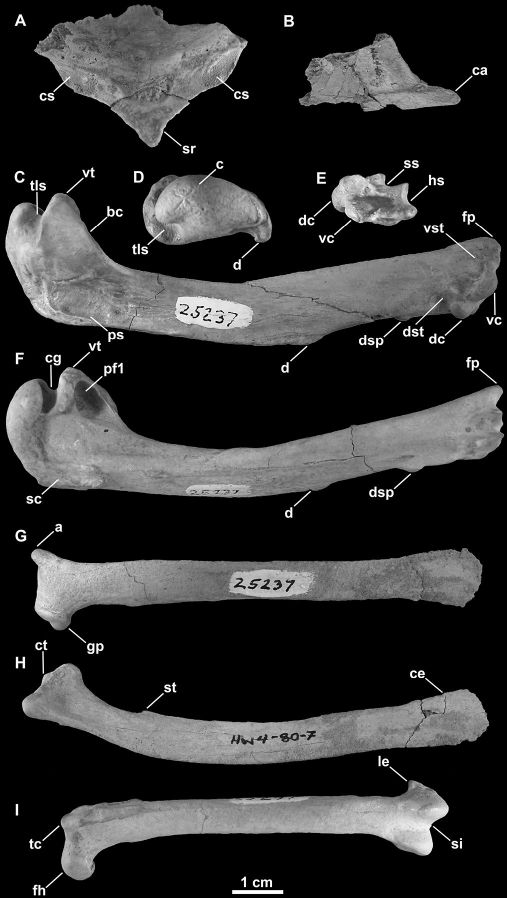
Holotype specimen of *Mancalla lucasi* (SDSNH 25237). **A** Fragment of anterior sternum in anterior view **B** Carinal apex of sternum in right lateral view **C** Right humerus in anterior view **D** Left humerus in proximal view **E** Left humerus in distal view **F** Left humerus in posterior view **G** Left scapula in lateral view **H** Right scapula in medial view **I** Left femur in anterior view. Anatomical abbreviations: **a** acromion process **bc** bicipital crest **c** caput **ca** carinal apex **ce** caudal extremity of scapula **cg** capital groove **cs** coracoidal sulcus **ct** coracoidal tubercle **d** deltopectoral crest **dc** dorsal condyle **dsp** dorsal supracondylar **dst** dorsal supracondylar tubercle **fh** femoral head **fp** flexor process **gp** glenoid process **hs** humerotricipital sulcus **le** lateral epicondyle **pf1** primary pneumotricipital fossa **ps** pectoralis scar **sc** supracoracoidal crest **si** sulcus intercondylaris **sr** sternal rostrum **ss** scapulotricipital sulcus **st** scapulotricipital tubercle **tc** trochanteric crest **tls** transverse ligament sulcus **vc** ventral condyle **vst** ventral supracondylar tubercle **vt** ventral tubercle.

SDSNH 25237: a partial postcranial skeleton comprising the following elements: right and left scapulae, partial sternum, right and left humeri, left femur (Fig. 3; Tables 1, 2 and 3). The holotype specimen was collected by H. M. Wagner in April, 1980.

**Table 2.** Measurements of Mancallinae holotype humeri (mm). Abbreviations: (Glh) greatest length of humerus; (Bph) breadth of proximal humerus; (Diph) diagonal of proximal humerus; (Whs) width of humeral shaft; (Bdh) breadth of distal humerus; (Ddh) depth of distal humerus. Measurements according to Von den Driesch (1976). ‘~’ signifies approximate measurement due to damage. ‘—‘ signifies missing data due to damage.

Species	Specimen #	Glh	Bph	Diph	Whs	Bdh	Ddh
*Mancalla californiensis*	USNM4976	~75.0	19.0	18.4	8.9	___	___
*Mancalla cedrosensis*	LACM15373	73.3	17.8	17.1	9.1	13.0	7.1
*Mancalla lucasi*	SDSNH25237	90.2	21.7	21.2	11.1	13.4	8.0
*Mancalla vegrandis*	SDSNH77399	61.8	15.1	14.3	7.4	9.5	5.8
*Miomancalla wetmorei*	LACM42653	~86.0	21.5	21.1	12.7	8.7	9.5
*Miomancalla howardi*	SDSNH24584	103.2	22.9	22.2	11.1	12.2	8.7
*Miomancalla howardi*	SDSNH68312	___	~25.0	~24.0	___	___	___

**Table 3.** Measurements of new associated Mancallinae holotype specimens (in mm). ‘-’ = missing data due to damage or lack of comparable element.

	*Miomancalla howardi*	*Mancalla lucasi*	*Mancalla vegrandis*
		SDSNH 68312	SDSNH 25237	SDSNH77399
SKULL & MANDIBLE			
Greatest length of skull	122.9	-	-
Greatest breadth of frontal	11.4	-	-
Greatest length of rostrum	84.2	-	-
Greatest height of rostrum	21.1	-	-
Greatest length of mandible	127.8	-	-
STERNUM			
Smallest width between costal processes	-	-	5.9
FURCULA			
Dorsoventral height of apophysis	-	-	2.8
CORACOID			
Greatest length	-	-	45.8
SCAPULA			
Greatest proximal height	-	15.1	10.9
CARPOMETACARPUS			
Greatest length	46.8	-	-
Length of metacarpal one	23.2	-	-
Proximal breadth	11.9	-	-
PELVIS			
Greatest length	127.8	-	74.8
FEMUR			
Greatest length	79.9	67.8	-
Medial length	78.0	64.9	-
Proximal breadth	17.8	12.9	-
Proximal depth	10.9	9.2	-
Breadth of shaft	8.3	7.5	-
Distal breadth	18.0	12.5	-
TIBIOTARSUS			
Greatest length (preserved)	113.7	-	-
Breadth of shaft	7.8	-	-

##### Etymology.

This new species is named in honor of Frederic A. Lucas who described the first known remains of *Mancalla*.

##### Locality and horizon.

Late Pliocene or Early Pleistocene (Zanclean or Calabrian) Niguel Formation of Orange County, California. Latitude, longitude, and elevation data are on file at SDSNH (locality 3202). Details of the geologic setting are provided in Appendix 6.

##### Referred specimen.

**Figure 4.**
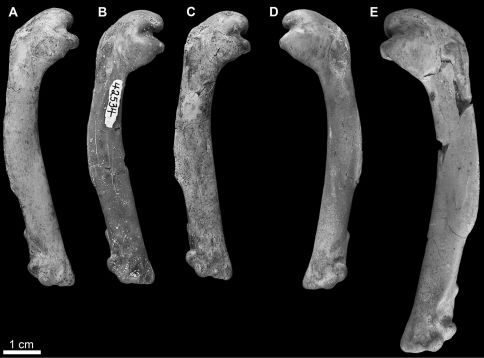
*Mancalla* referred humeri in anterior view. **A** *Mancalla vegrandis* SDSNH 28152 **B** *Mancalla vegrandis* SDSNH 42534 **C** *Mancalla vegrandis* SDSNH 75051 **D** *Mancalla vegrandis* SDSNH 42532 **E** *Mancalla lucasi* SDSNH 59049.

SDSNH 59049: a complete left humerus from the Middle Pliocene to Early Pleistocene San Diego Formation (SDSNH locality 3506; Fig. 4E).

##### Differential diagnosis.

Scar extending into primary pneumotricipital fossa is raised in relief to the floor of the primary pneumotricipital fossa and the humeral shaft as in *Mancalla cedrosensis*,rather than an excavated pit as in *Mancalla vegrandis* sp. n. and *Mancalla californiensis* Lucas 1901 (121:1; Fig. 5); dorsal and ventral edges of scar extending into primary pneumotricipital fossa taper to a point as in *Mancalla vegrandis*, rather than remaining parallel as in *Mancalla californiensis* and *Mancalla cedrosensis* (123:1); humerus longer than *Mancalla cedrosensis*, *Mancalla californiensis*,and *Mancalla vegrandis* (Tables 2, 3).

**Figure 5.**
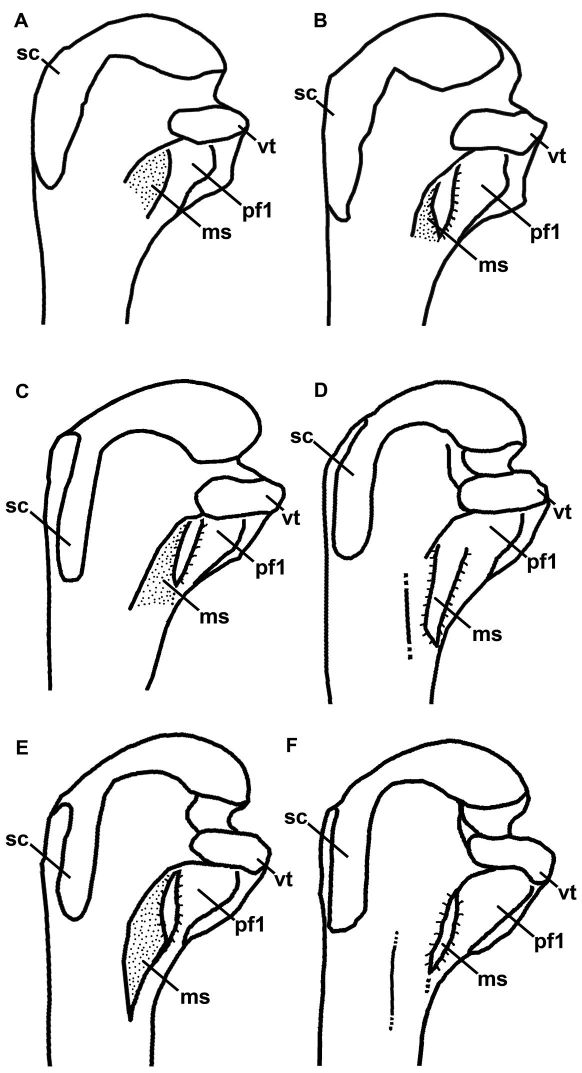
Line drawings for comparison of Mancallinae proximal humeri in posterior view (not to scale). **A** *Miomancalla wetmorei* **B** *Miomancalla howardi* **C** *Mancalla californiensis* **D** *Mancalla cedrosensis* **E** *Mancalla vegrandis* **F** *Mancalla lucasi*. Anatomical abbreviations: **ms** mancalline scar **pf1** primary pneumotricipital fossa **sc** supracoracoidal crest **vt** ventral tubercle.

##### Anatomical description.

Both scapulae are preserved (Fig. 3G, H). As in all Alcidae, the scapular shaft is mediolaterally compressed throughout its entire length. The proximal end of the scapular shaft is more rounded in other Charadriiformes. As in *Mancalla vegrandis*, the acromion projects farther anteriorly than that of *Mancalla cedrosensis* and other alcids (e.g., *Uria*, *Aethia*). As in *Mancalla cedrosensis*, the coracoidal tubercle is less pronounced than in *Mancalla vegrandis*. As in *Mancalla vegrandis* and *Mancalla cedrosensis*, a scapulotricipital tubercle is present just distal to the glenoid process on the ventral margin of the scapular shaft. This feature is also present in other flightless wing-propelled divers such as Spheniscidae and *Pinguinus*, but is not known in any volant alcid. As in *Mancalla vegrandis*, the scapular shaft, including the caudal extremity, is slightly more robust than in other alcids (e.g., *Alca*, *Aethia*). The caudal extremity is less dorsoventrally expanded than in *Mancalla vegrandis*. The caudal extremity is not known for *Mancalla cedrosensis*.

**Figure 6.**
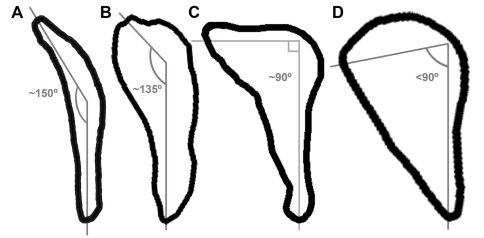
Comparison of sternal facet curvature in charadriiform left coracoids (sternal view; not to scale). **A** *Stercorarius* **B** *Mancalla* **C** *Alca* **D** *Aethia*.

Fragments of the sternum preserve the sternal rostrum, coracoidal sulci, and the carinal apex (Fig. 3A, B). These features are not preserved in *Miomancalla howardi* and comparisons are therefore limited to extant alcids and specimens of Mancallinae that are not presently referable to species. The morphology of the sternal rostrum is consistent with that of all other Alcidae. Although no coracoid is preserved in the holotype specimen of *Mancalla lucasi*, the shape of the coracoidal sulci of the sternum is consistent with the ~150° angle of the sternal articulation of the coracoid in *Mancalla cedrosensis* and *Mancalla vegrandis*. The sternal articulation of the coracoid, and the coracoidal sulci of the sternum in other alcids curves more acutely (e.g., ~90° in *Alca torda*; Fig. 6).

Complete right and left humeri are preserved (Fig. 3C, D, E and F). Based upon humeral proportions, *Mancalla lucasi* represents the largest known species of *Mancalla* (Table 2). As in other *Mancalla* species, the ventral margin of the ventral tubercle is convex, and the capital groove is relatively narrower than other Alcidae. The ventral tubercle does not project as far ventrally as in *Mancalla californiensis* (Fig. 5). The distal end of the deltopectoral crest transitions to the shaft more abruptly than in *Mancalla vegrandis*. As in other *Mancalla*, the humeral head is rotated anteriorly, and the supracoracoideus muscle scar does not broaden proximally. Mancallinae is characterized by a scar of unknown function that is positioned adjacent to the primary pneumotricipital fossa (hereafter referred to as the ‘mancalline scar’; Fig. 5). The position of the ‘mancalline scar’ suggests an accessory insertion of m. humerotriceps (Howard 1949), which can be divided into as many as four separate heads in some birds (Baumel and Witmer 1993). Other potentially homologous muscle scars include m. coracobrachialis cranialis, which is well developed in penguins (Ksepka et al. 2008), or m. scapulocranialis caudalis (see Matsuoka and Hasegawa 2007). However, the exact function of this feature is unknown because it is not present in any other charadriiform. The shape, position, and development of this scar is variable in Mancallinae (Fig. 5). The ‘mancalline scar’ of *Mancalla lucasi* is raised in relief like that of *Mancalla cedrosensis*, rather than excavated as in *Mancalla californiensis* and *Mancalla vegrandis* (Fig. 5). As in *Mancalla vegrandis*, the scar extends from a point just proximal to the junction of the bicipital crest with the humeral shaft, tapers to a point, and extends into the primary pneumotricipital fossa (Fig. 5). The dorsal and ventral margins of the ‘mancalline scar’ remain approximately parallel in *Mancalla californiensis* and *Mancalla cedrosensis* (Fig. 5). As in all Mancallinae, the humeral shaft is arced rather than sigmoidal or straight. As in other *Mancalla*, the dorsal supracondylar process is separated from the dorsal epicondyle by a small notch. A tubercle or papilla is present on the posterior side of the distal end of the humerus adjacent to the dorsal condyle (Howard 1976). As with all Mancallinae, the anterior surface of the ventral condyle is rounded, rather than flattened as in all other alcids. Rounded fossae are present at the proximal ends of the humerotricipital and scapulotricipital grooves. The flexor process extends distal to the ventral condyle as in all Mancallinae and *Pinguinus*.

The left femur is preserved (Fig. 3I) and is smaller (~15%; Table 2)than in *Miomancalla howardi* sp. n. (Table 3), and larger (~19%) than in *Mancalla cedrosensis* (Howard, 1971). Extant alcids do not display statistically significant degrees of sexual dimorphism in their size, plumage, or osteological morphology (Storer 1952; Nettleship and Birkhead 1985; Szekely et al. 2000). Thus, it can be reasonably assumed that extinct alcids were also not sexually dimorphic as the proposed sister taxon of all alcids, the Stercorariidae (Ericson et al. 2003; Thomas et al. 2004; Baker et al. 2007; Pereira and Baker 2008), as well as the closely related Laridae are also not sexually dimorphic. This range of size between *Mancalla* species is greater than the range of intraspecific variation documented for other alcids (~1-5%), including the flightless Great Auk (Moen 1991; Burness and Montevecchi 1992). As in *Alle*, *Cepphus* Mörhing, 1758, *Synthliboramphus* Brandt, 1837, and *Brachyramphus* Brandt, 1837, the femoral trochanter projects anteriorly in lateral view. The femoral trochanter in *Uria*, *Aethia* Merrem, 1788, *Alca*, and *Pinguinus* is not projected anteriorly (i.e., straight), and the trochanter is concave in lateral view in *Fratercula* Brisson, 1760 and *Cerorhinca* Bonaparte, 1828. Femora of *Miocepphus* are not known. No diagnostic characteristics of the femur of *Mancalla lucasi* were identified.

##### Remarks.

*Mancalla lucasi* corresponds in size and some humeral characteristics with material previously referred to *Mancalla diegensis*. However, *Mancalla diegensis* is considered Alcidae incertae sedis (see Appendix 1 for details of the taxonomic revision).

#### Mancalla vegrandis sp. n.

urn:lsid:zoobank.org:act:8F6D55BF-C827-47C3-AAB6-777632C92DB6

##### Holotype.

**Figure 7.**
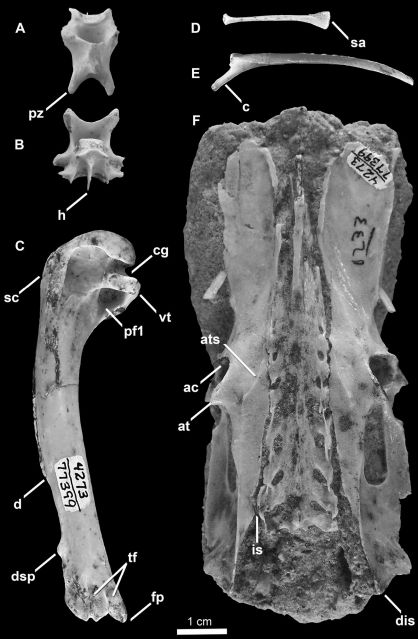
Holotype specimen of *Mancalla vegrandis* (SDSNH 77399) **A** Cervical vertebra (C3?) in dorsal view **B** Cervical vertebra (C4?) in ventral view **C** Left humerus in posterior view **D** Costal rib **E** Vertebral rib **F** Pelvis in dorsal view. Anatomical abbreviations: **ac** acetabulum **at** antitrochanter **ats** antitrochanteral sulcus **c** capitulum of vertebral rib **cg** capital groove **d** deltopectoral crest **dis** dorsal illiac spine **dsp** dorsal supracondylar process **fp** flexor process **h** hypapophysis **is** iliosynsacral suture **pf1** primary pneumotricipital fossa **pz** postzygapophysis **sa** sternal articulation of costal rib **sc** supracoracoidal crest **tf** tricipital fossae **vt** ventral tubercle.

**Figure 8.**
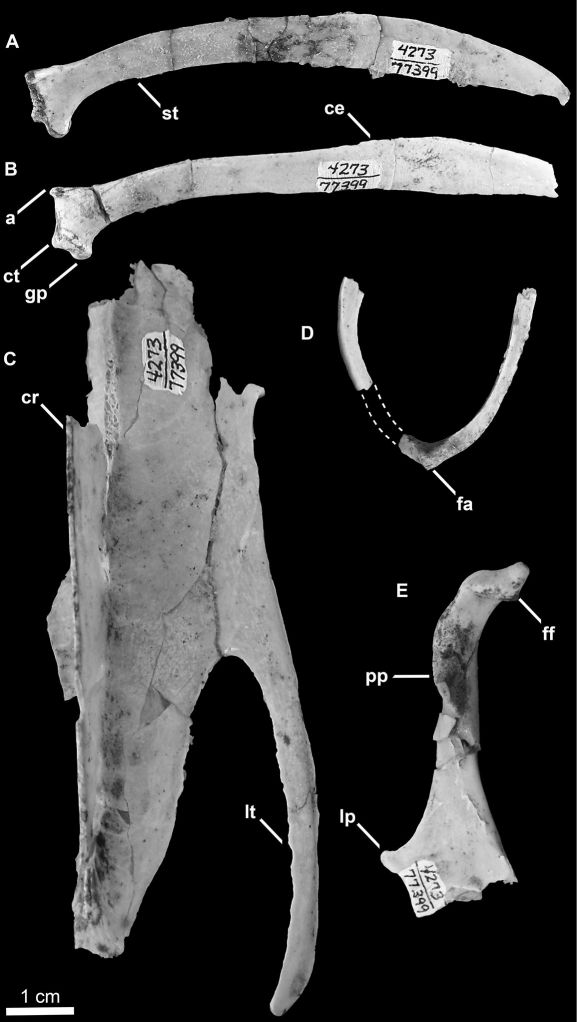
Holotype specimen of *Mancalla vegrandis* (SDSNH 77399). **A** Right scapula in medial view **B** Left scapula in lateral view **C** Partial sternum in ventral view **D** Partial furcula in posterior view (dashed lines represent missing portion of left ramus) **E** Left coracoid in posterior view. Anatomical abbreviations: **a** acromion process **ce** caudal extremity of scapula **cr** sternal carina **ct** coracoidal tubercle **fa** furcular apophysis **ff** furcular facet of coracoid **gp** glenoid process **lp** latral process of coracoid **lt** lateral trabeculae of sternum **pp** procoracoid process **st** scapulotricipital tubercle.

SDSNH 77399: a partial postcranial skeleton comprising the following elements: two cervical vertebrae, one costal and one vertebral rib, partial furcula, scapulae, left coracoid, partial right coracoid, partial sternum, left humerus, and pelvis (Figs 7 and 8; Tables1, 2 and 3). The holotype specimen was collected by W. T. Stein in October, 1961.

##### Etymology.

The species name *vegrandis* reflects the diminutive size of this taxon compared to other known *Mancalla* species (vegrandis, from the Latin for small, diminutive or tiny).

##### Locality and horizon.

Middle Pliocene to Early Pleistocene (Zanclean-Calabrian) San Diego Formation of San Diego County, California. Latitude, longitude, and elevation data are on file at SDSNH (locality 4273). Details of the geologic setting are provided in Appendix 6.

##### Referred specimens.

SDSNH 42532: a complete left humerus from the Middle Pliocene to Early Pleistocene San Diego Formation of San Diego County, California (SDSNH locality 3468); SDSNH 42534: a complete right humerus from the Middle Pliocene to Early Pleistocene San Diego Formation of San Diego County, California (SDSNH locality 3468); SDSNH 28152: a complete right humerus from the Early Pliocene upper member of the San Mateo Formation of San Diego County, California (SDSNH locality 3161); SDSNH 75051: a complete right humerus from the Early Pliocene upper member of the San Mateo Formation of San Diego County, California (SDSNH locality 2643; Fig. 4A–D).

##### Differential diagnosis.

Dorsal and ventral edges of the mancalline scar extending into primary pneumotricipital fossa taper to a point as in *Mancalla lucasi*, rather than remaining parallel as in *Mancalla californiensis* and *Mancalla cedrosensis* (123:1; Fig. 5); mancalline scar extending into primary pneumotricipital fossa is an excavated pit as in *Mancalla californiensis* rather than raised in relief to the floor of the primary pneumotricipital fossa and the humeral shaft as in *Mancalla cedrosensis* and *Mancalla lucasi* (121:0); humerus shorter than other known *Mancalla* (Tables 2 and 3).

##### Anatomical description.

Two cervical vertebrae are preserved (Fig. 7A and B). Comparisons with *Miomancalla howardi* are limited to generalities regarding shape in dorsal view, for which the morphology of *Mancalla vegrandis* is consistent with that of *Miomancalla howardi*. Only thoracic vertebrae are known for *Mancalla cedrosensis*. One of the vertebrae (Fig. 7A) is mediolaterally narrower than the other (Fig. 7B). Although the width of cervical vertebrae other than the axis and atlas do not vary considerably in extant Alcidae, the 3rd and 4th cervical vertebrae of some charadriiforms (e.g., *Larosterna inca* Lesson, 1827) are mediolaterally narrower than cervical vertebra posterior to the 4th (i.e., C5, C6, C7). The dorsal surface of the broader vertebra (Fig. 7B) is perforated by a small foramen (i.e., perforation of laminae arcocostales). In extant alcids, only the third and fourth cervical vertebrae are perforated. Typically in extant Alcidae, the third cervical vertebra is punctured by a small foramina, whereas the foramina in the fourth cervical vertebra is much larger, leaving only a thin strut of bone bordering it laterally. The morphology of the preserved vertebrae is suggestive of C3 and C4; however, definitive assignment cannot be made at this time.

One complete cervical rib and one complete costal rib (Fig. 7D and E) are preserved along with several other rib fragments (not figured). No morphological differences were evident between the ribs of *Mancalla vegrandis*, Mancallinae specimen SDSNH 25236,and other alcids for which the ribs are known.

All but the omal extremities of the furcula are preserved (Fig. 8D). The furcular rami are mediolaterally compressed as in all other Alcidae. The anterior surface of the furcular rami dorsal to the apophysis is rounded or convex as in *Uria*, rather than grooved as in *Cepphus*. The furcular apophysis does not bear the ventrally expanded, bladelike interclavicular process characteristic of extant Alcidae. However, the possibility that this feature was lost to damage cannot be ruled out. No additional morphological differences were evident between the preserved portions of the furcula of *Mancalla vegrandis* and other alcids for which the furcula is known.

The left coracoid is complete except for a small portion of the medial margin of the sternal facet (Fig. 8E). A fragment of the right coracoid preserves the medial margin of the sternal facet and the sternal portion of the coracoidal shaft (not figured). As in *Mancalla cedrosensis* the furcular facet is rounded, rather than oval as in *Aethia* and *Fratercula*. The head of the coracoid is apneumatic as in all Alcidae, but the brachial tuberosity is deeply undercut as in *Alca* and *Pinguinus*. The humeral articulation is more rounded than in extant Alcidae. As in *Cepphus*, the scar marking the position of m. supracoracoideus is less distinct than in other Alcidae. As in *Mancalla cedrosensis*, *Aethia*, and *Alle*,the procoracoidal process is not punctured by a foramen for passage of the tendon of m. supracoracoideus. The procoracoid process points dorsomedially as in all Alcidae except *Aethia*, in which the procoracoid points more ventromedially. As in *Mancalla cedrosensis*, *Brachyramphus*, *Uria*, *Aethia*, and *Ptychoramphus* Brandt, 1837, the sternal margin of the procoracoid process is concave, rather than convex as in *Cerorhinca*, *Fratercula,* and *Pinguinus*. As in many alcids (e.g., *Alca*, *Brachyramphus*) a single, distinct, straight ridge, which extends from the lateral angle of the sternal facet towards the humeral facet is present. This ridge does not extend sternally in *Synthliboramphus*, *Cepphus*, *Fratercula*, *Aethia*, *Ptychoramphus*, and *Cerorhinca*. This ridge is less pronounced and positioned farther laterally in *Mancalla cedrosensis*. A well-developed lateral process is present. This feature is absent in *Mancalla cedrosensis*. The dorsal margin of the medial sternal process is notched as in most alcids (e.g., *Alca torda*). As in *Mancalla cedrosensis*, the posterior surface of the sternal end of the coracoid is more excavated than in extant Alcidae and the sternal facet is curved ~150°.

Right and left scapulae are preserved (Fig. 8A and B). As in all Alcidae, the scapular shaft is mediolaterally compressed throughout its entire length. As in *Mancalla lucasi*, the acromion projects farther anteriorly than that of other alcids (e.g., *Uria*, *Aethia*). The acromion of *Mancalla cedrosensis* does not project as far anteriorly as that of *Mancalla vegrandis*. The coracoidal tubercle is more pronounced than in *Mancalla lucasi* and *Mancalla cedrosensis*. As in *Mancalla lucasi* and *Mancalla cedrosensis*, a scapulotricipital tubercle is present just distal to the glenoid process on the ventral margin of the scapular shaft. As in *Mancalla lucasi*, the scapular shaft, including the caudal extremity, is slightly more robust than in other alcids (e.g., *Alca*, *Aethia*). The caudal extremity is more dorsoventrally expanded than in *Mancalla lucasi*. The caudal extremity is not known for *Mancalla cedrosensis*.

**Figure 9.**
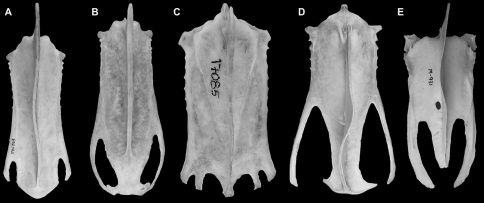
Comparison of charadriiform and sphenisciform sterni. **A** *Alca torda* (USNM 502382) **B** *Aethia psittacula* (NCSM 18514) **C** *Sterna anaethetus* (NCSM 17085) **D** *Hydrophasianus chirurgus* (USNM 490566) **E** *Eudyptula minor* (TMM M-391).

Parts of the left distal end of the sternum including the distal end of the carina, and the left lateral process are preserved (Fig. 8C). *Mancalla lucasi* and *Miomancalla howardi* do not preserve the same portions of the sternum so comparisons cannot presently be made between the sterni of Mancallinae. As a result of the deep incisure of the lateral notches the lateral processes of *Mancalla vegrandis* are more elongate that any other alcids for which the sternum is known. In other Charadriiformes this condition is present only in the Glareolidae and Scolpacidae, and resembles the sternum in Spheniscidae (Fig. 9).

The left humerus is preserved (Fig. 7C). Based upon humeral proportions, *Mancalla vegrandis* represents the smallest known species of *Mancalla* (Table 2). As in other species of *Mancalla*, the ventral margin of the ventral tubercle is convex, and the capital groove is relatively narrower than other Alcidae. The ventral tubercle does not project as far ventrally as in *Mancalla californiensis*. The distal end of the deltopectoral crest transitions to the shaft less abruptly than in *Mancalla lucasi*. As in other Mancallinae, the humeral head is rotated anteriorly and the supracoracoideus muscle scar does not broaden proximally. The ‘mancalline scar’ is excavated as in *Mancalla californiensis*, rather than raised in relief like that of *Mancalla cedrosensis* and *Mancalla lucasi* (Fig. 5). As in *Mancalla lucasi*, the ‘mancalline scar’ extends from a point just proximal to the junction of the bicipital crest with the humeral shaft and tapers to a point, and extends into the primary pneumotricipital fossa. The margins of this scar remain parallel in *Mancalla californiensis* and *Mancalla cedrosensis*. As in all Mancallinae, the humeral shaft is arced rather than sigmoidal or straight. As in other *Mancalla*, the dorsal supracondylar tubercle is separated from the dorsal epicondyle by a small notch. A tubercle or papilla is present on the posterior side of the distal end of the humerus adjacent to the dorsal condyle (Howard, 1966). As with all Mancallinae, the anterior surface of the ventral condyle is rounded, rather than flattened as in all other Alcidae. Rounded fossae are present at the proximal ends of the humerotricipital and scapulotricipital grooves. The flexor process extends distal to the ventral condyle as in all Mancallinae and *Pinguinus*.

The pelvis is preserved in dorsal view (Fig. 7F). Comparisons of pelves within Mancallinae are limited to *Miomancalla howardi*. As in all alcids the anteroposterior length of the pelvis is greater than two times the mediolateral width across the antitrochanters. The relative length of the pelves of other charadriiforms is anteroposteriorly shorter. The proximal end of the preacetabular ilium is wide as in *Miomancalla howardi* and most alcids (e.g., *Brachyramphus*). The distal end of the preacetabular ilium is relatively broader than in *Miomancalla howardi*. As in *Miomancalla howardi* the antitrochanteral sulcus does not extend proximally to contact the antitrochanter. As in most Alcidae (e.g., *Brachyramphus*), the post-acetabular dorsal ilium narrows, rather than broadens as in *Uria*, *Cepphus*, and some Fraterculinae. The iliosynsacral suture is perforated as in *Uria*, *Alca*, *Pinguinus*, and *Synthliboramphus*, rather than fused along its entire length as in *Cepphus*, *Brachyramphus,* and Fraterculinae. The dorsal iliac spine has a pointed tip as in all alcids other than *Aethia* and *Ptychoramphus*, in which the end of the spine is blunt.

##### Remarks.

*Mancalla vegrandis* corresponds in size and humeral characteristics with some material previously referred to *Mancalla milleri* Howard, 1970. However, *Mancalla milleri* is considered Alcidae incertae sedis (see Appendix 1 for details of the taxonomic revision).

#### Miomancalla gen n.

urn:lsid:zoobank.org:act:6280FCDF-06BA-46F8-A795-3AFF52A5A001

##### Type species.

*Miomancalla howardi* sp. n.

##### Etymology.

*Mio* to reflect Miocene occurrences of known species within the taxon, and *mancalla* to reflect the sister group relationship with *Mancalla* Lucas, 1901.

##### Differential diagnosis.

*Miomancalla* is differentiated from *Mancalla* by the following humeral characteristics: capital groove wider (137:0); supracoracoidial crest (sensu Fürbringer 1888; see Baumel and Witmer 1993: 98) proximally broader (113:1); ventral margin of the ventral tubercle broader and deeply grooved rather than narrow and ventrally expanded (134:1); distal margin of the primary pneumotricipital fossa concave rather than convex (126:2).

##### Remarks.

Based upon phylogenetic results (see below) and apomorphies shared with *Miomancalla howardi* (see diagnosis above), *Praemancalla wetmorei* Howard 1966is referred to *Miomancalla*, and becomes *Miomancalla wetmorei* (Howard 1966). See Appendix 1 for additional details of the taxonomic revision.

#### Miomancalla howardi sp. n.

urn:lsid:zoobank.org:act:BF31D07E-0CFF-4202-BE8C-C5E97F49F625

##### Holotype.

**Figure 10.**
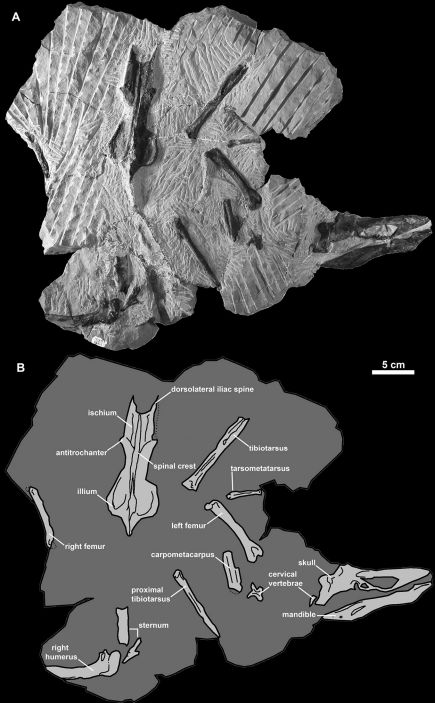
Holotype specimen of *Miomancalla howardi* (SDSNH 68312). **A** Photograph with contrast digitally adjusted to better display bone against similarly colored matrix **B** Line drawing of holotype specimen showing position of preserved elements with bones in light grey and matrix in dark grey.

**Figure 11.**
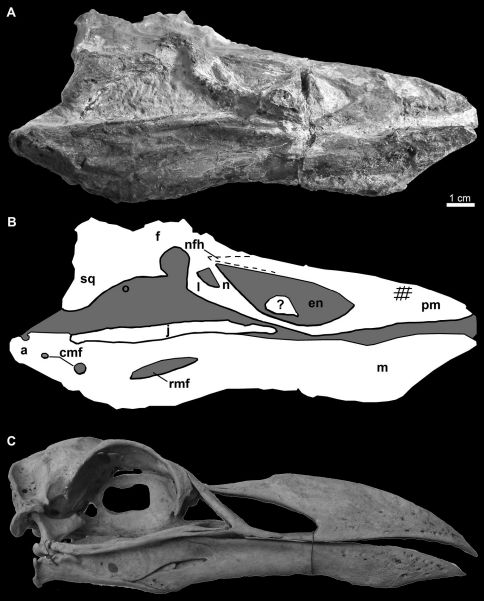
Photograph **A** and line drawing **B** of the skull of *Miomancalla howardi* compared with the skull of *Pinguinus impennis* (**C**; not to scale; USNM 346387). Cross-hatched lines on the premaxilla represent abrasion and dotted lines represent approximate reconstruction of incomplete elements. Anatomical abbreviations: **a** articular **cmf** caudal mandibular fenestrae **en** external nares **f** frontal **j** jugal **l** lacrimal **m** mandible **n** nasal **pm** premaxilla **nfh** nasofrontal hinge **o** orbit **rmf** rostral mandibular fenestra **sq** squamosal; **?** unidentified bone fragment.

SDSNH 68312: a partial skeleton collected by B. O. Riney on May 31, 1990 and comprising the following elements: partial skull, mandible, two cervical vertebrae, partial sternum, partial right humerus, left carpometacarpus, pelvis, femora, tibiotarsi, left tarsometatarsus (Figs 10, 11; Tables 1, 2 and 3).

##### Etymology.

This new species is named in honor of Hildegarde Howard in recognition of her many contributions to the systematics of extinct Alcidae.

##### Locality and horizon.

Early Pliocene (Zanclean; Deméré and Berta 2005) upper siltstone member of the Capistrano Formation, San Clemente, Orange County, California. Latitude, longitude and elevation data on file at SDSNH (locality 4160). Details of the geologic setting are provided in Appendix 6.

##### Referred specimen.

**Figure 12.**
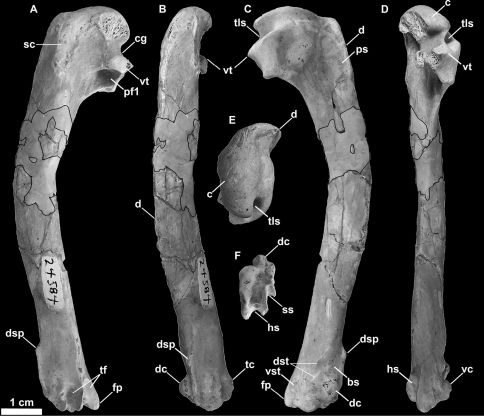
Referred left humerus of *Miomancalla howardi* (SDSNH 24584; dark outlined areas represent reconstructed areas obscured by repair). **A** posterior view **B** dorsal view **C** anterior view **D** ventral view **E** proximal view **F** distal view. Anatomical abbreviations: **bs** brachialis scar **c** caput **cg** capital groove **d** deltopectoral crest **dc** dorsal condyle **dsp** dorsal supracondylar process **dst** dorsal supracondylar tubercles **fp** flexor process **hs** humerotricipital sulcus **pf1** primary pneumotricipital fossa **ps** pectoralis scar **sc** supracoracoidal crest **ss** scapulotricipital sulcus **tc** tricipital crest **tf** tricipital fossae **tls** transverse ligament sulcus **vc** ventral condyle **vst** ventral supracondylar tubercle **vc** ventral condyle **vst** ventral supracondylar tubercle **vt** ventral tubercle.

SDSNH 24584, a left humerus (Fig. 12) from the Late Miocenelower member(Messinian) of the San Mateo Formation of San Diego County, California (SDSNH locality 3177). This specimen was noted but not named or described by Chandler (1985) and Livezey (1988).

##### Differential diagnosis.

Differs from *Miomancalla wetmorei* in the following characteristics: ventral margin of ventral tubercle more deeply grooved; transverse ligament furrow deeper, with lateral lip extended farther medially; capital groove wider, and flatter; dorsal supracondylar process less dorsally projected; groove between dorsal supracondylar process and dorsal condyle wider; ventral supracondylar tubercle more prominent; tubercle present proximal to dorsal condyle as in *Mancalla cedrosensis* (155:1); humerus ~20% longer (Table 2; Livezey 1988, Fig. 3A).

##### Anatomical description.

The holotype specimen is preserved in a matrix of dark grey, highly indurated, siltstone (Fig. 10). Some elements areslightly crushed and many cortical bone surfaces are considerably abraded, obscuring fine morphological details in many portions of the specimen.

**Figure 13.**
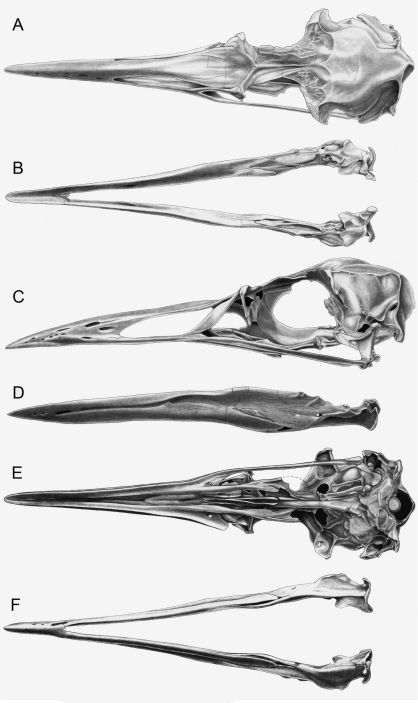
Skull of Mancallinae (SDSNH 25236). **A** Dorsal view of skull **B** Dorsal view of mandible **C** Left lateral view of skull **D** Left lateral view of mandible **E** Ventral view of skull **F** Ventral view of mandible (sketches by Michael Emerson).

Elements of the skull are exposed in oblique right lateral view (Figs 10, 11). The premaxilla, maxilla, nasal, lacrimal, jugal, frontal, and squamosal are present. Additional fragments of bone adjacent to the posterior frontal may represent a portion of the parietal. An unidentified fragment of bone protrudes from the external narial opening. The premaxilla is relatively shorter and mediolaterally compressed in comparison with the only other known premaxillae referable to Mancallinae (LACM 103940; SDSNH 25236; Fig. 13), which resemble the more terete bills of some other Alcidae (e.g., *Uria*). The maxilla, which broadens anteriorly before fusion with the premaxilla, is complete but broken at approximately its midpoint. As in many alcids (e.g., *Cepphus*, *Alca*) the nasal contacts the maxilla at ~45° angle. This angle is ~60° in the puffins and auklets (i.e., *Fratercula*, *Cerorhinca*, *Aethia*, and *Ptychoramphus*). As in *Pinguinus*, and in contrast to other alcids, the lacrimal appears to be directed ventrally rather than posteroventrally. However, crushing of the skull may have changed the relative orientation of elements and it is possible that distortion is responsible for this condition. The jugal is preserved in contact with the mandible. Fusion between the jugal and the jugal process of the premaxilla is visible. The frontal is distorted by crushing and most morphological details obscured in this element. The outline of the right orbit is visible, but is deformed by ventrolateral displacement of the lateral margin of the frontal. The frontal bears a robust orbital rim as in *Uria*, *Miocepphus*, *Alle*, *Alca*, and *Pinguinus*.

The mandible is preserved in right lateral view (Figs 10, 11). The mandibular symphysis is elongate as in *Uria* and *Fratercula*. The mandibular rami are fused along a relatively shorter distance in some alcids (e.g., *Alle*). The proximal and distal ends of the mandible are dorsoventrally expanded, similar to the condition in *Alca* and *Pinguinus*. A pair of small posterior mandibular fenestrae is present as in other known Mancallinae mandibles (LACM 103940; SDSNH 25236; Fig. 13), Fraterculini Storer, 1960, and some charadriiforms (e.g., *Stercorarius longicaudus* Vieillot, 1819).

At least two cervical vertebrae are partially exposed on the surface of the slab (Fig. 10). Fine morphological details are obscured by matrix and the poor preservation of the vertebrae. One vertebra resembles the axis, but positive identification is hindered by matrix and damage to the element. The other is a cervical vertebra exposed in dorsal view. Mancallinae vertebrae are known only from the holotype specimens of *Mancalla cedrosensis* and *Mancalla vegrandis*. Comparisons with *Mancalla cedrosensis* are not possible because only a single thoracic vertebra is preserved in the holotype specimen. The shape of the dorsal surface of the cervical vertebrae of *Miomancalla howardi* is consistent with that of *Mancalla vegrandis*. Further preparation of the holotype specimen of *Miomancalla howardi*,or discovery of additional material referable to this species is necessary before more details of vertebral anatomy can be described for this species.

Fragments of the sternum are preserved adjacent to the humerus in what appears to be ventral view (Fig. 10). The craniolateral process appears to point dorsally, rather than anteriorly as in *Mancalla lucasi*, although the possibility that crushing of this element altered the relative orientation of that feature cannot be ruled out. Other morphological details are obscured by matrix and the poor preservation of the sternum.

The holotype specimen preserves the proximal end of the right humerus in posterior view (Fig. 10). In addition to the head of the humerus, which is slightly crushed, the outline of the proximal half of the humeral shaft is visible as an impression in matrix. A complete left humerus (SDSNH 24584; Fig. 12) is referable to *Miomancalla howardi* based upon its similar proportions (i.e., larger than any other known Mancallinae; Table 2), and the fact that the ventral surface of ventral tubercle is more deeply grooved than in any other alcid. The ventral surface of the ventral tubercle is also grooved in *Pinguinus* and *Miomancalla wetmorei*, but the degree of excavation of this groove is more pronounced in *Miomancalla howardi*. The ventral margin of the ventral tubercle of *Mancalla* is convex. The capital groove is relatively wider than that of other species of Mancallinae, and it is incised more deeply into the transverse ligament sulcus in anterior view than in *Miomancalla wetmorei*. The proximal end of the deltopectoral crest is less pronounced than in *Miomancalla wetmorei*. The distal end of the deltopectoral crest transitions to the shaft less abruptly than in *Mancalla*. The humeral head is rotated more anteriorly than in *Miomancalla wetmorei*, and is more similar to the condition in *Mancalla*. As in *Miomancalla wetmorei* and *Fratercula*, and in contrast to the condition in *Mancalla* species, the supracoracoideus muscle scar broadens proximally. In *Miomancalla howardi* and *Miomancalla wetmorei* the ‘mancalline scar’ extends from a point just proximal to the junction of the bicipital crest with the humeral shaft and tapers to a point that meets the dorsal border of the primary pneumotricipital fossa (i.e., crus dorsale fossae of Baumel and Witmer 1993: 99). The scar is relatively smaller in *Miomancalla* and *Mancalla lucasi* thanin comparison with other Mancallinae. The scar is an excavation in all Mancallinae except *Mancalla cedrosensis* and *Mancalla lucasi*, in which the scar is raised in relief to the floor of the primary pneumotricipital fossa and the humeral shaft. The shaft of the humerus is arced more so than in *Miomancalla wetmorei* or any other known alcid, and is less dorsoventrally compressed than in *Pinguinus*. As in all alcids other than *Mancalla*, the dorsal supracondylar process is continuous with the dorsal epicondyle, rather than separated from it by a small notch. The dorsal supracondylar process is less pronounced than in *Miomancalla wetmorei*. A tubercle or papilla on the posterior side of the distal end of the humerus adjacent to the dorsal condyle was described by Howard (1966), who used that characteristic to differentiate between species of *Mancalla* that possessed the tubercle, and species of *Miomancalla* (*Praemancalla* sensu Howard, 1966) that did not posses it. The tubercle is present in *Miomancalla howardi*. As with all Mancallinae, the anterior surface of the ventral condyle is rounded, rather than flattened as in all other Alcidae. Rounded fossae are present at the proximal ends of the humerotricipital and scapulotricipital grooves. That character cannot be evaluated in *Miomancalla wetmorei* or *Mancalla californiensis* owing to damage to the holotype specimens of those species and current lack of referable specimens. The flexor process extends distal to the ventral condyle as in all Mancallinae and *Pinguinus*.

The left carpometacarpus is preserved in dorsal view (Fig. 10). Although hundreds of Mancallinae carpometacarpi are known from Pliocene marine deposits in California, the holotype specimens of *Miomancalla howardi* and *Mancalla cedrosensis* are the only associated specimens that allow for species-level referral of carpometacarpi. The carpometacarpus of *Miomancalla howardi* is larger than that of *Mancalla cedrosensis* (~23%; Table 3; Howard 1971), and displays the distal elongation of metacarpal I that is characteristic of Mancallinae. The abraded preservation of this element limits further comparisons.

The pelvis is exposed in dorsal view (Fig. 10). Comparisons within Mancallinae are limited to *Mancalla vegrandis*. As in all alcids the anteroposterior length of the pelvis is greater than two times the mediolateral width across the antitrochanters. The relative length of the pelves of other charadriiforms is anteroposteriorly shorter. The proximal end of the preacetabular ilium is wide as in *Mancalla vegrandis* and most alcids (e.g., *Brachyramphus*). The distal end of the preacetabular ilium narrows more so than in *Mancalla vegrandis*. As with *Mancalla vegrandis* the antitrochanteral sulcus does not extend proximally to contact the antitrochanter. The dorsal iliac spine has a pointed tip as in all alcids other than *Aethia* and *Ptychoramphus*, in which the end of the spine is blunt.

The distal ends of both tibiotarsi are missing or embedded in matrix (Fig. 10). The poor preservation of these elements limits comparisons with the smaller holotype tibiotarsi of *Mancalla cedrosensis* to size (~26% larger; Table 3; Howard 1971).

The right femur is exposed in posterolateral view along the edge of the block but is severely abraded: however, the left femur is well-preserved and exposed in anterior view (Fig. 10). The femur is robust and less sigmoidal in shape in comparison with the femora of extant alcids such as *Alle* or *Uria*, resembling the condition in *Mancalla lucasi* and *Mancalla cedrosensis*, the only other Mancallinae from which the femur is known. The intercondylar sulcus is relatively broader and more well-defined proximally than that of *Mancalla lucasi* and *Mancalla cedrosensis*. As in *Cepphus*, *Brachyramphus*, and *Synthliboramphus*, the distally extending and anteriorly projected crest of the femoral trochanter is convex in shape. This feature is flattened (e.g., *Alca* and *Uria*) or concave (e.g., *Fratercula* and *Cerorhinca*) in other alcids. The femoral head appears relatively smaller in comparison with this element in *Mancalla cedrosensis* and *Mancalla lucasi*. The length of the femur is greater than in *Mancalla cedrosensis* and *Mancalla lucasi* (Table 3; Howard 1971).

The left tarsometatarsus is preserved in anterior view (Fig. 10). The anterior surface of the shaft is deeply grooved as in *Mancalla cedrosensis* and *Fratercula*. Associated specimens with tarsometatarsi that would allow for referral of isolated tarsometatarsi to species are not currently known from other Mancallinae. The outlines of trochlea are visible but the distal end of the element is too badly abraded to discern fine morphological details.

## Phylogenetic results

Owing to the incomplete and fragmentary preservation of most Mancallinae specimens referable to species, preliminary analysis of the systematic relationships of *Mancalla* resulted in an unresolved polytomy among Alcidae sub-clades (i.e., relationships between Mancallinae, *Cepphus*, *Brachyramphus*, *Synthliboramphus*, Alcini, and Fraterculinae (contents = Fraterculini Storer, 1960 + Aethiini Storer, 1960) unresolved at the base of a monophyletic alcid clade (results not shown). Two additional phylogenetic analyses were performed to investigate the position of Mancallinae within Charadriiformes, and the interrelationships of Mancallinae species. The primary phylogenetic analysis included a Mancallinae supraspecific terminal (SST) constructed by combining scorings from 19 Mancallinae specimens (including all holotype material; Appendix 4). The referral of all Mancallinae specimens used to construct the SST was evaluated based upon the unambiguously optimized apomorphies listed in the diagnosis section for Mancallinae above. Note that due to damage or missing elements in Mancallinae holotype specimens, five of the specimens used to construct the Mancallinae supraspecific terminal preserve morphological data not preserved by the holotype specimens, thus providing a more compete picture of morphological variation in Mancallinae than if only the holotype specimens were analyzed. The results of the first analysis were used to constrain the topology of trees accepted during a secondary tree search in which the species-level relationships of Mancallinae were evaluated.

**Figure 14.**
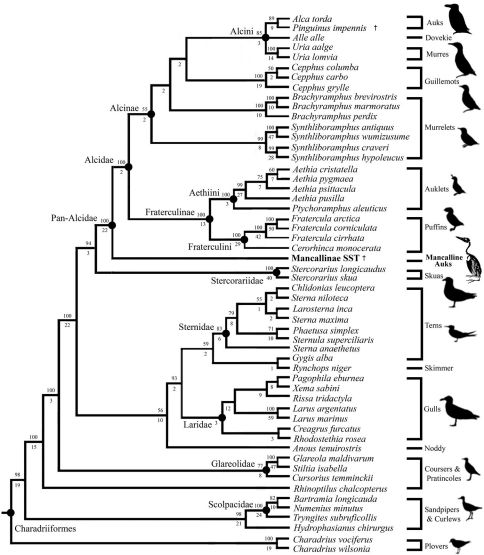
Results of primary phylogenetic analysis including the Mancallinae SST (2 MPT’s; TL: 15,974; CI O.38; RI O.50; RCI 0.19). Bootstrap values (>50%) are displayed above nodes, and Bremer support values are displayed below nodes.

The primary combined phylogenetic analysis of the cladistic matrix including a Mancallinae SST resulted in two most parsimonious trees (MPT’s) of 15,974 steps (Fig. 14; CI: 0.38; RI: O.50; RCI: 0.19). Additional analyses performed with all characters unordered did not result in topological differences, or an increase in the number of MPT’s recovered. Pan-Alcidae is recovered as the sister to Stercorariidae, a result that is congruent with the results ofprevious molecular based analyses(Ericson et al. 2003; Paton et al. 2003; Thomas et al. 2004; Paton and Baker 2006; Baker et al. 2007), but conflicts with previous morphology-based analyses (Strauch 1978; Björklund 1994; Chu 1995, 1998; Chu et al. 2009; Livezey 2009, 2010; Mayr 2011). Alcidae and Stercorariidae have not been recovered as sister taxa in any previous morphology based analysis, suggesting that molecular sequence data is solely responsible for this hypothesis. There is, however, morphological character support for this clade (Table 4). The combined analysis estimate of relationships among the Alcidae crown clade is congruent with the results of recent analyses of molecular sequence data (Thomas et al. 2004; Paton et al. 2003; Baker et al. 2007; Pereira and Baker 2008), except that *Synthliboramphus* is placed at the base of Alcinae, rather than as the sister to Alcini (Fig. 14). However, the parsimony analysis by Pereira and Baker (2008) also recovered *Synthliboramphus* at the base of Alcinae in one of two most-parsimonious topologies. The positions of other species (e.g., *Alca* + *Pinguinus*), and sub-clades in Alcidae (e.g., Fraterculinae + Alcinae) are consistent with the results of recent molecular-based analyses (Baker et al. 2007; Pereira and Baker 2008) with dense taxonomic sampling for Alcidae. The only prior morphology-based analyses with sufficient taxonomic sampling for comparison to these results, those by Strauch (1985) and Chandler (1990a), resulted in topologies that strongly conflict with more recent hypotheses of alcid relationships in that they do not support a traditional Fraterculinae (i.e., monophyly of Fraterculini + Aethiini). The Aethiini (i.e., *Ptychoramphus* + *Aethia*) are placed basal to the Alcinae (*Alca* + *Pinguinus* + *Cepphus* + *Brachyramphus* + *Synthliboramphus*), rather than as sister to the Fraterculini (i.e., *Cerorhinca* + *Fratercula*) in the topology of Strauch (1985). Although the work by Chandler (1990a) represented an increase in the number of characters scored for Alcidae, the results of that analysis placed *Alle alle* and *Cepphus* in a clade with the Fraterculini, rather than in Alcinae. The combined analysis, as well as previous analyses (Watada 1987; Moum et al. 1994; Friesen et al. 1996; Thomas et al. 2004; Baker et al. 2007; Pereira and Baker 2008) strongly support monophyly of a Fraterculinae clade consisting of *Ptychoramphus*, *Aethia*, *Cerorhinca*, and *Fratercula*, and the sister-group relationship between Fraterculinae and Alcinae as defined here (Fig. 14).

**Table 4.** Unambiguously optimized morphological characters with a CI of 1.0 supporting alcid clades in the resultant phylogenetic tree (Fig. 15). Character numbers from Appendix 3 are followed by character state symbols (e.g., 23:0 = character number 23, state 0). ‘*’ indicates selected locally optimized apomorphies with a CI of < 1.0.

*Clade*	*Character numbers and states that support monophyly*
Pan-Alcidae + Stercorariidae	*63:0; *124:1; *190:1; 315:1; 343:1
Pan-Alcidae	35:0; 38:1; 75:1; 77:1; 162:1
Alcidae (crown clade)	68:1; 153:1; 172:1
Alcinae	49:1; 270:1; 281:1
Alcini	185:1; 237:1; 239:1; 274:1
Fraterculinae	*10:1; *13:0; *52:1; *67:1; *72:1
Fraterculini	29:1; 35:1; 40:1; 63:1; 112:0; 275:1; 286:1; 287:1
Aethiini	11:1; 86:0; 94:1; 201:1
Mancallinae	104:2; 120:1; 139:1; 140:1; 148:1; 150:1; 183:0; 184:1
*Mancalla*	*130:1; 137:1

Only the systematic position of *Alle alle* Link, 1806 remains unresolved within Alcini (Fig. 14). The systematic position of *Alle alle* is potentially the most contentious issue within alcid systematics, as it has been recovered as the sister to *Alca* + *Pinguinus* (Moum et al. 1994, 2002; Baker et al. 2007), sister to *Alca* + *Pinguinus* + *Uria* (Strauch 1985), sister to *Uria* (Thomas et al. 2004; Pereira and Baker 2008), sister to Fraterculinae (Chandler 1990a), and sister to *Cepphus* + *Aethia* + *Brachyramphus* (Chu 1998). Resolution of this issue will likely require a comprehensive analysis of alcid relationships including dense taxonomic sampling of extinct Alcidae.

Mancallinae is placed as the sister taxon to all other Alcidae (i.e., placed outside of crown clade Alcidae; Fig. 14). This result is consistent with the only previous analysis that included Mancallinae (Chandler 1990a). The clade composed of crown Alcidae + Mancallinae is therefore designated Pan-Alcidae. The monophyly of Pan-Alcidae is supported by fiveunambiguously optimized morphological characters with a CI = 1.0 (UOMC; Table 4).

The combined analysis recovered relationships among the 29 charadriiform outgroup taxa that are largely congruent with prior molecular-based analyses of the clade, but do not support previous morphology-based results. *Larus* and *Hydrophasianus* (i.e., gulls and jacanas) are recovered as more closely related to one another than either are to *Charadriius* (i.e., plovers), as in the results obtained by Hackett et al. (2008). Also consistent with the results of prior molecular analyses (Ericson et al. 2003; Paton et al. 2003; Paton and Baker 2006; Baker et al. 2007), Alcidae + Stercorariidae is placed as the sister to Laridae + Sternidae + Rynchopidae. In contrast to the combined analysis results presented herein and recent molecular based results, the results of the phylogenetic analyses of morphological data by (Livezey and Zusi (2006, 2007) and (Livezey (2009, 2010) place Alcidae as the sister taxon to Stercorariidae + Rynchopidae + Laridae. However, taxon sampling for Alcidae was limited to *Uria* in the analysis of (Livezey and Zusi (2006, 2007), and Alcidae was included as a single, taxon level terminal in the analyses of (Livezey (2009, 2010) and Mayr (2011). The morphology based phylogeny of Mayr (2011) placed Alcidae in a polytomy with Dromadidae, Stercorariidae, and a clade comprising Laridae + Sternidae + Rynchopidae. The results of the combined analysis are congruent with recent molecular-based analyses, which place Lari (e.g., alcids, gulls, and pratincoles) as the sister to Scolpaci (e.g., sandpipers and curlews), and place Charadri (e.g., plovers), at the base of Charadriiformes. This hypothesis contrasts with morphology-based results (Björklund 1994; Chu 1995), which were the result of parsimony-based re-analyses of the compatibility analysis of Strauch (1978). In the topology recovered by Björklund (1994) the Charadri and Scolpaci are placed in an unresolved polytomy basal to the Lari, whereas the Lari and Charadri are placed in an unresolved polytomy basal to the Scolpaci in the topology recovered by Chu (1995). The morphology based analyses of (Livezey (2009, 2010) and Mayr (2011) recover Scolpaci as an outgroup to a Charadri + Lari clade. The contents of Charadri, Scolpaci, and Lari estimated by the combined analysis are consistent with the composition of those clades recovered in prior molecular-based phylogenetic analyses (Sibley and Ahlquist 1990; Paton et al. 2003; Ericson et al. 2003; Paton and Baker 2006; Baker et al. 2007), supporting the monophyly of Charadri, Lari, and Scolpaci. An additional combined analysis was performed in which the tree was *a priori* rooted with the Scolpaci clade (i.e., *Hydrophasianus*, *Tryngites*, *Numenius*, and *Bartramia*) to mimic the phylogenetic results of (Livezey (2009, 2010) and Mayr (2011). This alternative rooting scheme did not affect relationships recovered among Alcidae or Lari species and clades, between Alcidae and Stercorariidae, or between Mancallinae and other alcids (results not shown).

Also of interest is the placement of *Rynchops* (i.e., skimmers). Recent molecular analyses recovered *Rynchops* as the sister to Laridae (Paton et al. 2003; Baker et al. 2007) or sister to Sternidae (Paton and Baker 2006). The morphology-based analyses by (Chu (1995, 1998) placed *Rynchops* as the sister to Sternidae + Laridae + Stercorariidae. The results of the combined analysis place the Black Skimmer *Rynchops niger* Linnaeus, 1758 as the sister taxon to the White Tern *Gygis alba* Sparrman, 1786. Considering the accepted placement of *Gygis alba* in Sternidae (American Ornithologists’ Union 1998, Brigde et al., 2005), this result would suggest Sternidae paraphyly. Although, this result is not entirely novel because an alternative hypothesis also places *Gygis* outside Sternidae, as the sister to Laridae + Sternidae (Baker et al. 2007). However, denser taxonomic sampling of Rynchopidae, Sterndidae, and other Charadriiformes may resolve this issue in the future.

*Anous* (i.e., noddies) was recovered as the sister to Sternidae + Laridae + Rynchopidae in the combined analysis, a placement consistent with the molecular-based results reported by Baker et al. (2007), and in conflict with the morphology-based results obtained by Chu (1998), which place *Anous* as thesister to Stercorariidae. The only study with dense taxonomic sampling of terns and noddies (Bridge et al. 2005) included a single larid (*Larus delawarensis* Ord, 1815) as an outgroup taxon, but placed *Anous* basally in Sternidae. Resolution of the systematic affinities of *Anous* will likely require denser taxonomic and character sampling across Laridae, Sternidae, Rynchopidae, *Anous*, and other non-Lari charadriiforms.

**Figure 15.**
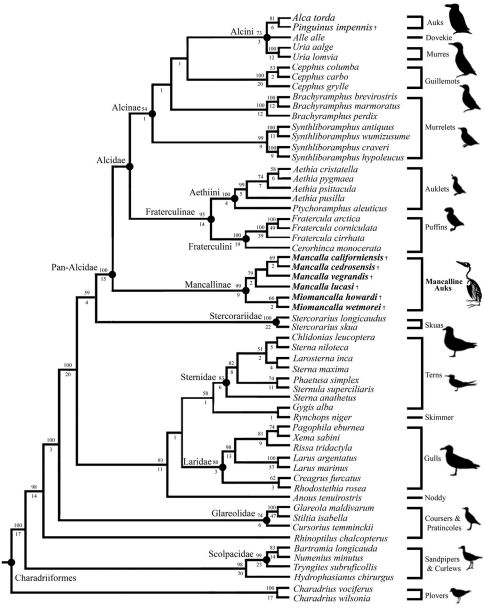
Results of secondary phylogenetic analysis of Mancallinae inter-relationships (2 MPT’s; TL: 15,971; CI O.38; RI O.51; RCI 0.19). Bootstrap values (>50%) are displayed above nodes, and Bremer support values are displayed below nodes.

The secondary phylogenetic analysis, which evaluated the interrelationships among Mancallinae resulted in two MPT’s of 15,971 steps (Fig. 15; CI: 0.37; RI: O.51; RCI: 0.19). Binary characters are interpreted as ambiguity (i.e., treated the same as ‘?’ scorings) when they are scored as polymorphic (e.g., 0&1 scorings), explaining the shorter tree length of the secondary analysis as compared to the primary analysis including the Mancallinae SST. The monophyly of Mancallinae is supported by eight UOMC’s (Table 4). *Miomancalla wetmorei* and *Miomancalla howardi* are placed as sister taxa, and *Miomancalla* monophyly is supported by three locally optimized morphological characters (LOMC; 105:0; 113:1; 134:1). *Miomancalla* is placed as the sister taxon to *Mancalla*. *Mancalla* monophyly is supported by one UOMC (137:1) and an additional LOMC (130:1). The placement of *Mancalla californiensis* as the sister taxon of *Mancalla cedrosensis* is supported by one UOMC (123:0), and an additional LOMC (109:1). *Mancalla vegrandis* and *Mancalla lucasi* are placed as successive outgroups to the clade composed of *Mancalla californiensis* and *Mancalla cedrosensis* (Fig. 15).

## Discussion

The taxonomic revision and description of new Mancallinae species herein confirms previous estimates of high diversity in Mancallinae (Howard 1970; Olson 1981; Chandler 1990a), and in combination with the phylogenetic results of the combined analysis, provide a new context for the interpretation of the evolutionary success of this lineage of flightless wing-propelled divers. Similar to the hypothesized independent evolution of flightlessness in penguins and plotopterids (Smith 2010), the placement of Mancallinae as the sister taxon to crown Alcidae suggests that flightlessness evolved independently in the Mancallinae and *Pinguinus* lineages, making the many osteological characteristics shared between these taxa an even more compelling example of morphological convergence. Phylogenetic support for the monophyly of *Miomancalla* and *Mancalla* also provides further contextualization for the interpretation of morphological differences between these sister taxa. Although known diversity is higher for *Mancalla*, there is an apparent trend towards decrease in size for more derived members of the clade, with the larger *Miomancalla* and *Mancalla lucasi* placed basally in the resultant topology (Fig. 15). Although it is tempting to infer large body-mass as the ancestral state for Pan-Alcidae, the reconstruction of this character is ambiguous according to the phylogenetic results, and there is an ~25Ma gap in the fossil record between the oldest known fossil alcid and the oldest Mancallinae fossils. The most important contributing factor regarding the ambiguity of ancestral states within Pan-Alcidae is the incompleteness of the early alcid fossil record. Although an abundance of taxa are known from the Miocene and Pliocene, only a single fragmentary alcid fossil is known form the Eocene (Chandler and Parmley 2002). The only Oligocene fossils that are currently referred to Alcidae are two fragmentary and isolated specimens from the Iwaki Formation in Japan (Ono and Hasegawa 1991). Eocene and Oligocene localities and collections should be targeted to increase knowledge of early diversity and ancestral states within Pan-Alcidae.

Although impressive with regard to the quantity of taxa sampled (*n* = 242) and the number of morphological characters scored for those taxa (*n* = 1107), comparisons with the results of a recent morphology based analysis of Charadriiformes (Livezey 2009, 2010) and the results of this study are limited to relationships among outgroup charadriiforms because Alcidae was included only as a suprageneric taxon. With respect to relationships among major charadriiform clades, some of the results of Livezey (2010) are admittedly in conflict with a growing consensus of molecular results based upon a variety of methods (e.g., parsimony, Bayesian) and sampling schemes (mitochondrial and nuclear DNA sequences). For example, although the placement of Charadriin a derived position within Charadriiformes to the exclusion of other clades (Livezey 2010) is in agreement with some previous hypotheses (Strauch 1978; Sibley and Ahlquist 1990; Christian et al. 1992; Björklund 1994; Chu 1995; Thomas et al. 2004; Livezey and Zusi 2007), these hypotheses are in contrast with the results of more recent multigene molecular based hypotheses that recovered Charadri in a more basal position. (Ericson et al. 2003; Paton et al. 2003; Paton and Baker 2006; Baker et al. 2007; Fain and Houde 2007; Hackett et al. 2008). There exists no metric with which to choose between the contrasting results of those many analyses, and thus systematic relationships between major clades of Charadiirformes remain somewhat uncertain. However, the combined analysis results reported herein represent the most inclusive analysis to date with respect to variety of phylogenetically informative data sampled.

### Referral of fossils to species level

Referral of specimens to named species, or recognition of new species, based solely upon size, or provenience, or age, or any combination of those three criteria, run the risk of incorrectly assigning specimens to species, or incorrectly assessing species diversity (Norell 1989; Stewart 2002b; Nesbitt and Stocker 2008; Bell et al. 2010). To avoid the possibility of recognizing two or more fossil species based upon different skeletal elements of the same species, recognition of new species must be predicated upon diagnoses or differentiation from previously named species within a taxon (Appendix 1). Occurrence within the same deposit or deposits of similar age is not considered strong evidence that fossils represent the same taxon. Similarly, a lack of recorded occurrences of a fossil taxon within a deposit or deposits of a particular age does not preclude the possibility that a taxon may have been extant during the time of deposition. For example, if the holotype specimen of a species is an isolated humerus, then only associated specimens with humeri consistent with that of the holotype specimen allow for initial referral of additional skeletal elements. When previously recognized holotype specimens consist of isolated elements, isolated material consisting of elements other than the holotype element cannot be referred to the species level until associated specimens are discovered that facilitate such referral. Although these criteria do not preclude the possibility that cryptic species may lead to underestimation of species diversity (see Stewart 2002b, 2007), these criteria do avoid overestimation of diversity and incorrect assignment of specimens that can result from less rigorous methods (i.e., size, provenience, or age based methods) of specimen referral and species recognition. In the case of Mancallinaeremains, there is little doubt that hundreds of isolated fossils are referable to that clade; however, to avoid future taxonomic confusion, referrals should only be made based upon the criteria outlined above. The morphological differences between Mancallinae holotype and referred specimens described and phylogenetically optimized herein provide a basis for the potential apomorphy-based referral of hundreds of additional isolated Mancallinae remains, which will facilitate future detailed study of interspecies morphologic and size variation in Mancallinae.

### Flightlessness and convergence

**Figure 16.**
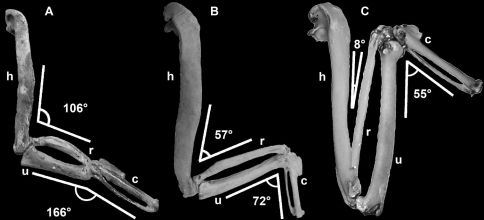
Wing elements of flightless and volant auks depicting decreased range of motion and shortening of distal wing elements. Elements not to scale and degree of flexion estimated based on manual articulation of specimens: **A** *Mancalla* (composite LACM 154560) **B** *Pinguinus impennis* (composite USNM 346387) **C** *Alca torda* (NCSM 20502). Anatomical abbreviations: **c** carpometacarpus **h** humerus **r** radius **u** ulna.

**Figure 17.**
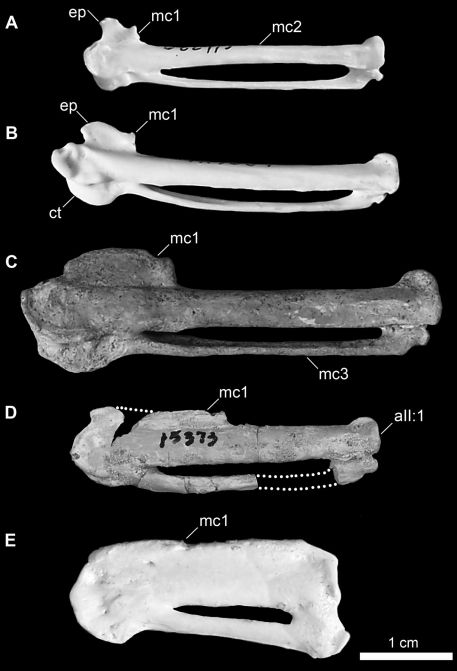
Comparison of charadriiform and sphenisciform carpometacarpi. **A** *Anous minutus* (USNM 622415) **B** *Cerorhinca monocerata* (USNM 620641) **C** *Pinguinus impennis* (USNM 623465) **D** *Mancalla cedrosensis* (LACM 15373) **E** *Eudyptula minor* (TMM M-931). Anatomical abbreviations: **aII**:**1** articulation of digit II phalanx 1 **ct** carpal trochlea **ep** extensor process **mc1** first metacarpal **mc2** second metacarpal **mc3** third metacarpal.

The etymology of *Mancalla* (*mancus*-from the Latin for crippled or lame, and *ala* from the Latin for wing; Brown 1956) reflects an antiquated view of flightlessness. The flightless condition observed in ostriches and some rails for example, in which the pectoral elements are diminished in size, has been attributed to lack of predatory pressures and energy conservation strategies (Livezey and Humphrey 1986; McNab 1994). The flightless condition observed in penguins, plotopterids and some auks (i.e., Mancallinaeand *Pinguinus*) reflects specialization for wing-propelled diving in the form of a functional ‘trade-off’ between aerial and sub-aqueous flight (Storer 1960; Olson and Hasegawa 1979; Bengston 1984; Livezey 1988; Habib 2010). This extreme specialization for wing-propelled diving results in characteristics that are shared not only among flightless alcids, but also with penguins and plotopterids. It was the outward resemblance of Spheniscidae to the familiar Great Auk *Pinguinus impennis* of the northern Atlantic Ocean that prompted sailors who first encountered Spheniscidae in the southern hemisphere to call them penguins (Olson and Lund 2007). Osteological characteristics shared between flightless alcids and penguins include decrease in range of motion and shortening of the distal wing elements in comparison with volant alcids (Raikow et al. 1988; Fig. 16), distal elongation of metacarpal one (Fig. 17), arced or curved wing elements (Fig. 1), an increase in the size of the tricipital crests of the distal humerus (Fig. 1), and a deeply grooved ventral margin of the ventral tubercle (Fig. 1). Mancallinae share additional convergent characteristics with Spheniscidae such as dorsoventral expansion of the omal extremity of the furcula, and deeply incised lateral sternal notches (Fig. 9). Although the functional significance of these modifications is not precisely known, the demands of wing-propelled pursuit diving for fish involving powered up-strokes and down-strokes likely played a role in the evolution of the convergent morphological characteristics shared by flightless alcids and penguins.

One characteristic that is unique to Mancallinae among all known flightless birds, is the shorter length of the ulna compared with that of the carpometacarpus (180:1). In most birds these proportions are opposite of that observed in Mancallinae, with the ulna being longer than the carpometacarpus. Three associated Mancallinae specimens (LACM 107028; SDSNH 77966), including the holotype specimen of *Mancalla cedrosensis* (LACM 15373) display this characteristic. Statistical analysis of osteological proportions of flightless alcids quantified the dorsoventral compression of wing elements and shortening of distal wing elements, but surprisingly, Livezey (1988) did not mention the unique relationship between the lengths of the ulna and carpometacarpus. A survey of the proportions of distal wing elements among extinct and extant birds was conducted to assess the distribution of this character state. The only other birds that are known to share this characteristic are several species of hummingbirds (e.g., *Phaethornis pretrei*; see Mayr 2004, Table 1). The precise functional significance of having a longer carpometacarpus than ulna would require detailed functional morphological study, but given the extreme pectoral specialization of both Mancallinae and Trochilidae, and the need of both of these taxa to produce thrust on both up-strokes and down-strokes, it seems reasonable to postulate that the increased dependence on thrust generated from primary feathers attached to the carpometacarpus (Chai 1997) may play a role in this osteological modification. Although the primaries of Mancallinae would likey have been much shorter than those of Trochilidae, water is a considerably more dense flight medium with different functional requirements than those for aerial flight (Habib 2010). Interestingly, this characteristic is not known in any extinct or extant penguin (J. Clarke, pers. com.).

The relatively large size of *Pinguinus* and some Mancallinae as compared to other alcids (Livezey 1988) may be linked with flightlessness, because the decreased buoyancy of large body size confers an advantage to piscivorous predators (Sparks and Soper 1987). Additionally, because these diving birds likely spent the majority of their time in the water (i.e., flightless, and came ashore only to breed), the thermal constraints imposed on them are decreased by large body size (Furness and Burger 1988). Furthermore, because Mancallinae were flightless, weight constraints related to maintaining the ability for aerial flight no longer restricted increases in body mass (Simpson 1946, Ksepka et al. 2006). Estimates of body mass in Mancallinae (excluding *Miomancalla howardi*) range from 1 kg in the smallest species (i.e., *Mancalla californiensis*) to 4kg in larger species (i.e., *Mancalla lucasi*; Livezey 1988). Although smaller than the 5kg mass estimated for *Pinguinus*, the estimated body mass of Mancallinae is greater than volant extant alcids (Livezey 1988). *Miomancalla howardi* is the largest known Mancallinae, and given the increased shortening and dorsoventral compression of wing elements of Mancallinae as compared to *Pinguinus*, it may have approached the mass of *Pinguinus*. Several Pliocene species of *Alca* are known to have exceeded the size of extant *Alca torda* (Olson and Rasmussen 2001; Smith and Clarke *in review*), and estimates based on fossils from Belgium indicate that at least one Pliocene Atlantic species, *Alca stewarti* Martin et al., 2001, was approaching the wing-loading threshold for flapping-flight (Martin et al. 2001; Dyke and Walker 2005). This apparent trend towards increased size in two separate alcid lineages, known from separate ocean basins during the Miocene and Pliocene is in stark contrast to the smaller body size of most extant alcids. The largest extant alcids are the Murres (*Uria aalge* and *Uria lomvia*), with an average body mass of 800-1000g, but the most speciose clade of extant alcids, the auklets *Aethia* and *Ptychoramphus*, are among the smallest of extant alcids with a body mass of 85-297g (del Hoyo et al. 1996). Additionally, the Mancallinae lineage and the *Alca* + *Pinguinus* lineage are considered the dominant seabirds in their respective oceans during the Pliocene (Olson 1985; Olson and Rasmussen 2001). This temporal disparity in size suggests that the conditions that led to radiations of large alcids in the Pacific and Atlantic Oceans are no longer in place, and that small to moderate size may have played a role in differential survival of alcid species since the Pliocene. However, the largest known alcid, the Great Auk, was not driven to extinction by competition from smaller species or lack of ability to adapt to a changing environment, but rather was exterminated through human exploitation (Bengston 1984; Fuller 1999).

Body size in extant alcids has been correlated with dive depth and feeding ecology (Piat and Nettleship 1985; Prince and Harris 1988; Watanuki and Burger 1999), andlarger body size in extant alcids is associated with piscivory (Bradstreet and Brown 1985). Foraging ranges, dive depths, and prey selection are similar in extant alcids and penguins (Prince and Harris 1988). Little is known about the feeding strategies of *Pinguinus* (Olson 1977), and there is no direct evidence of feeding strategies in Mancallinae; however, the large size of many Mancallinae and morphological comparisons with extant piscivorous alcids suggest that Mancallinae were specialized for piscivory. For example, the terete bill of *Mancalla* (e.g., LACM 103940) may be evidence of piscivory, because this characteristic in alcids has been linked with that feeding strategy **(**Bédard 1969).

### Geological and phylogenetic context for Pan-Alcidae

The oldest unequivocal fossil alcid (GCVP 5690) is from Late Eocene deposits of the Hardie Mine, Gordon, Georgia, USA (Chandler and Parmley 2002). Likely because of the incompleteness of the specimen, phylogenetic results (not shown) place this specimen at the base of Alcinae in an unresolved polytomy with other Alcinae clades. However, this placement is based upon a single shared character (equal width of the tricipital sulci) and the possibility that characteristics shared with Alcinae are pleisiomorphic for Alcidae should be considered. Accordingly, this fossil is considered Alcidae incertae sedis, rather than Alcinae insertae sedis. The presence of alcids in Late Eocene (Chandler and Parmley 2002) is congruent with divergence estimates placing the origin of Alcidae in the Paleocene (Baker et al. 2007; Pereira and Baker 2008). Although, as pointed out by Wijnker and Olson (2009), those divergence estimates suffer from serious flaws with respect to the taxonomic status and ages assigned to fossils used as calibrations.

The taxonomic status of all but one earlier (i.e., Mesozoic, Paleocene, and Early-mid Eocene) fossil referred to Charadriiformes (Olson and Paris 1987; Harrison and Walker 1977) consists of unassociated, undiagnosable fragments (Hope 2002; Mayr 2005, 2009). The earliest known definitive charadriiform fossil is a humerus that is tentatively referred to the Charadri (Hou and Ericson 2002). Although no radiometric-based date is known for this fossil, the age of *Jiliniornis huadianensis* Hou & Ericson, 2002 (IVPP V.8323) is estimated to be Middle Eocene based on biostratigraphic correlation (Hou and Ericson 2002). A minimum age of divergence between Alcidae and other charadriiforms in the Eocene suggests that the charadriiform fossil record is quite incomplete (i.e., extensive ghost lineages inferred based upon the fossil record).

The fossil record of Mancallinae ranges in age from Middle Miocene through Late Pleistocene (i.e., Turtonian-Calabrian; Becker 1987). The oldest record of Mancallinae may be the holotype specimen of *Miomancalla wetmorei* (LACM 42653) from the Mid-Late Miocene Monterey Formation exposed in Laguna Niguel, California; although, the precise stratigraphic position of the holotype locality is unknown. Deposition of the Monterey Fm. spans ~10ma from 17.9-7.4 Ma (i.e., Turtonian; DePaolo and Finger 1991). The holotype specimen is from the upper part of the formation (Domning and Deméré 1984), and would therefore be ~12-7.4 Ma. *Miomancalla howardi* is known from the Late Miocene San Mateo Formation, which ranges in age from 8.7-4.9 Ma (Zanclean-Messinian; Deméré and Berta 2005). *Miomancalla* is replaced in Pliocene sediments by *Mancalla*, with four species known from the Capistrano, San Diego, San Mateo, Niguel, Almejas, and Purisima Formations. The San Mateo Fm. records the highest diversity of Mancallinae, with *Miomancalla howardi* found in the lower unit, and *Mancalla cedrosensis*, *Mancalla lucasi*, and *Mancalla vegrandis* from the upper unit. The Capistrano Fm., which may be correlative with the San Mateo Fm. (Deméré and Berta 2005), has produced remains of *Miomancalla howardi* from the lower unit and *Mancalla californiensis* from the upper unit. The most geographically widespread and chronologically long-lived species (~5.0 Ma - 470 ka) is *Mancalla lucasi*, known from the Pliocene San Mateo, San Diego, and Niguel formations, and also from the Pleistocene Hookton Formation (Howard 1970; Kohl 1974; Domning and Deméré 1984).

Just as coldwater upwelling is linked to biological productivity in modern seabird communities (Hyrenbach and Viet 2003; Briggs et al. 1987) the Miocene appearance of *Miocepphus* in the Atlantic Ocean and *Miomancalla* in the Pacific Ocean coincides with the formation of permanent Antarctic icecaps and shallowing of the Central American Seaway (CAS) that resulted in steeper latitudinal thermal gradients. This resulted in intensified gyral circulation of surface waters, and strengthened coastal and trade winds that promote upwelling (Ford and Golonka 2003). The Early Pliocene (~5 - 3.5 Ma) was a time of relative climate stability and high sea level that coincides with the appearance of speciose alcid lineages in the Atlantic and Pacific Oceans (Warheit 1992). High Mancallinae diversity in the Pacific Ocean, and high *Alca* diversity in the Atlantic Ocean (Olson and Rasmussen 2001; Smith and Clarke *in review*) coincides with documented cooling during the Late Miocene and Early Pliocene (~14-3.6 Ma), and establishment of the California current system in the Pacific (Zachos et al. 2008; Lariviere et al. 2009).Although the geology of eastern Pacific marine units is more complex than that of coeval geologic formations from the passive Atlantic margin, sea-level fluctuation records indicate that the same Early Pliocene cycles of transgression and regression are recorded on western Atlantic and eastern Pacific coasts (Haq et al. 1988). The Middle Pliocene (~3.5–3.0 Ma) was characterized by continued global cooling, continued shallowing of the CAS, and the beginning of northern hemisphere glaciation cycles which led to increased coldwater upwelling in the Pacific (Bartoli et al. 2005; Lawrence et al. 2006). The emergence of the Panamanian Isthmus and the final closure of the CAS at ~2.7 Ma resulted in increased northern hemisphere glaciation, which is associated with a severe drop in sea-level (~45m) and the establishment of the modern profile of the California ocean-current system on which Pacific alcids rely today (Hyrenbach and Viet 2003; Bartoli et al. 2005). The microfaunal record documents a southward shift in Atlantic and Pacific cold-adapted foraminiferal faunal regimes (Bartoli et al. 2005), separation of Pacific and Caribbean cocolith assemblages at 2.74 Ma in response to final closure of the Isthmus, and an increase in thermohaline circulation as a result of separation of the Atlantic and Pacific Ocean basins. By ~2.5 Ma the modern climate regime was in place, involving small (i.e., meter scale) fluctuations in sea level associated with Late Pliocene and Pleistocene glacial cycles (Bartoli et al. 2005). The apparent response of seabirds to these climate-related changes in the environment was a significant decrease in diversity (Warheit 1992; Olson and Rasmussen 2001; Dyke and Walker 2005), because only a single species of *Alca* survives today in the Atlantic,and only a single specimen of *Mancalla* is known from the Pleistocene (Howard 1970; Kohl 1974). Confirmation of causal links between these climatic shifts and decreased seabird diversity will require more intense sampling of Late Pliocene and Pleistocene seabird fossils and evaluation of other proposed factors such as competition for nesting grounds with pinnipeds (Warheit and Lindberg 1988).

Known diversity of extinct Atlantic alcids now approaches that of extinct Pacificalcids (~16–19 species ranging from Miocene-Pleistocene age; Smith and Clarke *in review*). The differential extinction of Atlantic alcids, compared with that of Pacific lineages, may be linked to climatic changes that effected the Atlantic and Pacific Oceans in different ways. The alcid Pacific Ocean origin hypothesis is based primarily on higher extant diversity in the Pacific Ocean; however, higher extant diversity in the Pacific is not evidence of origination area, and the two oldest known alcid fossils are both from Atlantic deposits (Wetmore 1940; Chandler and Parmley 2002; Wijnker and Olson 2009). Although the lack of older fossils from the Pacific may simply reflect a gap in the fossil record, hypotheses concerning Pacific ancestral origination of alcids based upon proposed greater extant Pacific species diversity should accordingly be re-evaluated. However, the basal position of Mancallinae and their restriction to the Pacific basin may be viewed as support for the Pacific origin hypothesis for Pan-Alcidae (Storer 1952; Kozlova 1957; Olson 1985; Konyukhov 2002; Pereira and Baker 2008).

Regardless of the ancestral area of the clade (i.e., Atlantic or Pacific), hypotheses regarding the spread of alcids from one ocean basin to another include dispersal by ice-free northern passage through the Bering Strait and Arctic Ocean, and southern dispersal across the submerged Isthmus of Panama (Olson 1985; Konyukhov 2002; Pereira and Baker 2008). These hypotheses are based upon the assumption of dispersal across water, and the first occurrence datum (FAD) for alcid clades, which until the discovery of an auk from the Eocene of Georgia, USA (Chandler and Parmley 2002), included Miocene examples of Mancallinae (Howard 1976), *Cepphus* (Howard 1982), and *Uria* (Howard 1981) from Pacific deposits, and *Miocepphus* (Wetmore 1940) and *Alca* (Wijnker and Olson 2009) from Atlantic deposits. The ornithological literature is replete with records of occurrences of alcids hundreds or even thousands of miles from their normal ranges (see Konyukhov 2002) and records of alcid ‘wrecks’, sometimes composed of thousands of individuals, that were blown many kilometers inland from the sea by storms (Fisher and Lockley 1954; Stewart 2002a). Given the expanse of geologic time being considered (Paleocene-Recent), the possibility that such events may have led to the dispersal of populations from one ocean basin to another ocean basin must be considered.

As suggested by Bédard (1985), the presence of Atlantic alcids in the Eocene (Chandler and Parmley 2002) confirms that the cold adapted lifestyle of some alcids (e.g., *Uria*) evolved from ancestors that were adapted to warmer (i.e., Eocene) climates. The development of basically modern ocean circulation patterns was not achieved until ~24–20 Ma when opening of Drake Passage initiated dramatic cooling of Antarctica and formation of a strong Antarctic current that resulted in a switch from high productivity in equatorial regions, to more northern coastal regions (Lear et al. 2000; Ford and Golonka 2003; Liu et al. 2009). Although the southern location of the earliest alcid fossil locality (Georgia, USA) cannot necessarily be interpreted as support for a southern route of dispersal, warm-adapted alcids in the Eocene likely were not restricted to a northern dispersal route.

## Conclusions

Rigorous taxonomic evaluation of alcid fossil material resulted in a more refined picture of diversity within Mancallinae, and facilitated phylogenetic analysis of species-level relationships within the clade. The combined analysis and total evidence approaches adopted herein resulted in a well-resolved and strongly supported hypothesis of the position of Mancallinae with respect to other Charadriiformes, and the inter-relationships of Mancallinae species. The phylogenetic position of Mancallinae as the sister taxon to all other Alcidae (i.e., crown clade Alcidae) suggests extensive ghost lineages in Pan-Alcidae, provides further evidence that the charadriiform fossil record is quite incomplete, and demonstrates that flight was lost independently in at least two lineages of alcids. The stem-lineage position of Mancallinae recovered in this analysis is consistent with previous phylogenetic placement of this clade (Chandler 1990a), but contrasts with previous hypotheses of close relationship between Mancallinae and Alcinae (Olson 1985). Although extremely derived morphologically as a result of modifications related to flightlessness, Mancallinae do possess a unique suite of characters, some of which are otherwise found exclusively in Alcinae or Fraterculinae, and some of which are otherwise known only from non-alcid charadriiforms. Although it would not affect the number of inferred origins of flightlessness in Alcidae, the placement of Mancallinae at the base of Alcinae, or at the base of Fraterculinae, would only require an additional 2 steps of tree length (manually calculated in MacClade; Maddison and Maddison 2005), and thus the position of Mancallinae recovered here may be sensitive to the inclusion of additional fossil taxa with morphologies representing ancestral states for Pan-Alcidae. The hypothesized split between the lineages leading to Mancallinae and crown clade Alcidae raises questions about the evolution of flightlessness in charadriiforms, and the biological factors that may have led to the split between Alcidae and their proposed sister taxon, Stercorariidae.

*Miomancalla howardi* is placed as the sister taxon of *Miomancalla wetmorei*, and is the largest known species of Mancallinae. The large size and resemblance of the bill of *Miomancalla howardi* to that of the Great Auk *Pinguinus impennis* provides an example of within-lineage convergence between two species separated by time and geography. The independent acquisition of morphological characteristics in both lineages of flightless alcids (i.e., Mancallinae and *Pinguinus*), and the similarity of these modifications to those of penguins and plotopterids, strongly suggests correlation between these morphologies and mode of locomotion. The study of convergence within Alcidae may provide insights about the evolution of flightlessness in penguins, in which there are no known volant species.

Similarly diverse lineages of alcids inhabited the eastern and western coasts of North America during the Miocene and Pliocene. Approximately coeval Early Pliocene deposits in California and North Carolina record the replacement of *Miocepphus* by *Alca* in the Pliocene of the Atlantic, and the replacement of *Miomancalla* by *Mancalla* in the Pliocene of the Pacific. Global-scale environmental perturbations such as increased cooling following the MMCO, may have contributed to similar scenarios involving species turnover in Pan-Alcidae in both ocean basins.

## Supplementary Material

XML Treatment for Mancalla

XML Treatment for Mancalla lucasi

XML Treatment for Mancalla vegrandis

XML Treatment for Miomancalla

XML Treatment for Miomancalla howardi

## Appendix 1. Review of the Mancallinae fossil record

Owing to the recognition of several Mancallinae species based upon non-diagnostic material, the systematics of Mancallinae required extensive revision. The following review of the Mancallinae fossil record is presented to clarify the systematic position of previously named species and referred fossil material, and to justify the exclusion of some previously named species from the phylogenetic analysis.

Although more than 100,000 avian fossils are now known from sediments in California (Miller 1946; Brodkorb 1967; Olson, 1985), the first avian fossil from this state was not reported until 1901 when F. A. Lucas described a nearly complete left humerus from what were thought to be Late Miocene sediments of Los Angeles. That specimen (USNM 4976; Fig. 2) represented the first of approximately 4000 fossils that are now referred to the flightless alcid taxon *Mancalla* (Smith, pers. obs). *Mancalla californiensis* Lucas 1901 was the first of seven flightless alcid species recognized between 1901 and 1981 (Table 1).

The second report of *Mancalla* remains (humerus; catalog # uncertain) came from the Early Pliocene San Diego Formation exposed in San Diego, California (Miller 1933). The Early Pliocene age of that specimen was congruent with the revised age estimate for the holotype locality of *Mancalla californiensis* in Los Angeles (Arnold 1906). An additional specimen, a complete right femur (UCMP 33409) from the San Diego Fm., was reported by Miller in 1937. Based on characteristics of that specimen Miller (1937) considered it a Pliocene example of a puffin (i.e., ‘*Lunda*’, *Fratercula*, and *Cerorhinca*), and designated the specimen as the holotype of a new taxon, *Pliolunda diegense* Miller, 1937. Additional *Mancalla* remains (LM 2218) were reported by Miller (1946), who discussed the possibility that *Pliolunda* was a synonym of *Mancalla*, and erected the Family Mancallidae, separating *Mancalla* from Alcidae. The rank of Mancallidae later became subfamily Mancallinae (sensu Brodkorb 1967), systematically reuniting *Mancalla* and *Praemancalla* with other Alcidae.

Mounting evidence that more than one species of *Mancalla* was present during the Early Pliocene came from Howard in 1949. At that time approximately 118 specimens representing *Mancalla* were known, including two size classes of carpometacarpi from localities in Los Angeles, San Diego, and Corona del Mar, California. Although no associated remains were known, carpometacarpi were referred to *Mancalla* based upon characters such as an elongated first metacarpal, a morphology considered convergent with that of penguins (i.e., Spheniscidae) by Howard (1949). Humeri of *Mancalla* were well known, and also display characteristics related to extreme specialization for wing-propelled diving considered by Miller (1946) to be convergent with those of penguins, and prompting the referral of carpometacarpi exhibiting ‘penguin-like’ features.

The growing number of specimens from the San Diego Fm. prompted a review known remains of *Mancalla* (Miller and Howard 1949), which resulted in the recognition of *Pliolunda* as a junior synonym of *Mancalla*. However, additional remains other than humeri were referred solely on the basis of size, provenience, and osteological characteristics correlated by those authors with flightlessness. No associated *Mancalla* remains were known at the time that would allow for referral of femora to *Mancalla californiensis*, nor to facilitate comparisons between *Mancalla californiensis* and the holotype specimen of *Mancalla diegense*. The species name *Mancalla diegense* was emended to *Mancalla diegensis* by Olson (1981) to reflect correct latinization of the place name San Diego. Although my recent re-examination of *Mancalla* material in the collections of UCMP, LACM, and SDSNH identified several associated specimens within the size range of *Mancalla diegensis* as reported by Howard (1970), and that correspond with characters described for that taxon by Howard (1970), no associated specimens referable to *Mancalla californiensis* that preserved femora were identified. The holotype femur of *Mancalla diegensis* is, therefore, not presently comparable to *Mancalla californiensis*. Furthermore, my survey of the femora of all known alcid species revealed that the morphology of the femur is remarkably conserved across alcid taxa, potentially explaining Miller’s (1937) original proposal, that UCMP 33409 represented an extinct species of puffin. No characteristics were identified that would allow for confident referral of isolated femora to *Mancalla*, and *Mancalla diegensis* is, therefore, considered Pan-Alcidaeincertae sedis.

In 1966 Howard described a new Mancallinae taxon from the Late Miocene based upon isolated elements including a distal humerus, carpometacarpi, a partial coracoid, the proximal end of a scapula, and the articular portion of a mandible. *Praemancalla lagunenesis* Howard, 1966 was considered by that author to be less specialized with respect to features associated with loss of aerial flight, and the possibility that *Praemancalla* might represent a less derived ancestor of *Mancalla* was proposed. All elements referred to *Praemancalla lagunenesis* were isolated, so only the holotype distal humerus (LACM 15288) can be compared with previously recognized taxa to evaluate the taxonomic validity of this species. The holotype specimen of *Praemancalla lagunensis* is weathered smooth, obscuring many fine morphological details. Although LACM 15288 is referable to Mancallinae based upon the rounded anterior surface of the ventral condyle (153:0), all of the characteristics that Howard (1966) proposed as diagnostic for this species may be an artifact of weathering, or also are found in *Mancalla*. *Praemancalla lagunensis* is, therefore, considered Mancallinaeincertae sedis.

Another species of alcid with characteristics interpreted as “progressing towards flightlessness” (Howard 1968: 19), was described by Howard in 1968 from presumed Miocene sediments of Laguna Hills, California. *Alcodes ulnulus* Howard, 1968 was described based upon isolated elements including a complete left ulna, additional ulnar fragments, and a partial carpometacarpus (Howard 1968). Additional material representing this taxon was recovered from the Middle Miocene Topanga Formation along Oso Creek in Orange County, California (Howard and Barnes 1987), confirming the Miocene age of this species. Ulnae of *Alcodes* are differentiated from those of *Mancalla* by their more gracile and rounded shafts, and projection of the olecranon farther posteriorly. Although associated humeri and ulnae of *Mancalla* (e.g., holotype specimen of *Mancalla cedrosensis* LACM 15373)demonstrate that *Alcodes* is distinct from *Mancalla*, the lack of associated *“Praemancalla”* specimens with ulnae raisesthe possibility that *Alcodes* is congeneric with *“Praemancalla”*. Until additional material is recovered that would allow comparison with other recognized alcid taxa, the systematic affinities of *Alcodes* in Pan-Alcidae remain uncertain, and *Alcodes* is, therefore, considered Pan-Alcidae incertae sedis.

Although the reviewby Howard (1970) expanded the known geographic range of *Mancalla*, and greatly increased knowledge of character-and size-related differences in the taxon, the description of *Mancalla milleri* Howard, 1970 based upon isolated material further complicated the taxonomy of the clade. Comparisons between the holotype femur of *Mancalla diegensis* and the holotype femur of *Mancalla milleri* (LACM 2185) provide limited information because neither of those elements is directly comparable to the isolated holotype humerus of *Mancalla californiensis*. Additionally, my recent re-examination of the ~4000 fossils referred to *Mancalla* indicates that femoral characters cited by Howard (1970) are more variable within proposed size classes of *Mancalla* than previously recognized (Smith pers. obs.). Furthermore, as stated above, femoral morphology is remarkably conserved in Alcidae. Although characteristics of humeri indicate that multiple species of *Mancalla* are represented by *Mancalla* material from the San Diego Fm., the species to which the holotype femora of *Mancalla diegensis* and *Mancalla milleri* belong will likely never be determined. *Mancalla milleri* is, therefore, considered Pan-Alcidaeincertae sedis.

*Mancalla cedrosensis* Howard, 1971 was the first species of *Mancalla* described from associated remains, and also the first that was directly comparable to *Mancalla californiensis* (Howard, 1971). The holotype specimen (LACM 15373; Fig. 2) and additional referred specimens were recovered from Early Pliocene deposits on Cedros Island off the coast of Baja California, Mexico (Howard 1971). The associated remains of *Mancalla cedrosensis* provided the first reliable assessment of inter-element osteological proportions for *Mancalla*,proportions that supported earlier size-based estimates of diversity among material from the San Diego Fm. proposed by Howard (1970).

*Praemancalla wetmorei* Howard, 1976 was described based upon a nearly complete humerus (LACM 42653; Fig. 2) from Late Miocene sediments in Laguna Niguel, California. Several features distinguish this species from other *Mancalla* (see diagnoses below). An associated specimen (LACM 107028) was tentatively referred to *Praemancalla wetmorei* by Howard (1982) on the basis of overall resemblance between the ulna of that specimen and the paratype ulna of *Praemancalla wetmorei*. Because the paratype ulna (LACM 32429) is not associated with the holotype humerus, and was referred only on the basis of its occurrence within the same deposit, the affinities of that specimen remain uncertain. Likewise, the affinities of additional non-humeral material (e.g., LACM 53907, 37637, 52216) referred to *Praemancalla wetmorei* by Howard (1976) are uncertain, because those specimens are not comparable to the holotype, and therefore not referable to species at this time. As stated above, the name-bearing specimen of *Praemancalla* (i.e., *Praemancalla lagunensis* Howard, 1966) is Mancallinae incertae sedis. Based upon phylogenetic results and apomorphies shared with *Miomancalla howardi* sp. n., *Praemancalla wetmorei* is referred to *Miomancalla*, and becomes *Miomancalla wetmorei* (Howard 1976).

*Mancalla emlongi* was described based upon a complete ulna from Early Pliocene San Diego Fm. sediments in San Diego, California (Olson 1981). In the original description, comparisons were made between the holotype specimen of *Mancalla emlongi* (USNM 243765) and ulnae referred to *Mancalla californiensis*, *Mancalla diegensis*, *Mancalla milleri*, and *Mancalla cedrosensis*. As stated above, size and provenience alone do not in my opinion constitute strong evidence that material is referable to a taxon previously known from a particular locality or geologic formation. *Mancalla diegensis* and *Mancalla milleri* are Alcidaeincertae sedis, and there are no known associated specimens that would allow for referral of ulnae to *Mancalla californiensis*. Although the holotype ulna of *Mancalla emlongi* can be differentiated from ulnae of *Mancalla cedrosensis*, the possibility exists that *Mancalla emlongi* is synonymous with another species ofMancallinae (e.g., *Mancalla californiensis*). *Mancalla emlongi* is, therefore, considered Mancallinaeincertae sedis.

Additional material from the San Diego Fm. including a well-preserved skull and mandible (SDSNH 25236; Fig. 3) was tentatively referred to *Mancalla emlongi* by Chandler (1990b) on the basis of size. SDSNH 25236 and an additional skull (SDSNH 23753) are referable to Alcidae based upon the strongly protruding cerebellar prominence (35:0), and deeply incised temporal (31:1) and nasal fossae (20:1). Although no cranial apomorphies of Mancallinae have thus far been identified, the cranium of Mancallinae can be differentiated from the skulls of all other known Alcidae: differentiated from Fraterculinae (*Aethia*, *Ptychoramphus*, *Cerorhinca*, and *Fratercula*) by the dorsal position and extension of the temporal fossae; differentiated from *Brachyramphus*, *Synthliboramphus*, *Alle*, *Miocepphus*, *Alca*, *Pinguinus*, *Cerorhinca* and *Aethia* by the lack of supraoccipital foramina; differentiated from *Cepphus* by protrusion of the cerebellar prominence farther posteriorly (condition resembles that in *Uria*), and deeper interhemispherical furrow along midline of skull; differentiated from *Uria* by depth of nasal fossae (deeper, distinctly bordered posteriorly, and laterally incised in *Uria*). Although these specimens cannot be referred to speciesat this time, two associated specimens comprising associated cranial and postcranial material (LACM 103940 *Mancalla* sp. and SDSNH 68312 *Miomancalla howardi* sp. n.) allow for comparison of SDSNH 25236 with known cranial morphology of Mancallinae. All three of the aforementioned specimens possess two small caudal mandibular fenestrae (46:1; Fig. 3D), a characteristic known only in the Fraterculini (i.e., *Fratercula* and *Cerorhinca*) among crown Alcidae, and also in the proposed sister taxon of Pan-Alcidae, the Stercorariidae. SDSNH 25236 is differentiated from Fraterculini by the lack of dorsoventral expansion of the premaxilla and mandible (2:0), and by the less acute angle formed between the jugal and the proximo-ventrally descending bar of the nasal (6:0). SDSNH 25236 is consistent in size and morphological characteristics with the skull and mandible of LACM 103940, which is the only known *Mancalla* specimen with both cranial and postcranial elements preserved. Additionally, SDSNH 25236 lacks the dorsoventrally expanded mandible of *Miomancalla howardi*, suggesting systematic placement within *Mancalla*.

Although *Mancalla* remains were reported from Pleistocenesediments inShiriya, Japan (Hasegawa et al. 1988), that material was never described, figured, nor systematically evaluated. My recent reexamination of the material confirms its referral to Mancallinae. The presence of *Mancalla* in Japan provides a considerable range extension, and based upon the age of the material, also confirms that *Mancalla* survived into the Pleistocenein the eastern and western Pacific Ocean (Howard 1970; Kohl 1974).

## Appendix 2. Comparative material

*Aethia cristatella* Crested Auklet:

Skins: NCSM 6564, 6565, 6567, 16419, 17749.

Skeletons: NCSM 17749; USNM 223707, 488675, 498282, 561934, 61094.

Eggs: USNM 32126, 32128, 32131, 33167.

*Aethia psittacula* Parakeet Auklet:

Skins: NCSM 16423, 16424, 18387; USNM 89143, 493708.

Skeletons: NCSM 14147, 14804, 18387, 18514, 20177; NSM PO 355; USNM 12640, 226451, 610513, 610514, 610937.

Eggs: USNM 42123, 42124, 42125, 42126.

Dissection: NCSM 20881.

*Aethia pusilla* Least Auklet:

Skins: NCSM 17735, 17736, 17751, 17797.

Skeletons: NCSM 17734, 17736, 17737; USNM 224009, 224010, 498285; NSM PO 356, 357.

Eggs: USNM 16725, 18052, 25103, 33886.

*Aethia pygmaea* Whiskered Auklet:

Skins: NCSM 13159; USNM 4163, 67399, 85617, 92971, 110194.

Skeletons: USNM 344544; UMMZ 204592, 224279, 224882, 224883.

Eggs: Scored from Baicich and Harrison (1997).

*Alca torda* Razorbill:

Skins: NCSM 298, 299, 2236, 4455, 18760, 20015.

Skeletons: NCSM 20058, 20502; USNM 18062, 347946, 501644, 502378, 502382, 502387, 502388, 502389, 502549, 555666, 555668.

Eggs: NCSM 13447, 13448; USNM 18476, 21571, 23259.

*Alle alle* Dovekie:

Skins: NCSM 301, 302, 303, 304, 20111, 20630, 40060,.

Skeletons: NCSM 18374; USNM 344740, 344748, 499471, 560929.

Eggs: USNM 2634, 18490, 18491, 19053.

Dissection: NCSM 21042.

*Anous tenuirostris* Lesser Noddy:

Skins: USNM 486718, 486723, 486725, 486728.

Skeletons: USNM 488400, 622578.

*Bartramia longicauda* Upland Sandpiper:

Skins: NCSM 825, 826, 827, 828, 3093.

Skeletons: USNM 227823, 347894, 610844, 610845, 501160,.

*Brachyramphus brevirostris* Kittlitz’s Murrelet:

Skins: NCSM 35213; USNM 286494, 333257, 589672.

Skeletons: USNM 288086, 288087.

Eggs: USNM 47733.

*Brachyramphus marmoratus* Marbled Murrelet:

Skins: NCSM 5669, 5670, 18144, 18145, 18146, 18148.

Skeletons: NCSM 18143, 18144, 18145, 18146, 18147, 18148, 18149; NSM PO 354, 358, 551.

Eggs: USNM 21545, 28473, 40125, 417778.

*Brachyramphus perdix* Long-billed Murrelet:

Skins: USNM 108952, 109985, 120704, 200411, 200412.

Skeletons: USNM 582506, 599498.

*Charadrius vociferus* Killdeer:

Skins: NCSM 791, 792, 17610, 18671, 19284.

Skeletons: NCSM 18305, 21905; USNM 61432, 492870, 553817, 622526.

Eggs: NCSM 13382, 13383, 13384, 13385, 13386, 13387.

*Charadrius wilsonia* Wilson’s Plover:

Skins: USNM 220535, 338822, 338823, 524172.

Skeletons: NCSM 5818; USNM 1250, 556652, 610801.

Eggs: NCSM 13388, 13389; USNM 43430, 43431, 43432.

*Cepphus carbo* Spectacled Guillemot:

Skins: USNM 40637, 102199, 406348, 424970.

Skeletons: USNM 347755, 347756, 347757.

*Cepphus columba* Pigeon Guillemot:

Skins: NCSM 16153, 16155,16414, 16438, 16439.

Skeletons: NCSM 18094, 18095, 18096, 18097.

Eggs: NCSM 13449; USNM 19063, 21546, 27059.

Dissection: NCSM 21075.

*Cepphus grylle* Black Guillemot:

Skins: NCSM 6830; USNM 331585, 393556, 394525.

Skeletons: USNM 344759, 344760, 347265, 612213, 612214.

Eggs: NCSM 7435, 13450, 13451; USNM 2578, 18494.

*Cerorhinca monocerata* Rhinoceros Puffin:

Skins: NCSM 8064, 10628, 16420, 16421, 16430.

Skeletons: NSM PO 189; USNM 557613, 557614, 561468, 620641, 620643.

Eggs: USNM 12866, 24634, 27632, 27633.

*Chlidonias leucopterus* White-winged Black Tern:

Skins: NCSM 11351, 11352, 11358, 11470, 11471.

Skeletons: USNM 43173, 290154, 430844, 431172, 488879.

*Creagrus furcatus* Swallow-tailed Gull:

Skins: NCSM 183825. USNM 115967, 115968, 131674, 543878, 543879.

Skeletons: USNM 18492, 19029, 498301.

*Cursorius temminckii* Temminck’s Courser:

Skins: USNM 448378, 520019, 545851, 545853, 545854.

Skeletons: 429182, 431709.

*Fratercula arctica* Atlantic Puffin:

Skins: NCSM 17824, 17825; USNM 589716, 627638.

Skeletons: USNM 18055, 18057, 18058, 224189, 621331.

Eggs: NCSM 13452; USNM 2637, 14977, 31034.

*Fratercula cirrhata* Tufted Puffin:

Skins: NCSM 16147, 16148, 16150, 16433, 18098.

Skeletons: NCSM 17823, 18099, 18100; USNM 19449, 488748.

Eggs: NCSM 13453, 13454; USNM 16335, 12861.

*Fratercula corniculata* Horned Puffin:

Skins: NCSM 7761, 10629, 18102; USNM 610504, 612200, 499957.

Skeletons: NCSM 17835, 18083, 18388; USNM 499961, 499964.

Eggs: USNM 16329, 19706, 22052, 25095, 29216.

Dissection: NCSM 21095.

*Glareola maldivarum* Oriental Pratincole:

Skins: NCSM 9756, 11059, 11060, 11061, 11062.

Skeletons: USNM 19580.

*Gygis alba* White Tern:

Skins: NCSM 7859, 7860, 8021, 18890, 18932.

Skeletons: NCSM 16895; USNM 498081, 498415, 559583, 621328.

*Hydrophasianus chiurgis* Pheasant-tailed Jacana:

Skins: NCSM 10609, 11018, 11019, 11473.

Skeletons: USNM 226034, 431604, 431609, 490560, 490566.

*Larosterna inca* Inca Tern:

Skins: USNM 15503, 15516, 212050, 212051, 371303.

Skeletons: USNM 292869, 430271, 430375, 430580, 430625, 631761.

*Larus argentatus* Herring Gull:

Skins: NCSM 17738, 21188, 21444, 21462, 21791.

Skeletons: NCSM 8624, 10116, 10211, 10251, 22218.

Eggs: NCSM 5934, 13395.

*Larus marinus* Great Black-backed Gull:

Skins: NCSM 7376, 7861, 7863, 7941, 7992.

Skeletons: NCSM 6590, 16190, 102451; USNM 491592, 502396.

Eggs: NCSM 5968; USNM 42295, 42296, 42297.

*Numenius minutus* Little Curlew:

Skins: NCSM 1907, 22227, 22228, 22229, 22230.

Skeletons: USNM 347648.

*Pagophila eburnea* Ivory Gull:

Skins: USNM 17766, 22217, 22221.

Skeletons: NCSM 17766; USNM 344734, 491595, 491596, 491597.

*Phaetusa simplex* Large-billed Tern:

Skins: NCSM 22224. USNM 316370, 326609, 349836, 512940.

Skeletons: USNM 345827, 345828.

*Pinguinus impennis* Great Auk:

Skins: USNM 57388 (eye and mouth color scored based on Smith, 1879).

Skeletons: USNM 346387 (composite), 557975 (composite), 623465 (composite) additional series of disarticulated USNM material from the expedition to Funk Island (Lucas, 1890).

Eggs: USNM 15141, 15144.

*Ptychoramphus aleuticus* Cassin’s Auklet:

Skins: NCSM 5666, 7222, 10624, 19137, 19140.

Skeletons: NCSM 18088; USNM 491305, 491845, 491846, 557607, 557609, 557611.

Eggs: NCSM 7901; USNM 2353,16635, 16636.

*Rhinoptilus chalcopterus* Bronze-winged Courser:

Skins: USNM 117798, 216168, 437251, 448203, 460101.

Skeletons: USNM 321515.

*Rhodostethia rosea* Ross’s Gull:

Skins: NCSM 22222, 22223. USNM 93346, 93356, 93357, 332306, 495943.

Skeletons: USNM 491606, 491607, 491608, 491609, 491611.

*Rissa tridactyla* Black-legged Kittiwake:

Skins: NCSM 18072, 18073, 18074, 18075, 18076.

Skeletons: NCSM 18123, 18124, 18125, 18126.

Eggs: NCSM 13403.

*Rynchops niger* Black Skimmer:

Skins: NCSM 281, 282, 287, 289, 20262.

Skeletons: NCSM 4228, 6280, 6281, 7790, 7791, 9725, 19048, 19063

Eggs: NCSM 13441, 13442, 13443, 13444, 13445.

*Stercorarius longicaudus* Long-tailed Skua:

Skins: NCSM 8385, 10269, 11725, 17144, 17801.

Skeletons: NCSM 10269, 17801; USNM 491643, 491951, 501243.

Eggs: USNM 7789, 11692, 11694, 11681, 11699.

*Stercorarius skua* Great skua:

Skins: NCSM 13193, 14891, 22191, 22192.

Skeletons: NCSM 11747; USNM 488294, 488295, 560938, 576076, 623300.

Eggs: USNM 14918, 24541, 34243, 42219, 42221, 46504.

*Sterna anaethetus* Bridled Tern:

Skins: NCSM 4066, 6037, 6039, 6042, 6086.

Skeletons: NCSM 10268, 17085, 19073; USNM 488397, 554970, 554972, 558277.

*Sterna maxima* Royal Tern:

Skins: NCSM 7213, 7294, 7614, 20050, 20668.

Skeletons: NCSM 1640, 10248, 16010, 17514.

Eggs: NCSM 2603, 2604, 5317, 13245, 13424, 13426.

*Sterna niloteca* Gull-billed Tern:

Skins: NCSM 242, 10461, 11469, 15044, 15046.

Skeletons: 10228, 15046, 17188, 289676, 501253, 610912.

Eggs: NCSM 8397, 8398, 8399, 9943, 9944.

*Sternula superciliaris* Yellow-billed Tern:

Skins: USNM 283, 682, 401268, 512943, 512944.

Skeletons: USNM 227482, 345825, 345826.

*Stiltia isabella* Australian Pratincole:

Skins: USNM 279023, 405699, 405698, 405700, 405701.

Skeletons: AMNH 9599.

*Synthliboramphus antiquus* Ancient Murrelet:

Skins: NCSM 16146, 17742, 18089, 19143.

Skeletons: NCSM 17742, 18089, 18090; NSM PO 351, 352, 427, 428, 564; USNM 488688, 561926.

Eggs: USNM 16618, 27130, 27131, 28369.

Dissection: NCSM 21074.

*Synthliboramphus craveri* Craveri’s Murrelet:

Skins: USNM 544024, 544034, 597160, 597163.

Skeletons: SDSNH 36390, 36391, 37767.

Eggs: USNM 42144, 46625, 46627, 46628.

*Synthliboramphus hypoleucus* Xantus’ Murrelet:

Skins: USNM 544886, 544887, 544889, 544893.

Skeletons: USNM 19387, 291879, 345427, 345428, 500652.

Eggs: USNM 28131, 31480, 46623, 46624.

*Synthliboramphus wumizusume* Japanese Murrelet:

Skins: USNM 15803, 85796, 111653, 114529, 466256.

Skeletons: NSM PO 10, 353, 359; UMMZ 152355, 152356, 152357, 152358, 152359, 152360.

*Tryngites subruficollis* Buff-breasted Sandpiper:

Skins: NCSM 7621, 21581, 22225, 22226.

Skeletons: USNM 7995, 227481, 227771, 492110.

*Uria aalge* Common Murre:

Skins: NCSM 8074, 11188, 18115, 18992, 20551.

Skeletons: NCSM 17822, 18116, 18117, 18118, 18234.

Eggs: NCSM 5935, 5936, 13455, 13456, 13457, 13773.

Dissection: NCSM 21070.

*Uria lomvia* Thick-billed Murre:

Skins: NCSM 6347, 16144, 16145, 17754, 17779.

Skeletons: NCSM 18114, 19414; USNM 344435, 561265.

Eggs: USNM 18502, 18504,18505, 19049, 24420.

*Xema sabini* Sabines Gull:

Skins: NCSM 3678, 16393, 16394, 17777, 17778.

Skeletons: NCSM 17778; USNM 499111, 533882, 533905, 557605, 557606.

## Appendix 3. Morphological character list

Osteology: Characters 1–223

Integument: Characters 224–255

Reproduction: Characters 256–266

Diet: Characters 267–268

Myology: Characters 269–292

Feather Microstructure: Characters 293–344

### Skull

**1**. **Premaxilla, anterior tip:** (0) decurved; (1) hooked. The anterior tip of the premaxilla is hooked ventrally in a raptorial fashion in some alcids (e.g., *Alca torda*). The anterior tip of the premaxilla in other alcids (e.g., *Brachyramphus marmoratus*) is decurved slightly ventrally but does not possess a hooked tip.

**2. Premaxilla, dorsal** **margin** (modified from Chandler 1990b, character 17)**:** (0) smooth; (1) anteriorly enlarged. While the premaxilla of most alcids is acute (e.g., *Uria aalge*) the premaxilla of some species (e.g., *Alca torda*) is laterally compressed, and enlarged anteriorly and dorsally.

**3. Maxilla, fenestra adjacent to junction of maxilla and palatine:** (0) absent; (1) present. The ventral surface of the distal end of the maxilla is fenestrated in some alcids (e.g., *Cerorhinca monocerata*). This characteristic is absent in many other alcids (e.g., *Cepphus grylle*). In life the fenestra is covered by a thin membrane. Because the fenestra does not serve as a passageway for muscle, tendon, or nerves, its purpose may be related to flexion or weight reduction.

**4. Nasal, anterior projection along the** **ventral surface of the** **premaxilla** (Chandler, 1990b, character 9): (0) contacting; (1) separated. The nasals converge beneath the premaxilla in some species (e.g., *Uria aalge*), while in other species (e.g., *Fratercula cirrhata*) the lateral nasal bars merge with the ventral premaxilla but remain separated.

**5. Nasal, maxillary spine on nasal bar** (Chandler 1990b, character 13): (0) short (i.e., <=1/2 the length of the nasal bar); (1) long (i.e., >1/2 nasal bar). A spine extends dorsally from the posterior maxilla and fuses with the nasal bar. In most alcids and other charadriiform species (e.g., *Uria lomvia*) the spine is only a short protuberance. In other species (e.g., *Brachyramphus brevirostris*) this spine extends more than halfway up the lateral nasal bar towards the frontal.

**6. Nasal bar, angle with respect to jugal:** (0) ~45 degrees; (1) ~60 degrees. The angle between the nasal and the jugal of most alcids (e.g., *Uria aalge*) is ~45 degrees, while in the auklets and puffins (e.g., *Fratercula cirrhata*) this angle is around ~60 degrees.

**7. Maxillopalatine strut** (Strauch 1985, character 1; Chandler 1990b, character 16): (0) absent; (1) present. The maxillopalatine strut is a small projection connecting the maxillopalatine process to the nasal bar. The maxillopalatine strut, which is found only in *Fratercula* among the Alcidae, does not appear to be homologous with those found in other charadriiforms (Lowe 1931; Bock 1958; Zusi and Jehl 1970; Strauch 1978). Its presence is considered derived (Strauch, 1985).

**8. Maxillopalatine process shape** (Strauch 1985, character 2; Chandler 1990b, character 3): (0) ventrolaterally concave; (1) flat. In most alcids (e.g., *Alca torda*) the maxillopalatine process is a rounded cuplike structure, while in *Aethia*, the maxillopalatine process is flat.

**9. Maxillopalatine process orientation** (Chu 1998, character 45): (0) dorsally tilted; (1) ventral, flat lying. While the maxillopalatine process of some alcids (e.g., *Aethia pygmaea*) are dorsoventrally oriented (i.e., horizontally lying), the maxillopalatine process of other alcids (e.g., *Alca torda*) are tilted such that their medial edges are dorsally elevated in relation to their lateral edges.

**10. Maxillopalatine process, anterior end, medial margin** **in ventral view** (Chandler 1990b, character 6): (0) rounded; (1) angled. In some species (e.g., *Alca torda*) the medial margin of the maxillopalatine process forms a gentle curve. This feature is anteriorly angled in other species of alcids (e.g., *Aethia pusilla*).

**11. Palatine,ventral extent of the medial margin of the ventral crest relative to the palatine shelf** (crista ventralis medialis, Baumel and Witmer 1993; Strauch 1985, character 3): (0) does not extend beyond ventral margin of palatine shelf; (1) extends beyond ventral margin of palatine shelf. The ventral end of the ventral crest of the palatine does not extend as far ventrally as the ventral edge of the palatine plate in most charadriiforms (e.g., *Alca torda*). In the auklets (e.g., *Aethia psittacula*) however, it extends beyond the end of the palatine shelf (Strauch 1985).

**12. Palatine, anterior** **margin of the** **medial palatal crest** (crista ventralis medialis; Baumel and Witmer 1993; modified from Chandler 1990b, character 3): (0) angular; (1) rounded. The anterior end of the medial palatal crest can be either rounded in shape (e.g., *Alca torda*) or angular (e.g., *Cepphus carbo*).

**13. Palatine, posterior** **margin of the medial palatal crest** (crista ventralis medialis; Baumel and Witmer 1993; modified from Chandler 1990b, character 3): (0) angular (1) rounded. The posterior end of the medial palatal crest can be either rounded in shape (e.g., *Cepphus carbo*) or angular (e.g., *Brachyramphus brevirostris*).

**14. Palatine, lateral margin anterior to contact with the pterygoids** (angulus caudolateralis; Baumel and Witmer 1993; Chandler 1990b, character 2): (0) widens posteriorly; (1) narrows posteriorly. The posterior palatines (past the posterior extent of the maxillopalatine process) broadens before angling medially to articulate with the pterygoids in some species of alcids (e.g., *Alca torda*), while in others (e.g., *Synthliboramphus antiquus*) the palatines remain the same width or get narrower.

**15. Palatine, posterior extension (**Chandler 1990b, character 4): (0) extends to pterygoid articulation; (1) narrow and relatively straight before pterygoid articulation. The lateral margins of the palatines narrow to a thin medially oriented projection before the pterygoid articulation in some species of alcids (e.g., *Synthliboramphus antiquus*).

**16. Vomer, anterior curvature** (Chandler 1990b, character 1): (0) straight; (1) indented. The ventral edge of the anterior vomer is straight in some alcids (e.g., *Brachyramphus marmoratus*), while in some species (e.g., *Alca torda*), the anterior end of the vomer curves dorsally (i.e., is dorsally concave).

**17. Vomer, anterior tip shape:** (0) pointed; (1) bifurcated. The anterior tip of the vomer is pointed in most alcids (e.g., *Alca torda*), while in some species (i.e., *Cepphus grylle*) the anterior tip of the vomer is bifurcated.

**18. Lamina dorsalis, segmentation:** (0) not segmented; (1) segmented. The lamina dorsalis is an extension of the mesethmoid that lies against the ventral side of the frontal (Baumel and Witmer 1993). This osseous feature is segmented and may play some part in bill kinesis in alcids (e.g., *Uria lomvia*). The lamina dorsalis is fused to the rest of the mesethmoid in many charadriiform species (e.g., *Rynchops niger*).

**19. Lamina dorsalis, size:** (0) large; (1) small. The lamina dorsalisof most alcids (e.g., *Alca torda*) is a large (mesethmoid margin interrupted only by suture between it and the lamina dorsalis), triangular, anteriorly pointing structure with a medial crest , while in some species (e.g., *Alle alle*) it is reduced to a small (lamina dorsalis not continuous with margin of mesethmoid, appears to be a separate accessory structure), elongate point.

**20. Frontal, nasal fossa, salt glands, depth** (modified from Strauch 1985, character 5; Chandler 1990b, character 11): (0) shallow; (1) deep. In some species of alcids (e.g., *Alca torda*) deep (i.e., fossa concave) excavations of the frontals are separated by a medial cranial crest, while in some alcids (e.g., *Aethia psittacula*) the nasal fossae are shallower (i.e., flattened or convex).

**21. Frontal, supraorbital rims** (lateral to fossa glandulae nasalis; Baumel and Witmer 1993; modified from Strauch 1985, character 5; Chandler 1990b, character 11): (0) absent; (1) present. This structure associated with the salt glands is completely absent in some alcid species (e.g., *Brachyramphus brevirostris*) while in other alcid species (e.g., *Alca torda*) it is a robust, fully ossified lip that follows along the entire dorsal outline of the orbits.

**22. Mesethmoid, fenestra in nasal capsule anterior to nasofrontal hinge:** (0) small fenestra; (1) large fenestra. In contrast with many closely related charadriiforms (e.g., *Larus marinus*) that have only a small (i.e., fenestra height <=1/3 height of septum) interorbital fenstra, alcids possess a large (i.e., fenestra height >1/3 height of septum) interorbital fenstra.

**23. Mesethmoid, fenestra in nasal capsule anterior to nasofrontal hinge** (0) fenestrated; (1) not fenestrated. Between the lamina dorsalis and the ectethmoid, the mesethmoid of some alcids (e.g., *Fratercula arctica*) is fenestrated. The mesethmoid of other alcids (e.g., *Alca torda*) is not fenestrated.

**24. Foramen opthalmicum internum** (Chu 1998, character 15): (0) absent; (1) present. This foramen, which punctures the interorbital septum near the junction of the mesethmoid and the orbit, is present in all alcids (e.g., *Aethia psittacula*), but is absent in some charardiiforms not closely related to Alcidae (e.g., *Charadrius wilsonia*).

**25. Fonticulus orbitocranialis** (Chu 1998, character 33): (0) not enclosed; (1) enclosed. In most alcids (e.g., *Aethia psittacula*) the mesethmoid does not extend dorsally to fuse with the ventral frontals, thus the fonticulus orbitocranialis is not entirely enclosed by bone. In alcids that have a dorsally ossified mesethmoid (e.g., *Alca torda*), the fonticulus orbitocranialis is a clearly defined foramina near the dorsal margin of the posterior mesethmoid.

**26. Lacrimal, articulation with ectethmoid** (Chu 1998, character 26): (0) occupies entire lateral margin of ectethmiod; (1) occupies only the ventral half of the lateral margin of the ectethmoid. In the Alcidae (e.g., *Alca torda*), the lateral margin of the ectethmoid is dorsoventrally expanded and anteroposteriorly flattened, giving this element a square shape when viewed anteriorly. The lacrimal articulates with the ectethmoid along its entire lateral margin. In many other charadriiforms (e.g., *Sterna maxima*) the ectethmoid tapers laterally to a point. In these taxa the lacrimal extends dorsally from the medially extending ectethmoid.

**27. Lacrimal, position in lateral view:** (0)posteroventrally directed; (1) ventrally directed. With the exception of *Pinguinus impennis* and *Rynchops niger*, the lacrimal of all taxa examined in this study are directed posteroventrally. In contrast, the lacrimal of *Pinguinus impennis* extends ventrally. The condition shared by *Pinguinus impennis* and *Rynchops niger* is not considered homologous here, as the cranium of *Rynchops niger* is extremely derived(with respect to other charadriiforms).

**28. Lacrimal, supraorbital process** (Chu 1998, character 9(0) absent; (1) present. The supraorbital process of the lacrimal (sensu Cracraft 1968), while present in all alcids (e.g., *Cepphus grylle*), is absent in many other charadriiforms (e.g., *Rissa tridactyla*).

**29. Sclerotic ring, shape** (from Strauch 1985, character 7; Chandler 1990b, character 18): (0) narrow, flat ring; (1) wide conical ring with serrated inner edge. The sclerotic ring of most charadriiforms (e.g., *Rynchops niger*) is a flat and narrow. That of puffins (e.g., *Fratercula arctica*), however, is distinctly conical and has a serrated inner edge. Shufeldt (1889) was the first to describe this condition in puffins (Strauch, 1985).

**30. Squamosal, zygomatic process, shape** (Chandler 1990b, character 12): (0) short; (1) elongate. The zygomatic process, which extends ventrolaterally over the articulation of the quadrate with the skull, is a short (i.e., <3× long than wide), relatively rounded structure in many alcids (e.g., *Cepphus grylle*). In some alcids (e.g., *Fratercula arctica*), this process is a long (i.e., >=3× long than wide) pointed projection.

**31. Squamosal, temporal fossa** **depth** (Chandler 1990b, character 19): (0) shallow; (1) deep. The temporal fossa is a shallow (not bordered anteriorly and posteriorly by a distinct lip/crest) depression in most alcids (e.g., *Aethia psittacula*), although, in the a few species (e.g., *Alca torda*) it is a deep (bordered anteriorly and posteriorly by a distinct lip or crest) excavation bordered anteriorly by the temporal crest (Baumel and Witmer 1993).

**32. Squamosal, temporal fossa, medial extent:** (0) not medially extended; (1) separated by a thin flat space; (2) separated only by a thin crest. In many alcids (e.g., *Aethia psittacula*) the temporal fossa is not expressed on the dorsal surface of the skull, although, in some species (e.g., *Alca torda*) the temporal fossa nearly converge on the dorsal surface of the skull. In *Pinguinus impennis* the temporal fossa are very deep and separated only by a thin crest. **Ordered**

**33. Squamosal, temporal fossa, shape of medial margin:** (0) narrow; (1) broad. In species that possess medially expanded temporal fossa (see character 32) the medial–most extent of the temporal fossa varies in alcids from a broad, relatively ‘U-shaped’ curve (e.g., *Alca torda*) to a more pointed, medially narrowing groove (e.g., *Uria aalge*).

**34.** **Supraoccipital foramina** (foramen venae occipitalis externae; Baumel and Witmer 1993; Strauch, 1985, character 6; Chandler, 1990b, character 14)**:** (0) absent; (1) present. Supraoccipital foramina are absent in the skulls of adult Lari and most other groups of charadriiforms (e.g., *Uria aalge*); they are present in some species of alcids (e.g., *Aethia pygmaea*; Strauch, 1985).

**35. Cerebellar prominence** (Chu 1998, character 3): (0) strongly protruding; (1) weakly to moderately protruding. In contrast to charadriiforms with rounded (i.e., posteriorly convex) occipitals (e.g., *Xema sabini*) in which the cerebellar prominence does not protrude a great distance relative to the occipitals, the Alcidae (e.g., *Cepphus grylle*) have anteroposteriorly-flattened occipitals, such that the cerebellar prominence noticeably protrudes posteriorly.

**36. Foramen magnum, dorsal margin shape** (modified from Strauch 1978, character 19: (0) rounded; (1) pointed. The dorsal margin of the foramen magnum of most alcids (e.g., *Alca torda*) is rounded, while this feature in some alcids (e.g., *Alle alle*) is more pointed.

**37. Secondary articulation of mandible** (ala parasphenoidalis; Baumel and Witmer 1993; Strauch 1985, character 4; Chandler 1990b, character 8): (0) well developed; (1) absent. The Lari, auklets, and puffins have a well-developed secondary articulation of the mandible (a.k.a., lateral process of the basisphenoid). The articulation is absent in the murrelets, *Cepphus*, *Alle*, and the auks. Kozlova (1957) reported the presence of the basisphenoid processes associated with this articulation in alcids. Bock (1960) reported the articulation absent in alcids, but did not report which taxa he examined (Strauch, 1985).

**38. Quadrate** (Chandler 1990b, character 10): (0) pneumatic; (1) apneumatic. While the medial surface of the quadrate of many charadriiforms (e.g., *Rynchops niger*) if perforated by a foramina, which leads to the pneumatic interior of the quadrate, the quadrate of all alcids in which this element is known (e.g., *Fratercula corniculata*) is apneumatic.

### Mandible

**39. Mandible, length of symphysis** (modified from Chandler 1990b, character 22): (0) short; (1) long. The left and right rami of the mandible fuse at the anterior end of the mandible. The length of area fused can be either short (i.e., <15% of the total length of the mandible; e.g., *Alca torda*) or long (i.e., >15% of the total length of the mandible; e.g., *Uria aalge*).

**40. Mandible, contact distal to symphysis:** (0) non-contacting; (1) contacting. In most alcids (e.g., *Alca torda*) the mandibular rami are in contact only where fused at the symphysis. In Fraterculini (e.g., *Fratercula arctica*) the mandibular rami, although not fused, remain in contact posterior to the mandibular symphysis.

**41. Mandible, ventral expansion:** (0) absent; (1) present. The mandibles of most alcids (e.g., *Cepphus columba*) are not ventrally expanded. The mandibles of some species (e.g., *Fratercula arctica*) have a pronounced ventral expansion at the anterior end of the mandible (i.e., beak tip).

**42. Mandible, thickening of junction between pars dorsalis and dorsal splenial** (Chu, 1998 character 56): (0) flat to moderate; (1) gross, forming massive longitudinal crista. The dorsomedial surface of the mandible is noticeably thickened in terns (e.g., *Sterna maxima*). In the Alcidae (e.g., *Cepphus columba*) and most other charadriiforms, the medial surface of the mandible is flat (i.e., lateromedially compressed).

**43. Mandible, mediolateral curvature:** (0) laterally concave; (1) laterally convex. The mandibular rami of many alcids (e.g., *Fratercula arctica*) are laterally concave distal to the tip of the bill, while in other alcids (e.g., *Alle alle*) the rami are curved outward or laterally convex.

**44. Prearticular, anterior end** (modified from Chandler 1990b, character 21): (0) forked; (1) not forked. The anterior-most end of the prearticular is forked (i.e.. bifurcates around the distal edge of the rostral mandibular fenestra) in some alcids (e.g., *Alca torda*), while in other alcids (e.g., *Alle alle*) the prearticular is present only ventral to the rostral mandibular fenestra.

**45. Surangular, fenestration:** (0) absent; (1) present. The posterior mandible in many charadriiforms (e.g., *Larus marinus*) is perforated (fenestra caudalis mandibulae; Baumel and Witmer 1993) just anterior to the lateral mandibular cotyla. Some charadriiforms (e.g., *Charadriius wilsonia*) lack this feature. This fenestra provides passage for a nerve, which originates inside the adductor mandibulae pars ventralis muscle, and passes medially through the caudal mandibular foramen and then continues along the medial surface of the angular.

**46. Surangular, fenestration, quantity:** (0) one; (1) two. Most alcids (e.g., *Cepphus grylle*) have a single caudal mandibular fenestra, while the Fraterculini (puffins; e.g., *Fratercula arctica*) and Mancallinae (e.g., *Miomancalla howardi*) are characterized by the presence of two small caudal mandibular fenestrae perforating the dorsal surangular.

**47. Articular, medial articular process** **foramen** (foramen pneumaticum articulare; Baumel and Witmer 1993; Chandler 1990b, character 20): (0) absent; (1) present. An opening in the upper surface of the processus medialis mandibulae that leads to pneumatic spaces in the posterior segment of the mandibular ramus is present in some alcids (e.g., *Cepphus grylle*).

**48. Articular, medial articular process, shape:** (0) anteroposteriorly compressed; (1) dorsoventrally compressed; (2) rounded point. The medial articular process of the mandible, which articulates with the parasphenoid process (Baumel and Witmer 1993, p. 80) in many charadriiforms, varies in shape from an anteroposteriorly-compressed projection (e.g., *Cepphus carbo*) to a dorsoventrally compressed projection (e.g., *Alca torda*).

**49. Articular, medial articular process, orientation:** (0) projects medially; (1) projects posteromedially. The medial articular process of the mandible points medially in some alcids (e.g., *Fratercula cirrhata*), while in other alcids (e.g., *Cepphus grille*) this same process points more posteriorly.

**50. Articular facet in ventral view, shape:** (0) rounded knob; (1) anteromedial projection. In ventral view, the articular facet of the mandible is visible as a small, often rounded knob in some alcids (e.g., *Alca torda*). In some alcids (e.g., *Cepphus grylle*) this facet is more pointed and projects anteromedially.

**51. Articular, retroarticular process** (modified from Chandler 1990b, character 23): (0) absent; (1) present, This characteristic, although present in all alcids, is absent in many other Lari (e.g., *Stercorarius longicaudus*).

**52. Articular, retroarticular process length** (modified from Chandler 1990b, character 23): (0) short; (1) long. In some species (e.g., *Aethia pygmaea*) the dorsoposteriorly projecting process of the cotyla lateralis is long (i.e., as long or longer than the dorsoventral height from the articular facet to the ventral margin of the mandible in lateral view), while in other species (e.g., *Alca torda*) this process is shorter.

### Vertebrae

**53. Atlas, flange on the lateral margins of the arcus atlanticus in dorsal view:** (0) straight; (1) laterally angled. The posteriorly-projecting processes for articulation with the axis project posteriorly in most species of alcids (e.g., *Uria aalge*). In some species of alcids (e.g., *Fratercula arctica*) the zygapophyses angle laterally.

**54. Axis, dorsal extension of neural spine:** (0) short; (1) long. In posterior view, the neural spine of the axis in most alcids (e.g., *Uria aalge*) is short (i.e., less than half of the length of the neural spine extends above the level of the anapophyses), although in some alcids(e.g., *Alca torda*) this projection of the axis is lengthened and extends to a point well above the anapophyses (i.e., more than half of the length of the neural spine extends above the level of the anapophyses).

**55. Thoracic vertebrae, hypapohyses:** (alae cristae ventralis; Baumel and Witmer 1993; modified from Strauch 1985, character 14): (0) not present on any thoracic vertebrae; (1) present on some thoracic vertebrae. The Lari and other non-alcid charadriiforms (e.g., *Larus marinus*) have poorly developed hypapophyses on their thoracic vertebrae. Well-developed hypapophyses, most with bilateral flanged wings, are found in all alcids, but the number of vertebrae on which they occur varies among the species. These structures serve as increased area for attachment of M. longus colli ventralis, are functionally correlated with the strength needed by diving birds (Kuroda 1954). It is hypothesized that a greater number of vertebrae with well-developed hypapophyses is a more derived condition. Similar structures are found in other diving birds such as loons, grebes, penguins, and some anseriforms (Beddard 1898 in Strauch 1985).

**56. Thoracic vertebrae, number of hypapohyses** (crista [processus] ventralis corporis; Baumel and Witmer 1993; modified from Strauch 1985, character 14): (0) well developed on all thoracic vertebrae; (1) well developed on all but last vertebrae; (2) well developed on all but last two vertebrae; (3) well developed on all but last three vertebrae; (4) well developed on all but last four vertebrae; (5) well developed on all but last five vertebrae. **Ordered**

**57. Pygostyle, dorsal margin** (Chu 1998, character 67): (0) dorsally restricted; (1) dorsally expanded. In contrast to the dorsally expanded pygostyle observed in all other charadriiforms examined during this study (e.g., *Larus argentatus*), the Alcidae are characterized by a pygostyle lacking dorsal expansion.

### Sternum

**58. Sternum, coracoidal sulci, separation** (modified from Chandler 1990b, character 35): (0) continuous above dorsal manubrium; (1) sulci separated by manubrium. The sternal articular surface of the coracoid is a continuous, smooth, depression in many charadriiforms (e.g., *Larus marinus*), while in Alcidae (e.g., *Alca torda*) the sulci are separated by the manubrium (i.e., rostrum sterni; Baumel and Witmer 1993).

**59. Sternum, anterior pneumatic foramen:** (0) reduced; (1) pneumatic. The pneumatic foramen located at the anterior end of the sternal basin is reduced to a tiny ‘pin-sized’ hole in alcids (e.g., *Alca torda*). In most other charadriiforms examined (e.g., *Larus marinus*) this feature is a deep pneumatic foramen.

**60. Sternum, sternocoracoidal process, orientation** (Strauch 1985, character 10; Chandler 1990b, character 36): (0) points dorsally; (1) points anteriorly. In the Lari and most other charadriiforms the sternocoracoidal process (process craniolateralis, Baumel and Witmer 1993) of the sternum points dorsally; in the puffins (e.g., *Fratercula arctica*), auklets (e.g., *Aethia psittacula*) and Mancallinae (e.g., SDSNH 26242) it points anteriorly.

**61. Sternum, costal processes, quantity** (Strauch 1985, character 12; Chandler 1990b, character 41): (0) five; (1) six; (2) seven. Although some charadriiforms (e.g., *Glareola maldivarum*) not closely related to Alcidae have five costal processes of the sternum, the Lari and most other charadriiforms (e.g., *Larus marinus*) have six. Some alcids (e.g., *Alca torda*) have seven. **Ordered**

**62. Sternum, width** (modified from Chandler 1990b, character 39): (0) narrow posteriorly; (1) broad posteriorly. In dorsal view the posterior sternum of most alcids (e.g., *Aethia pygmaea*) is lateromedially broader than the anterior sternum (i.e., the area of the sternum proximal to the distal-most costal process), while in other species (e.g., *Alca torda*) the sternum is roughly the same width throughout its length.

**63. Sternum, medial notch** (Strauch 1985, character 8): (0) absent; (1) present. Most charadriiforms (e.g., *Larus marinus*) have a medial sternal notch, but several, including members of the Lari and Alcidae (e.g., *Aethia pusilla*), do not. Distribution of the states among other charadriiforms thus does not indicate which state is primitive in the alcids. Only the puffins (Fraterculini) retain the remnant of the medial sternal notch as a medial sternal fenestra.

**64. Sternum, medial notch, shape:** (0) a notch; (1) a fenestra. Among alcids, only the puffins (Fraterculini) retain the remnant of the medial sternal notch as a medial sternal fenestra.

**65. Sternum. lateral notch, shape** (Strauch 1985, character 9; Chandler 1990b, character 38): (0) a notch; (1) a fenestra. Almost all charadriiforms (including all Lari) have a lateral sternal notch. In the auklets it is reduced to a fenestra, a condition assumed to be a derived state in the Alcidae. Shufeldt (1888, 1889) and Lucas (1890) reported that in the auks the lateral sternal notch tends to become ossified with age. This condition clearly differs from that in the auklets; it is hypothesized to represent merely a variant of the state with the notch present. Kuroda (1954, 1955) illustrated the variation with age of the sternal notching of some alcids (Strauch 1985).

**66. Sternum, lateral notch, anterior extent of incisure:** (0) shallow; (1) deeply incised. In most charadriiforms, the extent to which the lateral sternal notches incise proximally is limited (e.g., *Larus marinus*), while in some charadriiforms (e.g., *Mancalla vegrandis*), these incisures are extensive.

**67. Sternum, posterior extension of carina relative to lateral sternal notches/fenestrae** (modified from Chandler 1990b, character 40): (0) carina extends to distal ends of notches/fenestrae; (1) lateral sternal notches/fenestrae extend posteriorly beyond posterior extent of carina. The length of the carina relative to the posterior extent of the lateral sternal notches/fenestrae of alcids varies from extending to a point about equally posterior to the posterior margins of the lateral sternal notches/fenestrae in some alcids (e.g., *Alca torda*), to a condition in which the lateral sternal notches/fenestrae extend posterior to the carina (e.g., *Aethia cristatella*).

**68. Sternum, supracoracoideus scar, position:** (0) angled medially; (1) straight. In contrast to the condition observed many charadriiforms (e.g., *Sterna maxima*) in which the scar for the supracoracoideus muscle on the ventral surface of the sternum angles medially from the coracoidal sulcus towards the carina, in Alcidae this scar extends posteriorly for almost the entire length of the carina. This feature is correlated with the increased resistance during the upstroke experienced by alcids while flying underwater (Kozlova 1957).

**69. Sternum, posterior margin ossification** (margo caudalis sterni; Baumel and Witmer 1993; modified from Chandler 1990b, character 43): (0) not ossified; (1) ossified. The posterior-most portion of the sternum is an ossified posteriorly projecting structure in many species (e.g., *Uria lomvia*), while in other species (e.g., *Brachyramphus marmoratus*) it is completely unossified and not preserved in dry skeletal specimens.

**70. Sternum, length of area between distal extent of medial fenestra and posterior margin** (modified from Chandler 1990b, character 43): (0) short; (1) long. The ossified area of sternum posterior to the termination of the carina (i.e., the xiphoid) is short (i.e., wider than long) in some alcids (*Alle alle*), while in others (e.g., *Cepphus grylle*) this feature is much longer (i.e., nearly as long or longer than it is wide).

**71. Sternum, length:** (0) short; (1) long. When compared to their immediate outgroup, the Stercorariidae, alcids have an elongated sternum (i.e., sternum >2× long than wide), a character which has been associated with diving (Storer 1960). The greatest length of the sternum (i.e., from the anterior manubrium to the distal xiphoid) is more than two times the width of the sternum across the sternocoracoidal processes in all alcids (e.g. *Alca torda*).

### Furcula

**72. Furcula, symphysis (apophysis), size** (modified from Chandler 1990b, character 30): (0) large; (1) small. A medially oriented crest-like projection characterizes the furcular symphysis of alcids. This crest can be either small (i.e., projects less than the width of individual clavicles at symphysis; *Aethia psittacula*)or large (i.e., projection as wide or wider than that of individual clavicles at symphysis; (e.g., *Brachyramphus marmoratus*).

**73. Furcula, anterior surface of rami** (Strauch 1978, character 41): (0) smooth; (1) grooved. The anterior surface of the furcula dorsal to the apophysis is characterized by a distinct mediolaterally oriented groove or concavity in some alcids (e.g., *Cepphus grylle*; see Strauch 1978, Fig. 22, pg. 310). The furculae of other alcids (e.g., *Alca torda*) are rounded or convex on the anterior surface of the furcula dorsal to the furcular symphysis.

**74. Furcula, cristae on anterior surface of rami:** (0) absent; (1) present. The anterior surface of the furcular rami dorsal to the apophysis is characterized by the presence of small cristae/tubercles in some alcids (e.g., *Alca torda*).

**75. Furcula, curvature of omal extremity** (Chu 1998, character 76): (0) smoothly curving; (1) sharply curved or angled at posterior extremity. The transition from the dorsally extending shaft of the clavicles to the omal extremity of the clavicles in Alcidae (e.g., *Brachyramphus perdix*) is characterized by a distinctly angular bend. The furculae of all other charadriiforms (e.g., *Larus marinus*) examined during the course of this study exhibited a more gently sloping furcular curvature.

**76. Furcula, dorsoventral expansion of omal extremity:** (0) absent; (1) present. Ventral to the coracoidal facet, the clavicles of most alcids (e.g., *Pinguinus impennis*) are dorsoventrally expanded and lateromedially compressed (i.e., bladelike; scapular tuberosity much thinner than clavicular shaft ventral to the coracoidal facet). The clavicles of many other charadriiforms (e.g., *Tryngites subruficollis*) are more circular in cross section and much less dorsoventrally expanded (i.e., scapular tuberosity same width or thicker than clavicular shaft ventral to the coracoidal facet).

**77. Furcula, coracoidal tuberosity, position relative to coracoidal facet** (Chandler 1990b, character 33): (0) medially adjacent to coracoidal facet; (1) separate and anterior to facet. The coracoidal tuberosity contacts the medial margin of the coracoidal facet in alcids (e.g., *Brachyramphus perdix*), while in many other charadriiforms (e.g., *Gygis alba*) this tuberosity is more robust, separate from, and anterior to the coracoidal facet.

### Scapula

**78. Scapula, acromium, attachment of acrocoracoacromiale ligament in proximal view,:** (0) anteriorly oriented pit; (1) laterally oriented scar. In some alcids (e.g., *Uria aalge*), the attachment of the acrocoracoacromiale ligament is an anteriorly oriented excavation of the ventral surface of the acromium process bordered medially by a crest. This same attachment point in other alcids (e.g., *Aethia cristatella*) is rotated laterally and is characterized by a relatively smooth attachment surface.

**79. Scapula, acromium, shape in lateral view:** (0) blunt, rounded; (1) angular, pointed. The acromium process of all extant alcids (e.g., *Uria aalge*) has a pointed proximal tip, while the tip of the acromium in some larids (e.g., *Larus marinus*) is rounded/truncated and does not project anteriorly.

**80. Scapula, scapulotricipital tubercle:** (0) absent; (1) present. A raised process for attachment of m. scapulotriceps on the ventral surface of the scapula (tuberculum m. scapulotricipitis, Baumel and Witmer 1993) just distal to the glenoid facet is present in flightless alcids (e.g., *Pinguinus impennis*) but absent in all extant alcids (e.g., *Alca torda*). The presence of this structure in penguins (Schreiweis 1982), argues in favor of it being correlated with flightlessness.

**81. Scapula, width of distal extremity:** (0) tapering; (1) dorsoventrally expanded. The dorsal margin of the scapula (margo dorsalis; Baumel and Witmer 1993) is expanded dorsally in some species of alcids (e.g., *Cerorhinca monocerata*). The distal ends of the scapulae of other alcids (e.g., *Aethia pygmaea*) are tapered to a point.

**82. Scapula, shape of distal extremity:** (0) curved; (1) angled. In contrast to the gently ventrally curving distal extremity of many charadriiforms (e.g., *Larus argentatus*), the scapulae of all known alcids are characterized by a ventrally directed angular bend proximal to the distal most extremity.

### Coracoid

**83. Coracoid, furcular facet shape** (modified from Chandler 1990b, character 28)**:** (0) oval; (1) rounded. The furcular facet of the coracoid is rounded in thepuffinsand auklets (e.g., *Fratercula arctica*) but is more oval with a vertical long axis in the auks and murres (e.g., *Uria lomvia*).

**84. Coracoid, furcular facet, notch posterior to bicipital tubercle:** (0) absent; (1) present. The ventral margin of the furcular facet is curves dorsally just posterior to the process for the attachment of the bicipital muscle in some species of alcids (e.g., *Uria aalge*). The ventral margin of this feature in other alcids (e.g., *Alca torda*) is gently curved but not notched.

**85. Coracoid, supracoracoidal sulcus:** (0) pneumatic; (1) apneumatic, but deeply undercut; (2) not deeply undercut. The medial side of the distal end or head of the coracoid of some charadriiforms (e.g., *Anous tenuirostris*) are characterized by a pneumatic excavation. The coracoids of all alcids are apneumatic, although the brachial crest is deeply undercut for the passage of the supracoracoideus muscle in some species of alcids (e.g. *Cepphus grille*), while in some alcids (e.g., *Cerorhinca monocerata*) the brachial crest is not deeply undercut (i.e., ventrally concave). **Ordered**

**86. Coracoid, brachial tuberosity, shape:** (0) a tubercle; (1) a crest. The brachial tuberosity is developed as an anteroposteriorly oriented crest in some alcids (e.g., *Cepphus grille*), while in other alcids (e.g., *Aethia psittacula*) the brachial tuberosity is developed simply as a small rounded tubercle positioned roughly at the midpoint on the neck of the coracoid. The term brachial crest is used here to describe the latter condition.

**87. Coracoid, brachial tuberosity, shape in medial view:** (0) approximately straight; (1) distinctly curved. In species that possess a brachial crest rather than a brachial tubercle (see character 88), the crest varies from an approximately straight crest (e.g., *Alle alle*) to a distinctly concave curve (e.g., *Alca torda*).

**88. Coracoid, neck in dorsal view** (Chandler 1990b, character 29): (0) short; (1) long. The neck of alcid coracoids (defined here as the head of the coracoid distal to the distal-most extent of the glenoid facet), which extends medially to articulate with the furcula, is elongate (i.e., considerably longer than wide) in some species (e.g., *Uria aalge*) and gives the neck of the coracoid a rectangular appearance in dorsal view. In other species (e.g., *Fratercula cirrhata*) this neck is shorter (i.e., roughly as wide as it is long) and results in a rather square coracoidal neck.

**89. Coracoid, supracoracoideus scar development:** (0) a distinct ridge; (1) ridge reduced or absent. Contact with the supracoracoideus creates a distinct, medially oriented ridge/scar in most alcids (e.g., *Alca torda*) that gives the shaft of the coracoid a distinctly angular cross-section, while in *Cepphus* this structure is greatly reduced or absent and the cross-section of the coracoid element is more rounded.

**90. Coracoid, supracoracoidal nerve foramen** (Strauch 1985, character 13; Chandler 1990b, character 25): (0) absent; (1) present. The Lari and most other charadriiforms have a coracoidal foramen (e.g., *Pinguinus impennis*); it is absent in some species of alcids (e.g., *Aethia pusilla*; Strauch, 1985).

**91. Coracoid, position of supracoracoidal nerve foramen** (0) distal; (1) proximal. In alcids that possess a coracoidal foramen, the position of this feature is typically near the midpoint of the of the procoracoid process near the shaft of the coracoid (e.g., *Pinguinus impennis*), although in *Cepphus* this foramen is positioned on the extreme anteroproximal edge of the procoracoid process leaving only a very thin strut of bone which forms the dorsal margin of the procoracoid process.

**92. Coracoid, procoracoid process, shape:** (0) rectangular; (1) triangular; (2) wing-shaped. The procoracoid process of some alcids (e.g., *Aethia psittacula*) is ‘strap-like’ and has a roughly rectangular shape, resembling the condition in the outgroup of Alcidae. The shape of the procoracoid process in most alcids (e.g., *Fratercula cirrhata*) is triangular.

**93. Coracoid, tip of procoracoid:** (0) straight; (1) hooked. In *Brachyramphus*, the tip of the procoracoid is hooked anteriorly. This feature is absent in all other Alcidae for which the coracoid is known.

**94. Coracoid, procoracoid process, orientation:** (0) points dorsomedially; (1) points ventromedially; (2) points anteriorly. The tip of the procoracoid process in the auklets (e.g., *Aethia pygmaea*) is hooked, and points ventromedially, while in all other alcids (e.g., *Uria aalge*) the tip of the procoracoid process points dorsomedially. In many other charadriiforms (e.g., *Larus marinus*) the procoracoid process is noticeably hooked to provide passage for the supracoracoideaus tendon, and as a result the tip of the procoracoid process points anteriorly.

**95. Coracoid, procoracoid process, shape of proximal edge:** (0) concave; (1) convex. In posterior view the proximal edge of the procoracoid process (lower or sternal side) in some alcids (e.g., *Cerorhinca monocerata*) curves convexly. The procoracoid process of other alcids (e.g., *Uria aalge*) is concave in curvature.

**96. Coracoid, scar on anterior face of lateral edge of coracoid:** (0) absent; (1) present. Alcids (e.g., *Cepphus grylle*) possess a distinct scar along the anterior surface of the lateral process that is lacking in other charadriiforms (e.g., *Bartramia longicauda*). The exact origin of this scar is unclear, although Fürbringer (1888) discusses several accessory ligaments that attach in this area.

**97. Coracoid, scar extension along anterior surface of lateral process:** (0) extends to sternal articulation; (1) bordered sternally by crest.This scar is less medially and sternally extended and more excavated in the auklets and puffins (e.g., *Fratercula cirrhata*) than in other alcids (e.g., *Alca torda*) in which this scar is less excavated and extends to the sternal margin of the coracoid (i.e., not bordered sternally by a crest).

**98. Coracoid, crest along sternal edge of lateral process:** (0) absent; (1) present. In anterior view the sternal edge of the lateral process of some alcids (e.g., *Uria aalge*) is characterized by a crest or thickening of the sternal margin. This characteristic is absent in some alcids (e.g., *Alca torda*).

**99. Coracoid, lateral (sternocoracoidal) process length** (modified from Strauch, 1985,character 11)**:** (0) elongate, with anteriorly pointing tip; (1) short, with laterally pointing tip. The lateral process of the coracoid is a well developed, elongate projecting process with an anteriorly projecting tip in most alcids (e.g., *Alca torda*) the Laridae and most other charadriiforms; it is absent or poorly developed in some of the auklets (e.g., *Aethia pusilla*). These differences are illustrated by Kuroda (1954: Fig. 7) and are mentioned by Shufeldt 1889 *in* Strauch 1985).

**100. Coracoid, medial sternal process, notch in dorsal margin:** (0) absent; (1) present. The posteromedial margin of the proximal coracoidal shaft just distal to the medial sternal articulation (angulus medialis; Baumel and Witmer 1993) is characterized by a small dorsoanterior oriented projection, giving the shaft a notched appearance in medial view at this point in most alcids (e.g., *Aethia cristatella*). The medial angle is more pointed in other alcids (e.g., *Alca torda*).

**101. Coracoid, sternal facet curvature** (Chandler 1990b, character 26): (0) angled ~135°; (1) angled ~90°; (2) angled >90°. Among the alcids the sternal articulation facet of the coracoid varies, the anterior margin curving roughly 135° in some (e.g., *Cepphus grylle*), roughly 90° in others (e.g., *Alca torda*), and greater than 90° in the auklets (e.g., *Aethia pusilla*). **Ordered**

### Humerus

**102. Humerus, head, distal extent of posterior margin (caput):** (0) convex curve; (1) triangular point. The distally overturned posterior margin of the humeral head of some alcids (e.g., *Cepphus grylle*) is characterized by a distinct, distally extending point. This feature is rounded in most other alcids (e.g., *Alca torda*).

**103. Humerus, dorsal caput, posterior side** (modified from Chandler 1990b; character 49): (0) not notched; (1) notched. The posterior surface of the dorsal caput in proximal view is notched ventral to the dorsal tubercle in some species of alcids (e.g., *Pinguinus impennis*) owing to the posterior projection of the supracoracoidal crest, while in some alcids (e.g., *Fratercula cirrhata*) this transition curves gently anteriorly (i.e., is not notched).

**104. Humerus, deltopectoral crest, distal extension** (modified from Chandler 1990b, character 53): (0) does not extend to midpoint of shaft; (1) extends distally to the midway point of shaft; (2) extends beyond the midpoint of the humeral shaft. The deltopectoral crest extends distally along the anterodorsal margin of the humeral shaft to a point roughly ⅓ to one half of the distance towards the distal end of the shaft in most species of alcids (e.g., *Fratercula arctica*), while in *Pinguinus* the deltopectoral crest extends to the halfway point along the shaft. In *Mancalla* this crest extends distally beyond the midpoint of the shaft.

**105. Humerus, deltopectoral crest, transition to shaft:** (0) smooth; (1) abrupt. As noted by Howard (1982) the deltoid crest merges smoothly with the shaft of the humerus in most alcids (e.g., *Pinguinus impennis*) while in some alcids (e.g., *Fratercula arctica*) this transition is more abrupt or angled.

**106. Humerus, deltopectoral crest, dorsal curvature:** (0) concave; (1) flat. In dorsal view, the area between the dorsal surface of the deltopectoral crest and the dorsal tubercle (i.e., the dorsal shaft distal to the head) is concave in many charadriiforms (e.g., *Creagrus furcatus*). In all alcids except *Alca stewarti* this space is flat or slightly convex in some cases (e.g., *Brachyramphus marmoratus*).

**107. Humerus, impressio coracobrachialis scar, depth** (Chandler 1990b, character 60): (0) very deep; (1) deep; (2) shallow. The scar for attachment of the impressio coracobrachialis muscle in alcids (e.g., *Aethia psittacula*) is a shallow (i.e., smoothly transitions to anterior surface of humeral head), usually rounded impression (e.g., *Alca torda*). This is in contrast to the condition in most other charadriiforms, in which this muscle scar is a very deeply excavated, usually triangular pit. **Ordered**

**108. Humerus, distal edge of bicipital crest, angle with respect to long axis of shaft:** (0) not perpindicular; (1) nearly perpindicular. The ventral edge of the bicipital crest forms a nearly perpindicular angle to the shaft in some species (e.g., *Pinguinus impennis*) while in other species (e.g., *Alca torda*) the bicipital crest is positioned at an obtuse angle with respect to the long axis of the humeral shaft.

**109. Humerus, biciptal crest, transition to shaft:** (0) smooth; (1) notched. This character, noted by Olson and Winker (2009), varies from a condition where (in anterior view) the bicipital crest transitions smoothly onto the humeral shaft (e.g., *Aethia pusilla*) to a condition in which there is a distinct notch or separation between these structures (e.g., *Alle alle*).

**110. Humerus, coracobrachial sulcus, conformation:** (0) open sulcus; (1) closed duct. As noted by Olson and Winker (2009), the coracobrachial sulcus is an open sulcus in most species of alcids (e.g., *Aethia pusilla*), although in some species of alcids (e.g., *Alca torda*) the sulcus is enclosed to form a duct.

**111. Humerus, coracobrachial sulcus, curvature: (0) dorsal; (1) ventral.** The distal most point of the bicipital surface, as defined by the curvature of the coracobrachial sulcus, which curves or angles dorsal to the bicipital crest on the anterior surface of the humerus in some alcids (e.g., *Pinguinus impennis*), while in other alcids (e.g., *Alle alle*) the coracobrachial sulcus and the distal edge of the bicipital surface extend ventrally to terminate where the bicipital crest contacts the ventral surface of the humeral shaft.

**112. Humerus, supracoracoideus scar, depth:** (0) deep; (1) shallow. The attachment for the supracoracoideus muscle on the posterior humerus is a deep (i.e., excavated) scar in puffins (e.g., *Fratercula arctica*) and a shallow (i.e., basically flat) impression in others (e.g., *Cepphus grylle*).

**113. Humerus, supracoracoideus scar, shape:** (0) round; (1) long, proximally broadening; (2) long, does not broaden proximally. The attachment of the supracoracoideus muscle on the proximal humerus of most charadriiforms (e.g., *Larus marinus*) is a rounded scar, while in alcids this scar is distally elongated **(**Crista m. supracoracoidei; Baumel and Witmer 1993). In some alcids (e.g., *Fratercula arctica*) the proximal end of the scar is much broader than the distal end, while in other alcids (e.g., *Alca torda*) the scar is relatively the same width throughout its length.

**114. Humerus, supracoracoideus scar, transition into the secondary pneumatic fossa (pf2):** (0) pf2 borders scar; (1) scar separated from pf2; (2) margo caudalis widely separates pf2 and scar. In most alcids (e.g., *Alca torda*) the dorsal extent of the excavation of the second pneumatic fossa parallels the ventral margin of the supracoracoideus scar. In some species (e.g., *Cerorhinca monocerata*)the excavation for pneumatic fossa 2 is separated from the supracoracoideus scar by a thin, flat, latero-medially oriented projection of the humeral shaft (which is most like the very reduced remains of the margo caudalis). The supracoracoideus attachment point in many other charadriiforms (e.g., *Larus marinus*) is widely separated from the medial portion of the humeral shaft by the margo caudalis and does not extend as far distally as the condition seen in alcids. **Ordered**

**115. Humerus, medial crest between pneumatic fossae, extension relative to the bicipital crest** (modified from Chandler 1990b, character 51)**:** (0) ends proximal to distal-most extension of bicipital crest; (1) crest extends to distal extant bicipital crest; The crest which divides the pneumatic fossae varies in the distance it extends distally towards the distal margin of the bicipital crest. In some species (e.g., *Pinguinus impennis*) this crest terminates proximal to the distal edge of the bicipital crest. In some species (e.g., *Alle alle*) this crest extends to the distal edge of the bicipital crest.

**116. Humerus, primary pneumatic fossa, excavation for insertion of humerotriceps muscle:** (0) absent; (1) present. The interior (i.e., anterior wall) of the ventral pneumatic fossa (fossa pneumotricipitalis ventralis; Baumel and Witmer 1993) in most alcids (e.g., *Alca torda*) is smooth. In *Fratercula cirrhata* this area is characterized by a small ‘eye shaped’ excavation.

**117. Humerus, primary pneumatic fossa, accessory ridge:** (0) absent; (1) present. The interior (i.e., anterior wall) of the ventral pneumatic fossa in most alcids (e.g., *Alca torda*) is smooth. In some Fraterculini (e.g., *Cerorhinca monocerata*) this area is characterized by a small accessory ridge.

**118. Humerus, primary pneumatic fossa, depth:** (0) deeply pneumatic; (1) moderately deep; (2) shallow. In contrast to the deeply pneumatic (i.e., deeper than wide) first fossa of most charadriiforms (e.g., *Larus marinus*), the first pneumatic fossa of most alcids (e.g., *Uria aalge*) is moderate in depth (i.e., ~ as deep as wide). In true auks (e.g., *Alca torda*) the first pneumatic fossa is very shallow and constricted (i.e., less deep than wide). **Ordered**

**119. Humerus, primary pneumatic fossa, shape:** (0) round; (1) oval. The first pneumatic fossa varies in shape from rounded (e.g., *Pinguinus impennis*) to oval (e.g., *Cerorhinca monocerata*).

**120. Humerus, mancalline scar on posterior side of proximal humerus:** (0) absent; (1) present. In *Mancalla* a deep scar extends along the humeral shaft distal to the first pneumatic fossa. This distinct scar, hereafter referred to as the ‘mancalline scar’, is absent in all other charadriiforms. And its its homology is, therefore, uncertain. Although it is possible that this scar may represent an additional insertion point of m. humerotriceps.

**121. Humerus, mancalline scar on posterior side of proximal humerus, conformation:** (0) excavated; (1) raised. The dorsal and ventral borders of the scar on the posterior side of the proximal humerus of *Mancalla* extend parallel to one another in some species (e.g., *Mancalla californiensis*). In other species (e.g., *Miomancalla wetmorei*) these borders converge proximally, giving this scar a more triangular shape.

**122. Humerus, mancalline scar on posterior side of proximal humerus, proximal extension relative to the first pneumatic fossa:** (0) extends within the first pneumatic fossa; (1) scar terminates near the distal margin of the first pneumatic fossa. The proximal extent of this scar varies from a condition in which the scar extends well within the first pneumatic fossa (e.g., *Mancalla californiensis*) to a condition in which this scar terminates near the distal margin of the first pneumatic fossa (e.g., *Miomancalla wetmorei*).

**123. Humerus, mancalline scar on posterior side of proximal humerus, shape:** (0) ridges parallel; (1) ridges converge proximally. The dorsal and ventral borders of the scar on the posterior side of the proximal humerus of *Mancalla* extend parallel to one another in some species (e.g., *Mancalla californiensis*). In other species (e.g., *Miomancalla wetmorei*) these borders converge proximally, giving this scar a more triangular shape.

**124. Humerus, attachment of scapulohumeralis caudalis, position:** (0) medial; (1) ventral. The insertion point of scapulohumeralis caudalis (crus ventrale fossae; Baumel and Witmer 1993) in many alcids (e.g., *Aethia pusilla*) is restricted to the medial border of the first pneumatic fossa (which is also the distal margin of the ventral tubercle). This fossa extends along the ventral margin of the first pneumatic fossa in other alcids (e.g., *Alca torda*).

**125. Humerus, scapulohumeralis caudalis attachment scar, depth:** (0) flat or slightly concave; (1) a deep pit. As noted by Olson and Rasmussen (2001), in alcids the attachment point of the scapulohumeralis caudalis muscle on the margin of the first pneumatic fossa varies from a basically flat or slightly concave surface (e.g., *Alca torda*) to a deeply excavated pit (e.g., *Fratercula arctica*).

**126. Humerus, primary pneumatic fossa, shape of distal edge:** (0) convex; (1) straight; (2) concave. The distal edge of the pneumatic fossa is concave (e.g., *Aethia pusilla*) or straight (e.g., *Alca torda*) in alcids. This feature is convex in most other charadriiforms (e.g., *Charadrius wilsonia*).

**127. Humerus, secondary pneumatic fossa,** (fossa pneumotricipitalis dorsalis; Baumel and Witmer 1993; modified from Strauch 1985, character 17): (0) absent; (1) present. The Lari and most other charadriiforms (e.g., *Larus marinus*) have a well-developed pneumatic fossa II of the humerus. However, in many alcids (e.g., *Alca torda*), it is poorly developed or absent (Strauch 1985).

**128. Humerus, secondary pneumatic fossa, depth** (fossa pneumotricipitalis; Baumel and Witmer 1993; modified from Strauch, 1985, character 17): (0) a deep excavation; (1) a shallow excavation. Among alcids, only the puffins (e.g., *Cerorhinca monocerata*) possess an excavated second pneumatic fossa.

**129. Humerus, secondary pneumatic fossa, division:** (0) absent; (1) present. The pneumatic fossa II of some alcids (e.g., *Aethia pygmaea*) is divided by a medial crest, creating two separate points for muscle insertion. This feature is absent in most alcids (e.g., *Cerorhinca monocerata*).

**130. Humerus, ridge between ventral tubercle and secondary pneumatic fossa:** (0) absent; (1) present. On the posterior side of the humerus in *Brachyramphus* a slight ridge extends distally from underneath the distally overturned head of the humerus and contacts dorsal margin of the ventral tubercle, thus dividing the second pneumatic fossa from the capital groove.

**131. Humerus, ventral tubercle, shape:** (0) long and thin; (1) short and thick. In ventral view the ventral tubercle of some species of alcids (e.g., *Fratercula arctica*) is fairly thin and extends posteriorly to a point roughly level with the posterior extent of the caput. In other alcids (e.g., *Alca torda*) this feature does not extend as far posteriorly, and is more robust.

**132. Humerus, ventral tubercle, lateral margin curvature:** (0) single concavity; (1) double concavity. When viewed ventrally the lateral margin of the ventral tubercle of all alcid species other than *Cerorhinca monocerata* is a single concave curve. This feature in *Cerorhinca monocerata* is characterized by two concave curves. This character is the result of the crus ventrale fossae of *Cerorhinca monocerata* being divided into two sections.

**133. Humerus, ventral tubercle, shape of posterior tip:** (0) rounded or oval; (1) elongate. In *Brachyramphus* the posterior-most extension/point of the ventral tubercle is dorsally expanded into an elongate shape. In other alcids (e.g., *Fratercula arctica*) this feature is rounded or oval in shape.

**134. Humerus, ventral tubercle, ventral margin curvature:** (0) not deeply grooved; (1) deeply grooved. In anterior or posterior view the point at which the ventral tubercle and the ventral margin of the first pneumatic fossa merge varies in its shape from ventrally convex or flat (e.g., *Fratercula corniculata*) to ventrally concave (e.g., *Pinguinus impennis*).

**135. Humerus, m. latissimus dorsi scar, curvature:** (0) straight; (1) curves dorsally. The latissimus dorsi scar in most alcids (e.g., *Fratercula arctica*) extends distally straight down the shaft of the humerus. The latissimus dorsi scar of some alcids (e.g., *Cepphus grylle*) curves anteriorly across the dorsal surface of the humeral shaft.

**136. Humerus, capital groove, anterior expression** (modified from Chandler 1990b, character 52): (0) curved; (1) notched; (2) deep groove. In anterior view the capital groove of most alcids (e.g., *Alca torda*) is visible as a notch on the lateromedial side of the humeral head. In the aukets (e.g., *Ptychoramphus aleuticus*) the capital groove is not expressed anteriorly, resulting in a convexly curved shaped lateromedial side of the humeral head. In the Mancallinae alcids (e.g., *Mancalla cedrosensis*) the capital groove communicates with the ligamental furrow, and is expressed as a deep groove in the ventral margin of the anterior humeral head. **Ordered**

**137. Humerus, capital groove, width:** (0) wide; (1) constricted. In all alcids (e.g., *Fratercula arctica*) except *Mancalla* the capital groove is an open ‘U’ shaped groove. Only in *Mancalla* does the caput overhang the capital groove, giving the proximal wall of the capital groove a convex shape, and constricting this passageway.

**138. Humerus, capital groove, shape:** (0) ‘U’ shaped; (1) pointed anteriorly. In ventral view the capital groove is ‘U’ shaped in most alcids (e.g., *Aethia* psittacula). In *Mancalla* the capital groove is constricted anteriorly.

**139. Humerus, orientation of head relative to shaft:** (0) in line with shaft; (1) rotated anteriorly. As noted by Miller (1933), the humeral head of most alcids (e.g., *Alca torda*) is in-line with the shaft of the humerus, while the ventral portion of the humeral head of mancalline alcids (e.g., *Mancalla cedrosensis*) is rotated anteriorly.

**140. Humerus, longitudinal shape of shaft,:** (0) sigmoidal; (1) arced. As noted by Lucas (1901), the shaft of most alcids (e.g., *Alca torda*), when viewed laterally, is slightly sigmoidal in shape, while the shaft of the mancalline alcids (e.g., *Mancalla californiensis*) is arced.

**141. Humerus, cross-sectional shape of shaft,:** (0) rounded; (1) semi-rounded; (2) flattened. As noted by Howard(1978, 1982), the humeral shaft of most alcids (e.g., *Alca torda*) is flattened in cross-section (flattened oval). The shaft of some alcids (e.g., *Cepphus grylle*) is more rounded (i.e., semi-rounded) in cross-section. The humeral shaft of other charadriiforms (e.g., *Larus marinus*) is rounded in cross-section. **Ordered**

**142. Humerus, shaft thickness:** (0) robust; (1) gracile. The thickness of the humeral shaft varies from robust (i.e., width of shaft in anterior view greater than or equal to half the width of the humeral head; e.g., *Brachyramphus marmoratus*) to gracile (i.e., width of shaft in anterior view less than or equal to half the width of the humeral head; e.g., *Alle alle*).

**143. Humerus, dorsal supracondylar process, shape:** (0) large dorsally pointing projection;(1) small dorsally pointing projection; (2) smoothly transitioning; (3) square; (4) rounded knob. The attachment point for M. extensor carpi along the dorsal margin of the distal humerus projects dorsally away from the shaft in many charadriiforms (e.g., *Larus marinus*; state 0) while in all alcids this feature is elongated along the shaft of the humerus medially and does not project as far dorsally. States within Alcidae include: (1) a small dorsally projecting point (e.g., *Alca torda*), (2) square, ~ 90° contact with shaft (e.g., *Fratercula arctica*) (3) smoothly transitioning to the shaft (e.g., *Pinguinus impennis*), In *Mancalla* this process is a rounded knob that is separated from the distal extent of the dorsal supracondylar prominence (crest) by a gap.

**144. Humerus, dorsal supracondylar process, length:** (0) short; (1) long. The dorsal supracondylar process of most alcids (e.g., *Aethia pygmaea*) is short (i.e., the proximodistal length measured from the distal end of the humerus to the proximal termination of the crest on the humeral shaft is shorter than the greatest distal width of the humerus measured from the entepicondyle to the dorsal condyle). The dorsal supracondylar process of some alcids (e.g., *Mancalla lucasi*) extends further proximally onto the humeral shaft.

**145. Humerus, dorsal sulcus:** (0) continuous; (1) divided. The sulcus for passage of extensor metacarpi radialis, which runs between the dorsal supracondylar process and the dorsal condyle is continuous in all alcids (e.g., *Pinguinus impennis*) except the Fraterculini (e.g., *Fratercula arctica*), in which this sulcus is divided by a bony crest, forming a round pit on the posterior edge of the dorsal condyle.

**146. Humerus, ventral epicondyle, orientation relative to shaft:** (0) flared ventrally; (1) nearly straight. As noted by Olson and Rasmussen (2001), in anterior view the ventral margin of the ventral epicondyle is flared ventrally in *Fratercula arctica*, but is nearly straight in *Cepphus grylle*.

**147. Humerus, tricipital sulci, width** (modified from Chandler 1990b, character 54): (0) scapulotricipital sulcus narrower than humerotricipital sulcus; (1) sulci of equal width; (2) scapulotricipital sulcus broader than humerotricipital sulcus. The scapulotricipital sulcus of Fraterculini species (e.g., *Cerorhinca monocerata*) is narrower than humerotricipital sulcus. In most other alcid taxa (e.g., *Alca torda*) these sulci are of roughly equal width. *Alle alle* is the only species of extant alcid in which the scapulotricipital sulcus is broader than the humerotricipital sulcus.

**148. Humerus, tricipital crest, orientation:** (0) straight, projects posteriorly; (1curved dorsally over scapulotricipital sulcus. The crest that divides the tricipital sulci (humerotricipital and scapulotricipital sulci) is a low posteriorly projecting ridge in most alcids (e.g., *Uria aalge*). In *Mancalla* this ridge veers dorsally and merges with the dorsal margin of the scapulotricipital sulcus on its lateral side.

**149. Humerus, humerotricipital sulcus, shape in distal view:** (0) flattened; (1) ‘U’ shaped or curved. In distal view the humerotricipital sulcus of all alcids other than *Alle alle* and *Uria aalge* is curved or ‘U’ shaped.

**150. Humerus, tricipital fossae:** (0) absent; (1) present. The scapulotricipital and humerotricipital sulci of Mancallinae are characterized by fossae positioned at the proximal end of the sulci. The sulci of other alcids transition smoothly onto the posterior face of the humeral shaft.

**151. Humerus, relative distal extension of condyles** (Chandler 1990b, character 61): (0) level; (1) distal extent of dorsal condyle proximal to distal extent of ventral condyle. The dorsal condyles of all extant alcids (e.g., *Alca torda*) are situated slightly proximal to the ventral condyle. The condyles of most other charadriiforms (e.g.,*Gygis alba*) extend distally an equal distance.

**152. Humerus, ventral condyle in distal view, posterior trochlear process:** (0) absent; (1) present. As noted by Marsh (1870) in the original description of *Cataractes antiquus* a posterodorsally-projecting tubercle is present on the ventral condyle; projecting into the sulcus between the ventral condyle and the saddle that defines the distal extent of the humerotricipital sulcus. This characteristic is also present in *Pinguinus*, but is lacking in all other alcids (e.g., *Alca torda*). This character has also been noted in penguins and plotopterids (Marples 1952; Ksepka et al. 2006).

**153. Humerus, ventral condyle, shape** (Chandler, 1990b, character 59)**:** (0) rounded; (1) flattened. The anterior face of the ventral humeral condyle is flattened in most alcids (e.g., *Pinguinus impennis*), while the ventral condyle is rounded in all other charadriiforms examined during this study (e.g., *Larus argentatus*).

**154. Humerus, separation of humeral condyles:** (0) absent; (1) present. In distal view the humeral condyles of *Brachyramphus* are separated, whereas the ventral margin of the dorsal condyle and the dorsal margin of the ventral condyle of other alcids (e.g., *Synthliboramphus antiquus*) contact one another.

**155. Humerus, tubercle adjacent to dorsal condyle:** (0) absent; (1) present. As noted by Howard (1982), a small rounded tubercle lies ventral to the dorsal condyle along the ventral margin of the brachial impression in some alcids (e.g. *Mancalla californiensis*), but is absent in most species of alcids (e.g., *Alca torda*).

**156. Humerus, tubercles dorsal to scapulotricipital groove:** (0) absent; (1) present. Many alcids (e.g., *Brachyramphus perdix*) possess a tubercle along the dorsal border of the scapulotricipital sulcus. In alcids this tubercle is located distal to paired fossae that lye between the raised dorsal margin of the scapulotricpital sulcus and the dorsal sulcus.

**157. Humerus, tubercles dorsal to scapulotricipital groove, quantity:** (0) a single tubercle; (1) paired tubercles. Rather than a single tubercle, some alcids (e.g., *Mancalla cedrosensis*)possess two tubercles dorsal to the scapulotricipital sulcus.

**158. Humerus, ventral supracondylar tubercle (anterior ligament scar):** (0) triangular; (1) rounded. On the anterior surface of the distal shaft of the humerus ventral to the brachialis scar, the ventral supracondylar tubercle, which is the attachment for the ventral collateral ligament, varies in its shape from triangular (e.g., *Brachyramphus marmoratus*), to an oval/rounded pit (e.g., *Alca torda*).

**159. Humerus, pit associated with anterior ligament scar:** (0) absent; (1) present. A small scar on the distal humerus marks the attachment point of the M. pronator sublimis in some species of alcids (e.g., *Alca torda*), but is absent in many species (e.g., *Uria aalge*).

**160. Humerus, position of pit adjacent to anterior ligament scar:** (0) proximal; (1) ventral; (2) detached. The position of the small pit, which marks the origination point of the M. pronator sublimis varies in its position. In some species (e.g., *Aethia pygmaea*) this feature is located at the proximal tip of the anterior ligament scar, while in other species of alcids (e.g., *Aethia psittacula*) it is located along the dorsal margin of this scar. In some other charadriiforms (e.g., *Phaetusa simplex*), this scar is detached from the anterior ligament scar.

### Radius

**161. Radius, bicipital tubercle:** (0)reduced;(1)distinct. The auklets (e.g., *Ptychoramphus aleuticus*) and murrelets (e.g., *Synthliboramphus antiquus*) lack the distinct bicipital tubercle found in other alcids (e.g., *Alca torda*).

**162. Radius, bicipital tubercle, shape:** (0) a crest; (1) a round tubercle. The shape of the bicipital tubercle in Alcidae (e.g., *Alca torda*) is an elongated crest-like structure, rather than the rounded tubercle of other charadriiforms (e.g., *Larus marinus*).

**163. Radius, bicipital tubercle, position:** (0) contacts papilla; (1) separate. The bicipital tubercle of most alcids (e.g., *Uria lomvia*) is a swollen area along the distal margin of what Howard (1929) termed the ligamental papilla. In a few alcids (e.g., *Cepphus columba*) the bicipital tubercle is a separate structure, positioned distally and separated from the ligamental papilla.

**164. Radius, sulcus tendinosus** (Chu 1998, character 102): (0) not divided; (1) divided lengthwise by a crest. The tendinal groove located on the dorsal side of the distal radius is divided by a crest in some species of alcids (e.g., *Synthliboramphus antiquus*). This feature is lacking in the charadriiform outgroup taxa examined and also in the auklets (e.g., *Aethia pusilla*).

**165. Radius, notch in distal end:** (0) absent; (1) present. In anterior view, the crest associated with the scapho-lunar facet of some alcids (e.g., *Aethia pygmaea*) extends far enough distally so that a notch is formed between that crest and the ventral-most articulation surface of the distal radius with the radiale. In other alcids (e.g., *Alle alle*) the crest on the anterior surface of the radius transitions smoothly into the distal end of the radius.

### Ulna

**166. Ulna, olecranon, length:** (0) long; (1) short; (2) truncate. The olecranon of most alcids (e.g., *Alca torda*), is a long (i.e., projects well past the medial extent of the ventral cotyla) medially projecting point. In some species (e.g., *Pinguinus impennis*) the olecranon is truncated (i.e., does not extend past the medial extent of the ventral cotyla), while the condition in other alcids (i.e., *Aethia pusilla*) is intermediate (i.e., olecranon short). **Ordered**

**167. Ulna, olecranon, curvature:** (0) flares posteriorly; (1) curves anteriorly. In dorsal view the posterior margin of the ulnar head of most alcids (e.g., *Pinguinus impennis*) flares posteriorly to form the posterior edge of the olecranon, while in some species (e.g., *Alca torda*) this margin curves anteriorly.

**168. Ulna, ventral collateral ligament tubercle, shape:** (0) triangular; (1) rounded. The scar for the attachment of the ventral collateral ligament is triangular in some alcids (e.g., *Alca torda*) and more rounded in others (e.g., *Aethia psittacula*).

**169. Ulna, crest extending from the ventral cotyla to the anterior margin of the ventral collateral ligament tubercle** (modified from Chandler 1990b, character 63): (0) absent; (1) present. The ventral cotyla of the proximal ulna is separate from the scar for the attachment of the ventral collateral ligament in some species of alcids (e.g., *Cepphus grylle*). In other alcids (e.g., *Alle alle*) a crest extends laterally from the ventral cotyla and contacts the anterior margin of the collateral ligament scar.

**170. Ulna, crest extending from the ventral cotyla to the posterior margin of the ventral collateral ligament tubercle:** (0) absent; (1) present.Although most alcids (e.g., *Alca torda*) lack a crest, which extends from the ventral cotyla to contact the posterior margin of the ventral collateral ligament scar, several alcids (e.g., *Brachyramphus brevirostris*) possess this character.

**171. Ulna, dorsal cotylar process, anterior margin shape:** (0) rounded; (1) straight. The dorsal condyle of alcids is bordered on the posterior margin by a posteriorly projecting bladelike process for attachment of the scapulotriceps muscle. The anterior margin of this feature in dorsal view can be either rounded (e.g., *Alca torda*) or straight (e.g., *Fratercula cirrhata*).

**172. Ulna, dorsal cotylar process, development** (Chu 1998, character 98): (0) poorly developed; (1) well developed. The dorsal cotylar process of all alcids (e.g., *Alca torda*) is a distinct anteriorly expanded structure when compared to the less developed condition observed in other charadriiforms (e.g., *Larus marinus*).

**173. Ulna, proximal radial depression, shape:** (0) a round pit; (1) a triangular pit; (2) broad and flat. In contrast to the distinctly triangular shape of the proximal radial depression of most charadriiforms (e.g., *Larus marinus*), the proximal radial depression of all extant alcids (e.g., *Uria aalge*) is a round pit situated distal to the ulnar cotylae. Insome charadriiforms not closely related to Alcidae (e.g., *Bartramia* longicauda) the radial depression is broad and flat.In *Miomancalla wetmorei* the proximal radial depression is a broad flat space bordered dorsally and ventrally by distinct crests that occupies the entire anterior surface of the ulna (Howard 1982). **Ordered**

**174. Ulna, brachial impression, breadth:** (0) thin; (1) broad. As noted by Howard (1982) the brachial impression on the proximal ulna of some alcids (e.g., *Alca torda*) is a relatively thin scar (i.e., does not comprise more than half the width of the ulnar shaft), while in some species (e.g., *Fratercula arctica*) this feature is broader (i.e., comprises more than half of the width of this proximal portion of the ulna).

**175. Ulna, intramuscular line:** (0) non-distinct; (1) distinct, raised ridge. As noted by Olson (1981), an inter-muscular line runs between the proximal radial depression and the nutrient foramen. This inter-muscular line is non-distinct and often barely visible in many species of alcids (e.g., *Alle alle*), while in others this feature is distinct and raised (e.g., *Pinguinus impennis*).

**176. Ulna, shaft, shape:** (0) rounded; (1) semi-flattened; (2) flattened. As noted by Howard (1978) the ulnae of most alcids (e.g., *Alca torda*) exhibit the flattening typical of the pectoral elements of wing-propelled divers, while the ulnae of some alcids (e.g., *Cepphus grylle*) are more rounded (i.e., semi-flattened) in cross section. The ulna of other charadriiforms (e.g., *Larus marinus*) are more rounded in cross section. **Ordered**

**177. Ulna, dorsal condyle, shape:** (0) rounded; (1) angular. The entire posterior margin of the dorsal ulnar condyle of some alcids (e.g., *Pinguinus impennis*) is rounded, while in other alcids (e.g., *Alca torda*) the dorsal condyle has an angular bend distal to the contact with the ulnar shaft.

**178. Ulna, carpal tubercle, shape:** (0) flat or angled distally; (1) concave. The distal margin of the carpal tubercle of some alcids (e.g., *Alca torda*) is flat or angles slightly distally in some specimens. In *Cepphus* this surface is concave, giving the distal surface of the carpal tubercle a hooked appearance.

**179. Ulna, sulcus intercondylaris:** (0) concave; (1) flat. In distal view the groove between the ulnar condyles is a concave ‘U’ shaped depression in some species (e.g., *Alca torda*). In other species (e.g., *Pinguinus impennis*) the posterior surface of the ventral condyle angles anteriorly from the sulcus towards the ventral surface of the ulna, forming a flat almost 90° angle between the condyles (i.e., ‘stairstep-like’).

**180. Ulna, length:** (0) longer than carpometacarpus; (1) shorter than carpometacarpus. The ulnae of most alcids (e.g., *Cepphus grylle*) are longer than their carpometacarpi, while the ulnae of Mancallinae are shorter (e.g., *Mancalla cedrosensis*). This condition is not known in other flightless birds, but interestingly, is found in some hummingbirds.

**181. Ulna, ventral condyle, orientation** (Chandler 1990b, character 64): (0) directed posteriorly; (1) directed posteroventrally. The ventral condyle of *Mancalla* species (e.g., *Mancalla cedrosensis*)is directed posteroventrally, while in all other charadriiform taxa examined during this study, the ventral condyle is directed posteriorly.

**182. Ulna, dorsal condyle, distal extension** (Chandler 1990b, character 65): (0) dorsal condyle extends distal to ventral condyle; (1) level. The dorsal condyle of most alcids (e.g., *Alca torda*) extends distally the same distance as the ventral condyle. In *Mancalla* (e.g., *Mancalla cedrosensis*) the dorsal condyle extends further distally than the ventral condyle.

### Carpometacarpus

**183. Carpometacarpus, extensor process of metacarpal 1:** (0) present; (1) absent. The carpometacarpi of all alcids except *Mancalla* (e.g., *Mancalla cedrosensis*) have an extensor process on the anterior margin of metacarpal 1.

**184. Carpometacarpus, metacarpal 1, length** (modified from Chandler 1990b, character 67): (0) short; (1) long. The first metacarpal of most alcids (e.g., *Alca torda*) extends distally about one third of the length of metacarpal 2, while in *Mancalla* species(e.g., *Mancalla cedrosensis*) the extensor process terminates approximately at the midpoint of metacarpal 2.

**185. Carpometacarpus, extensor process of metacarpal 1, anterior margin shape** (modified from Strauch 1985, character 18; Chandler 1990b, character 69): (0) rounded knob; (1) flat. The extensor process of the carpometacarpus is a short, rounded knob in the Lari and most other charadriiforms (e.g., *Fratercula arctica*). In the Alcini (e.g., *Alca torda*) the anterior margin of the process of metacarpal 1 is flat in comparison with other extant alcids (yet still slightly anteriorly convex).

**186. Carpometacarpus, proximal intermetacarpal spatium, position relative to the distal extent of metacarpal 1** (modified from Chandler 1990b, character 71): (0) symphysis distal to MC1; (1) symphysis level with MC1; (2) symphysis proximal to MC1. In relation to the pollical facet, the symphysis can be either distal to it (e.g., *Aethia cristatella*), level with it(e.g., *Cepphus carbo*), or proximal to it (e.g., *Synthliboramphus antiquus*).

**187. Carpometacarpus, posterior extension of ventral trochlear margin relative to metacarpal III** (modified from Chandler 1990b, character 70): (0) ventral trochlear margin of carpometacarpus extends posteriorly to metacarpal III (e.g., *Alca torda*); (1) ventral trochlear margin and metacarpal III extend an equal distance posteriorly (e.g., *Fratercula arctica*); (2) ventral trochlear margin does not extend as far posteriorly as metacarpal III (e.g., *Aethia pusilla*).

**188. Carpometacarpus, pisiform process, development:** (0) distinct; (1)reduced. The Pisiform process of most alcids (e.g., *Miomancalla wetmorei*) is a distinct ventral projection. The Pisiform process of *Mancalla cedrosensis* is reduced to a small scar. Similar to the condition observed in penguins, the reduction of this feature in *Mancalla* may be related to the stiffening of the wing that is associated with the lack of these highly specialized wing propelled divers need to flex the manus.

**189. Carpometacarpus, distal end of tendinal groove** (i.e., sulcus interosseous; Baumel and Witmer 1993): (0) a sulcus; (1) a bony canal. The sulcus occupied by the tendons of the interossei muscle on the distal end of the dorsal carpometacarpus varies from a distally open-ended groove (e.g., *Uria lomvia*), to a partially or fully roofed bony canal (e.g., *Aethia pusilla*).

**190. Carpometacarpus, minor digit articulaton:** (0) level with facies articularis digitalis major; (1) proximal to facies articularis digitalis major. The articulation surface of the minor digit (III:1) is located proximally to the articulation surface for the major digit (II:1) in some species (e.g., *Pinguinus impennis*), whereas in other species (e.g., *Alca torda*) the articulation surfaces are approximately level.

### Major Phalanx

**191. Manual digit II, phalanx 1, fenestration:** (0) absent; (1) present. The major phalanx of many charadriiforms (e.g., *Larosterna inca*) is penetrated by two fenestrae. These fenestrae are absent in all alcids.

**192. Manual digit II, phalanx 1, shape of process on dorsal surface of the distal end:** (0) rounded; (1) rectangular. A bladelike process projects posteriorly from the distal end of the first phalange of the second digit. In dorsal view this process varies from rounded (e.g., *Aethia pusilla*) to rectangular (e.g., *Alca torda*).

**193. Manual digit II, phalanx 1, length:** (0) <1/2 length of carpometacarpus; (1) >1/2 length of carpometacarpus. The greatest length of the major phalanx of some charadriiforms (e.g., *Sterna maxima*) is >1/2 the greatest length of the carpometacarpus. The length of the major phalanx is <1/2 the length of the carpometacarpus in all alcids except *Pinguinus impennis*, in which the relative length of the wing elements has been reduced in association with flightlessness.

### Pelvis

**194. Ilium, pre-acetabular ilium, lateral expansion:** (0) not expanded, narrow; (1) expanded laterally, spatulate. As noted by Kuroda (1954) the anterior ends of the pre-acetabular blades of the ilium are laterally expanded (i.e., wider) in some species of alcids (e.g., *Uria lomvia*) than others (e.g., *Synthliboramphus antiquus*).

**195. Synsacral strut extending to acetabulum** (Strauch 1985, character 15; Chandler 1990b, character 47): (0) absent; (1) present. In most charadriiforms (e.g., *Aethia* pygmaea) a strut or brace extends from the fused sacral-caudal vertebrae towards the acetabulum. In alcids this strut may be well developed (contra Strauch, 1978), it may be reduced to a very slight ridge, or it may be completely absent (e.g., *Alca torda*; Strauch, 1985).

**196. Renal depression** (Chandler 1990b, character 44): (0) broad; (1) narrows posteriorly. The renal depression on the ventral side of the ilium maintains a relatively constant width in some alcids (e.g., *Aethia pusilla*) while in other alcid species (e.g., *Uria aalge*) the renal depression narrows posteriorly.

**197. Antitrochanteral sulcus, distal extension:** (0) terminates at antitrochanter; (1) extends past antitrochanter. The antitrochanteral sulcus (sulcus antitrochantericus, Baumel and Witmer 1993) is bordered medially by a crest that extends distal to the acetabulum in some alcids (e.g., *Alca torda*). This crest curves laterally and ends at, or just past, the distal extent of the antitrochanter in other species of alcids (e.g., *Cepphus grylle*).

**198. Iliosynsacral suture:** (0) fused; (1) perforated. The contact between the lateral processes of the sacral vertebrae and the ilium, termed the iliosynsacral suture (sutura iliosynsacralis, Baumel and Witmer 1993), is fused along its entire margin in some alcids (e.g., *Cepphus columba*), while in other species (e.g., *Alca torda*) this suture is non-continuous (i.e., perforated by spaces between the lateral processes of the sacral vertebrae). This feature is distinct from the foramina intertransversaria of Baumel and Witmer (1993), which are located medially to the iliosynsacral suture.

**199. Ilium, post acetabular dorsal iliac crest:** (0) broadens; (1) narrows. The dorsal iliac crest (crista dorsolateralis ilii, Baumel and Witmer 1993) broadens past the acetabulum and angles posterolaterally in some species of alcids (e.g., *Uria aalge*), while in other species of alcids (e.g., *Alca torda*) the post acetabular area of the ilium is narrower (i.e., tapers posteriorly).

**200. Ilium, dorsolateral iliac spine orientation:** (0) dorsal; (1) dorsolateral. The dorsolateral iliac spine (spina dorsolateralis ilii, Baumel and Witmer 1993) is oriented so that its surface faces much more dorsally in some species of alcids (e.g., *Cepphus columba*) than in others (e.g., *Uria aalge*).

**201. Ilium, dorsolateral iliac spine shape:** (0) pointed; (1) square. The dorsolateral iliac spine (spina dorsolateralis ilii, Baumel and Witmer 1993) is pointed in all alcids (e.g., *Cepphus columba*) except *Aethia* (e.g., *Aethia pygmaea*).

**202. Ischium, relative length of ischial angle and posterior projection** (Strauch 1985, character 16; Chandler 1990b, character 46): (0) ischial angle much longer; (1) both structures about the same length. In the Lari and most other charadriiforms (e.g., *Alca torda*) the ischial angle is much longer than the posterior projection of the ilium; in the auklets (e.g., *Aethia pusilla*) the length of the ischial angle is much reduced, and the structures are almost the same length. These differences also are indicated by Storer’s (1945a) measurements of alcid skeletons (Strauch 1985).

**203. Pelvis, width:** (0) broad; (1) narrow. In contrast to the broad (i.e., length of pelvis from anterior-most illium to distal point of the dorsal iliac spine <= 2× width across pelvis at antitrochanter) pelvi of many other charadriiforms (e.g., *Sterna maxima*), the pelvi of all alcids are narrow (i.e., length of pelvis from anterior-most illium to distal point of the dorsal iliac spine>2× width across pelvis at antitrochanter).

### Femur

**204. Femur, trochanteric ridge, shape:** (0) convex; (1) straight; (2) concave. In most alcids the anterior margin of the femoral trochanter in lateral view is straight (e.g., *Alca torda*) or convex (e.g., *Alle alle*). In puffins (e.g., *Fratercula arctica*) the trochanter is slightly concave.

**205. Femur, trochanteric ridge, length:** (0) long; (1) short. As noted by Miller (1937) the trochanteric crest of the femur varies in the extent to which it extends distally down the lateral shaft of the proximal femur from short (i.e., extends distally <2× width of the lateral surface of femoral head; e.g., *Synthliboramphus hypoleucus*) to long (i.e., extends distally >=2× width of the lateral surface of femoral head; e.g., *Alca torda*).

### Tibiotarsus

**206. Tibiotarsus, cnemial crests, shape in proximal view:** (0) ‘T’ shaped; (1) ‘L’ shaped. In some alcids (e.g., *Aethia cristatella*) the medial cnemial crest extends posteriorly along the medial margin of the femoral articulation surface. This gives this feature a ‘T’ shape in proximal view. Some alcids (e.g., *Uria lomvia*) lack this posterior extension and as a result the cnemial crests appear ‘L’ shaped in proximal view.

**207. Tibiotarsus, cnemial crests, distal extent** (Chandler 1990b, character 74): (0) anterior crest extends further distally than lateral cnemial crest; (1) both extend distally about equal. The distal extent of the cnemial crests is roughly equal in some alcids (e.g., *Alle alle*) while the anterior cnemial crest extends further distally in some alcids (e.g., *Aethia psittacula*).

**208. Tibiotarsus, lateral cnemial crest** **orientation:** (0) directed anterolaterally; (1) directed laterally. In proximal view the external cnemial crest of some alcids (e.g., *Alca torda*) is directed anterolaterally, while the lateral cnemial crest of other alcids (e.g., *Aethia psittacula*) is directed laterally, which results in a more constricted incisura tibialis.

**209. Tibiotarsus, notch in lateral margin of medial condyle** (Chandler 1990b, character 75): (0) absent; (1) present. The distal most portion of the medial condyle of many alcids (e.g., *Pinguinus impennis*) is notched in lateral view. Other alcids (e.g., *Alca torda*) lack this feature. This notch is a common feature in many charadriiforms (e.g., *Larus marinus*) and its absence is therefore considered derived among alcids.

**210. Tibiotarsus, lateral projection of crest lateral to the groove for peroneus profundus tendon, posterior view:** (0) a distinct projection; (1) not visible in dorsal view. The lateral edge of the groove for the peroneus profundus tendon projects far enough laterally in some species of alcids (e.g., *Alca torda*) to be visible in posterior view.

**211. Tibiotarsus, supratendinal bridge:** (0) not fully ossified; (1) fully ossified. The supratendinal bridge of all alcids (e.g., *Alle alle*) except *Pinguinus* is ossified.

**212. Tibiotarsus, length:** (0) <2× greatest length of tarsometatarsus; (1) >2× greatest length of tarsometatarsus. The greatest length of the tibiotarsus is greater than two times the greatest length of the tarsometatarsus in most alcids (e.g., *Alca torda*), but in some species of alcids (e.g., *Synthliboramphus antiquus*) the tibiotarsus is less than twice the length of the tarsometatarsus.

### Tarsometatarsus

**213. Tarsometatarsus, tendinal canal No. 1 of hypotarsus, conformation** (modified from Strauch 1978, character 64; Chandler 1990b, character 82): (0) deep channel; (1) bony canal. The pattern of the canals in the hypotarsus of charadriiforms is discussed by Strauch (1978). In most charadriiforms canal No. 1 is a bony canal; in the Lari it is either a bony canal or a deep channel. In the Alcidae it may be a bony canal (e.g., *Aethia pusilla*), or a deep channel (e.g., *Alca torda*). The bony canal in charadriiforms is hypothesized to be primitive (Strauch 1978). More open canals in the hypotarsus have been linked with greater specialization and probably represent derived states (Harrison 1976 in Strauch 1985). In charadriiforms, tendinal canal No. 1 provides passage for m. flexor digitorum longus (Strauch 1978).

**214. Tarsometatarsus, tendinal canal No. 2 of hypotarsus, position** (modified from Strauch 1978, character 65): (0) posterior to tendinal canal 1; (1) confluent with tendinal canal 1; (2) posteromedial to tendinal canal 1, bordered medially by medial hypotarsal crest. In alcids that have an enclosed tendinal canal 1, this canal is positioned anterior to tendinal canal 2 (e.g., *Alle alle*). In alcids that do not have an enclosed tendinal canal 1 (e.g., *Alca torda*), the tendons for M. flexor digitorum longus, and M. flexor perforatus digit IV and/or M. flexor perforans et et perforatus digiti II, presumably run within what has been designated tendinal canal 1 (Strauch 1978). The second tendinal canal of some other charadriiforms (e.g., *Charadrius wilsonia*) is located posteromedial to tendinal canal 1 along the medial border of the medial hypotarsal crest.

**215. Tarsometatarsus, tendinal canal No. 3 of hypotarsus, conformation** (modified from Strauch 1978, character 66): (0) open groove; (1) mostly or completely enclosed bony channel. In the taxa examined, the third tendinal canal of the hypotarsus varies from a shallow groove (e.g., *Alca torda*) to a partially or fully enclosed bony canal (e.g., *Cepphus grylle*). In charadriiforms, the tendinal canal No. 3 provides passage for m. flexor hallicus longus (Strauch 1978).

**216. Tarsometatarsus, calcaneal ridges of hypotarsus, distal extension:** (0) short; (1) long. The calcaneal ridges of the hypotarsus extend further distally (i.e., proximodistally longer than lateromedially wide) in some species of alcids (e.g., *Synthliboramphus wumizusume*) while in others (e.g., *Ptychoramphus aleuticus*), the calcaneal ridges are shorter (i.e., proximodistally shorter than lateromedially wide).

**217. Tarsometatarsus, medial crest of hypotarsus:** (0) absent; (1) present. The medial crest of the hypotarsus in many charadriiforms (e.g., *Sterna maxima*) is a well-developed posteriorly projecting and distally extending structure. In all alcids (e.g., *Alca torda*) the medial hypotarsal crest is reduced (i.e., relatively the same size as other hypotarsal crests).

**218. Tarsometatarsus, anterior groove, conformation** (Chandler 1990b, character 79): (0) deep groove; (1) shallow groove. The anterior surface of the shaft of the tarsometatarsus is relatively flat in some alcids (e.g., *Alca torda*) and a deep groove in others (e.g., *Cerorhinca monocerata*).

**219. Tarsometatarsus, cross-sectional shape:** (0) square; (1) rectangular. The tarsometatarsus of many alcids at mid-shaft is much wider than it is deep (e.g., *Fratercula cirrhata*), while in other alcids (e.g., *Cepphus columba*) the tarsometatarsus is approximately as wide as it is deep.

**220. Tarsometatarsus, proximal vascular foramina, penetration of medial calcaneal ridge:** (0) absent; (1) present. There are two proximal vascular foramina in alcids. The medial foramina penetrates the medial calcaneal ridge all species (e.g., *Alca torda*) except (e.g., *Ptychoramphus aleuticus*) in which this foramina is positioned distal to the distal extent of the medial calcaneal ridge.

**221. Tarsometatarsus, trochlear proportions** (Strauch 1985, character 20): (0) fairly robust (i.e., “normal”) proportions for charadriiforms; (1) long and slender. In the Lari and most alcids (e.g., *Alca torda*) the proportions of the trochlea are similar. In some murrelets (e.g., *Synthliboramphus antiquus*) the trochleae are relatively long and somewhat compressed and give the tarsometatarsus a slender appearance (Storer 1945b in Strauch, 1985).

**222. Tarsometatarsus, trochlea II in lateral view, overlap of trochlea III** (Chandler 1990b, character 81): (0) trochlea 2 does not overlap trochlea 3; (1) trochlea 2 overlaps trochlea 3. The distal extension of the second trochlea of the tarsometatarsus relative to the third trochlea is variable in Alcidae. In most alcids (e.g., *Alca torda*) trochlea 2 partially overlaps trochlea 3, while in some alcids (e.g., *Synthliboramphus antiquus*) there is no overlap at all.

**223. Tarsometatarsus, length:** (0) tarsometatarsus longer than femur; (1) tarsometatarsus shorter than femur. The tarsometatarsi of a few alcids (e.g., *Synthliboramphus antiquus*) are longer than their femurs. Most alcids have femurs that are longer than their tarsometatarsi (e.g., *Alca torda*).

### Integument

**224. Maxillary rhamphotheca, color of tip** (modified from Chandler 1990b, character 95): (0) black or very dark brown; (1) red, orange or yellow; (2) white. In some alcids the tip of the beak varies in color from the rest of the rhamphothecum. The distal tip of the maxillary rhamphothecum varies in color from black or very dark brown (e.g., *Alca torda*), to shades of red, orange and yellow (e.g., *Aethia psittacula*), to white or light colored (e.g., *Aethia pusilla*).

**225. Maxillary rhamphotheca, color** (modified from Chandler 1990b, character 92): (0) primarily red, yellow or orange; (1) primarily black; (2) horn or grey. The maxillary rhamphothecum varies in color from black only (e.g., *Uria aalge*), to horn or grey colored

(e.g., *Synthliboramphus wumizusume*), to shades of red, yellow or orange (e.g., *Fratercula arctica*).

**226. Maxillary rhamphotheca, lateral surface ornamentation** (modified from Chandler 1990b, character 98): (0) smooth; (1) **vertically grooved**. The rhamphothecum of some alcids have dorsoventrally oriented grooves (e.g., *Alca torda*), although this feature is not present in all alcids (e.g., *Alle alle*).

**227. Maxillary rhamphotheca, horn at base of maxilla:** (0) absent; (1) present. A dorsally projecting horn is present on the base of the posterior maxillary rhamphotheca of *Cerorhinca monocerata* and *Aethia pusilla*. This feature is absent in all other alcids (e.g., *Alle alle*) and the nearest outgroups to Alcidae.

**228. Maxillary rhamphotheca, seasonal change** (Chu 1998, character 121): (0) absent; (1) present. The bill structure of most alcids (e.g., *Alle alle*) does not change once they reach adulthood, while the bills of some species (e.g., *Fratercula arctica*) undergo dramatic changes associated with the breeding season.

**229. Mouth tissue color** (modified from Chandler 1990b, character 93): (0) yellow; (1) red or orange; (2) white. The lining inside the mouth varies in color from shades of red (e.g., *Cepphus grylle*), orange (e.g., *Fratercula corniculata*) and yellow (e.g., *Alca torda*) to white (e.g., *Aethia psittacula*; Ridgway, 1919).

**230. External nares, orientation:** (0) laterally or dorsally directed oval or slit; (1) ventrally directed, medially oriented slit. Most alcids (e.g., *Cepphus grylle*) have oval shaped dorsally oriented nares. A few alcids (e.g., *Fratercula* arctica) have nares in the form of long, ventrally opening, medially oriented slits.

**231. Nostril feathering** (Strauch 1985, character 22; Chandler 1990b, character 89): (0) nostrils bare; (1) partially feathered; (2) fully feathered. The nostrils of the Lari, some alcids (e.g., *Cerorhinca monocerata*), and most other charadriiforms are bare. Some alcids (e.g., *Cepphus grylle*) have partially feathered nostrils, and others have completely feathered ones (e.g., *Alca torda*). It is hypothesized that increasing feathering represents progressively derived states. This character was first used by Brandt (1837) to classify the alcids (Strauch 1985). **Ordered**

**232. Eye color:** (0) darkly colored; (1) lightly colored. The eye color of most alcids (e.g., *Alca torda*) is brown, although a few alcids (e.g., *Aethia pygmaea*) have yellow or grey colored eyes.

**233. Eye scales** (Strauch 1985, character 24; Chandler 1990b, character 90): (0) absent; (1) present. The Lari and most other charadriiforms have no eye scales (e.g., *Aethia psittacula*); they are present in some puffins (e.g.,*Fratercula corniculata*; Strauch, 1985). These dermal structures, although they change color during the breeding season and are undoubtedly used for mating display purposes, are also present year round and even on nestlings. This suggests that these ‘horns’ may have another purpose, possibly hydrodynamic. The evolution of a hardened horn solely for mating purposes seems unlikely given that a simple mating display could be achieved via feather coloration.

**234. Plume in front of eye** (Chandler 1990b, character 86): (0) absent; (1) present. *Aethia pygmaea* is unique in possessing a plume that originates anterior to the eye. All other alcids lack this feature.

**235. Plume behind eye** (Chandler 1990b, character 87): (0) absent; (1) present. While most alcids (e.g., *Alca torda*) lack this feature, some alcids (e.g., *Aethia pusilla*) possess a plume that originates posterior to the eye.

**236. Plume on forehead** (Chandler 1990b, character 85): (0) absent; (1) present. Although lacking in most alcids (e.g., *Alle alle*), some species (e.g., *Aethia psittacula*) possess a head plume that originates on the forehead (in addition to plumes behind and in front of the eye).

**237. Head plumage** (Strauch 1985, character 23): (0) typical feathering; (1) velvety plumage. The head plumage of the Lari and most other charadriiforms consists of typical feathers (e.g., *Aethia psittacula*); in some alcids (e.g., *Alca torda*) the head plumage is distinctly velvety (Strauch 1985).

**238. Neck plumage** (Chandler 1990b, character 101): (0) not notched; (1) notched. The neck plumage of some alcids (e.g., *Uria lomvia*) is notched in anterior view.

**239. White tips on secondaries** (Strauch 1985, character 26; Chandler 1990b, character 88): (0) absent; (1) present. In the Lari the secondaries may be solid-colored or white-tipped. The condition in the Lari thus does not indicate the primitive state in the Alcidae. Since dark-tipped secondaries (e.g., *Aethia pusilla*) are found in three of the four major groups of alcids [“widespread” according to the principles of Kluge and Farris (1969)], white tips (e.g., *Uria aalge*) are hypothesized to be a derived state (Strauch 1985).

**240. White wing patch** (Chandler 1990b, character 105): (0) absent; (1) present. Although absent in most alcids (e.g., *Alle alle*), some alcids possess a white wing patch (e.g., *Cepphus grylle*).

**241. Number of primaries:** (0) eleven; (1) nine. All alcids (e.g., *Cepphus grylle*) and many other charadriiforms have 10 functional primary flight feathers and an eleventh reduced primary, while some charadriiforms (e.g., *Charadriius wilsonia*) have 9 primaries.

**242. Number of retrices** (Strauch 1985, character 27; Chandler 1990b, character 96): (0) ten; (1) twelve; (2) fourteen; (3) sixteen or more. The Lari have12 retrices. Alcid species may have 12 (e.g., *Alca torda*), 14 (e.g., *Cepphus columba*), 16, or 18 retrices (e.g., *Fratercula arctica*). The number appears to be constant within a species except for *Cerorhinca monocerata*, which may have 16 or 18. It is hypothesized that an increasing number of retrices represents increasingly derived states (Strauch 1985). Some other charadriiforms (e.g., *Anous tenuirostris*) only have ten retrices. **Ordered**

**243. Shape of retrices** (Strauch 1985, character 28; Chandler 1990b, character 97): (0) rounded at tips; (1) pointed at tips. The retrices of the Lari and most other charadriiforms (e.g., *Cepphus columba*) have rounded tips. In some auks (e.g., *Alca torda*)the retrices are distinctly pointed at the tips (Strauch, 1985).

**244. Winter plumage** (Chandler 1990b, character 83): (0) contrasting dark mantle and white underparts; (1) mantle and underparts dark. While some species of alcids (e.g., *Aethia pygmaea*) remain darkly colored above and below year-round, many alcids (e.g., *Alca torda*) display white underparts during the winter months.

**245. Juvenile plumage:** (0) resembles winter adults; (1) resembles summer adults. The juvenile plumage of all acids (e.g., *Uria lomvia*) except *Alca torda* and *Alle alle* resembles the winter plumage of adults, in which the juvenile plumage resembles the summer plumage of adults (Kozlova 1957).

**246. Moult:** (0) simultaneous; (1) gradual. Most alcids (e.g., *Uria lomvia*) moult their flight feathers simultaneously, resulting in a roughly 45-day period of flightlessness. Only the auklets (e.g., *Aethia pygmaea*) moult their flight feathers gradually, maintaining the ability of flight year-round (Kozlova 1957).

**247. Tail shape** (modified from Chandler 1990b, character 99): (0) rounded; (1) central notched; (2) pointed; (3) forked. Variation in the tail shape of alcids includes rounded (e.g., *Uria lomvia*), central notched (e.g., *Fratercula arctica*) and pointed (e.g., *Alca torda*).

**248. Foot color** (modified from Chandler 1990b, character 94): (0) red, orange, or pink; (1) white or grey; (2) black or dark brown; (3) buff or tan. Foot color in the Alcidae varies from red (e.g., *Cepphus columba*), to grey (e.g., *Aethia pygmaea*), to black (e.g., *Alca torda*; Ridgway 1919).

**249. Scutellation** (modified from Strauch 1985, character 29; Chandler 1990b, character 106): (0) scutellate; (1) reticulate. The scutellation on the dorsal podotheca (i.e., acrotarsium) of the Lari is scutellate. In alcids it may be either scutellate (e.g., *Alca torda*) or reticulate (e.g., *Aethia pygmaea*; Strauch 1985).

**250. Ungual (claw) of inner toe, shape** (modified from Strauch 1985, character 21): (0) gracile, gently curving; (1) stout and strongly recurved. The second claw of most charadriiforms (e.g., *Aethia pygmaea*) is moderately arched, compressed, and acute (Coues, 1868). In puffins that dig their own burrows (e.g., *Fratercula arctica*), the inner (second) toe is usually stout and strongly recurved (Strauch 1985).

**251. Foot webbing:** (0) absent; (1) present. All alcids (e.g., *Alca torda*) have webbing between the second, third and fourth toes. Some charadriiforms (e.g., *Charadriius wilsonia*) lack this characteristic.

**252. Hallux (**Chandler 1990b, character 80): (0) absent; (1) present. Alcids (e.g., *Uria lomvia*) differ from many other charadriiforms (e.g., *Larus marinus*) in that they lack a first toe or hallux.

**253. White face color in breeding plumage** (Chandler 1990b, character 91): (0) absent; (1) present. During the breeding season the face color of some alcids (e.g., *Fratercula arctica*) changes to white in color. The face color of most alcids (e.g., *Alca torda*) is not white during the reproductive phase.

**254. Belly color during breeding plumage** (Chandler 1990b, character 84): (0) white; (1) black; (2) mottled brown; (3) grey. The belly-color of alcids during the breeding season varies from white (e.g., *Alca torda*), to black (e.g., *Fratercula cirrhata*), to mottled brown (e.g., *Brachyramphus marmoratus*), to grey (e.g., *Aethia pygmaea*).

**255. Barred breeding plumage** (Chandler 1990b, character 104): (0) absent; (1) present. The breeding plumage of some alcids (e.g., *Brachyramphus marmoratus*) is barred.

### Reproduction

**256. Incubation patches** (Strauch 1985, character 25; Chandler 1990b, character 103): (0) two; (1) one. Paired lateral incubation patches are found in shorebirds, Lari, and some alcids (e.g., *Alle alle*; Bailey 1952). Some alcids (e.g., *Alca torda*) have only one patch (Strauch 1985).

**257. Nest sites** (modified from Strauch 1985, character 32): (0) bare rock or scrape; (1) natural crevices; (2) burrows; (3) built of sticks, grass, feathers, etc. The Lari nest in the open, as do some alcids (*Ptychoramphus aleuticus*). Other alcids (e.g., *Fratercula arctica*) nest in crevices or in burrows. Kozlova (1957) thought that the original nest sites of alcids were on open rocks or coastal cliffs (e.g., *Alca torda*). It is hypothesized that nesting in crevices or in burrows represents increasingly derived conditions (Strauch, 1985).

**258. Nesting dispersion** (Strauch 1985, character 33): (0) colonial; (1) solitary. The Lari and most of the alcids (e.g., *Alca torda*) nest in colonies. Some species of alcids (e.g., *Brachyramphus marmoratus*), however, nest solitarily (Strauch 1985).

**259. Nesting proximity to shore:** (0) near-shore; (1) inland. Although most alcids (e.g., *Alca torda*) nest on sea-cliffs or rocky beaches near-shore, a few alcids (e.g., *Brachyramphus marmoratus*) nest further inland.

**260. Clutch size** (Strauch 1985, character 30; Chandler 1990b, character 102): (0) one; (1) two or more. The Lari and almost all other charadriiforms lay a clutch of two or more. Although some alcids (e.g., *Cepphus grylle*) lay two eggs, most species lay only one (e.g., *Alca torda*; Strauch 1985).

**261. Egg shape** (modified from Chandler 1990b, character 100): (0) ovate; (1) pyriform; (2) elliptical; (3) sub-elliptical/ovate. Alcid eggs display considerable variety of shape. The eggs of the majority of alcid species (e.g., *Cepphus grylle*) are characterized as sub-elliptical/ovate in shape. The second most common alcid egg shape is ovate (e.g., *Alle alle*). Other shapes include pyriform (e.g., *Pinguinus impennis*) and elliptical (e.g., *Synthliboramphus hypoleucus*).

**262. Egg markings, scribbling:** (0) absent; (1) present. The eggs of some alcids (e.g., *Pinguinus impennis*) display complex ‘scribbles’, although the eggs of most alcids (e.g., *Aethia cristatella*) lack this feature.

**263. Egg texture**: (0) smooth; (1) granular. The eggs of some alcids (e.g., *Alca torda*) have a rough, granular texture. The eggs of other alcids (e.g., *Cepphus grylle*) and most charadriiforms have a smooth texture.

**264. Egg luster**: (0) non-glossy; (1) glossy. The luster of murrelet (e.g., *Synthliboramphus antiquus*) eggs varies from all other alcids (e.g., *Alca torda*) in having a glossy luster.

**265. Color of downy chicks:** (0) variable; (1) primarily brown; (2) primarily black; (3) primarily grey; (4) primarily buff or white. The down feathering of charadriiform chicks is predictably colored in most species (e.g., black in *Cepphus grylle*), although the color of the down feathers in some terns (e.g., *Sterna maxima*) is variable (i.e., sometimes black, sometimes buff).

**266. Post-hatching development pattern** (Strauch 1985, character 31): (0) semi-precocial; (1) intermediate; (2) precoccial. Alcids have three distinct post-hatching development patterns: precocial, intermediate, and semi-precocial (Sealy 1973). The pattern for *Pinguinus* is unknown. Bengtson (1984), in a review of the literature on *Pinguinus*, estimated that chicks leave the nest at about 10 days old, which would agree with an intermediate pattern. In the Lari the pattern is semi-precocial; it is hypothesized that shortening of the nestling period in alcids represents a derived condition (Strauch 1985). **Ordered**

### Diet

**267. Adult prey preference:** (0) primarily invertebrates; (1) primarily vertebrates; (2) significant amounts of invertebrates and vertebrates. Many of the smaller alcids (e.g., *Alle alle*) are specialized feeders on small invertebrates, while some larger alcids (i.e., *Fratercula arctica*) subsist on a diet of mostly fish (del Hoyo et al. 1996).

**268. Chick diet:** (0) primarily invertebrates; (1) primarily vertebrates; (2) significant amounts of invertebrates and vertebrates. The diet that alcids feed to their chicks varies from primarily invertebrates such as copepods, amphipods, and euphausiids (del Hoyo et al. 1996; e.g., *Alle alle*), to primarily vertebrates such as fish and eels (del Hoyo et al. 1996; e.g., *Uria lomvia*). Many close outgroup taxa to Alcidae are more generalized feeders (i.e., a combination of both invertebrates, vertebrates, carrion, trash; del Hoyo et al. 1996; e.g., *Larus marinus*).

### Musculature

(see Hudson et al. 1969 for complete character descriptions)

**269. M. pectoralis abdominalis insertion on** (Hudson et al. 1969): (0) tendon of M. pectoralis thoracica; (1) humerus.

**270. Anterior head of M. subcoracoideus** (Hudson et al. 1969): (0) small or absent; (1) short or long.

**271. M. propatagialis longus dilation at wrist** (Hudson et al. 1969): (0) unossified; (1) ossified.

**272. M. propatagialis** (Hudson et al. 1969): (0) two tendons; (1) one tendon.

**273. Patagial fan sesamoid** (Hudson et al. 1969) : (0) present; (1) absent.

**274. M. deltoideus minor dorsal head** (Hudson et al. 1969): (0) present; (1) absent.

**275. Swelling in M. triceps tendons** (Hudson et al. 1969): (0) unossified; (1) ossified.

**276. Swelling in humero-ulnar pulley** (Hudson et al. 1969): (0) ossified; (1) unossified.

**277. M. biceps brachii** (Hudson et al. 1969): (0) divided lengthwise; (1) divided distally; (2) undivided.

**278. M. flexor digitorum sublimis dilation at base of phalanx 1** (Hudson et al. 1969): (0) ossified; (1) unossified.

**279. M. ulnimetacarpalis dorsalis ventral head** (Hudson et al. 1969): (0) present; (1) absent.

**280. M. ambiens** (Hudson et al. 1969): (0) present; (1) absent.

**281. Pars iliofemoralis of M. piriformis** (Hudson et al. 1969): (0) absent; (1) present.

**282. Pars interna of M. gastrocnemius** (Hudson et al. 1969): (0) extends around anterior surface of knee; (1) does not extend around anterior surface of knee.

**283. Pars interna of M. gastrocnemius** (Hudson et al. 1969): (0) no extra head from tibia; (1) extra head from tibia.

**284. Pars medialis of M. gastrocnemius** (Hudson et al. 1969): (0) present; (1) absent.

**285. M. plantaris** (Hudson et al. 1969): (0) present; (1) absent.

**286. Sesamoid of M. scapulotriceps** (Hudson et al. 1969): (0) absent; (1) present.

**287. Sesamoid of M. humerootriceps** (Hudson et al. 1969): (0) absent; (1) present.

**288. Sesamoid of humero-ulnar pulley** (Hudson et al. 1969): (0) absent; (1) present.

**289. Sesamoid of propatagialis longus at wrist** (Hudson et al. 1969): (0) absent; (1) present.

**290. Sesamoid of flexor digitorum profundus in hand** (Hudson et al. 1969): (0) absent; (1) present.

**291. Sesamoid of flexor digitorum sublimis at base of phalanx 1** (Hudson et al. 1969): (0) absent; (1) present.

**292. Sesamoid of flexor digitorum longus** (Hudson et al. 1969): (0) absent; (1) present.

### Feather microstructure

(see Dove 2000 for complete character descriptions)

**293. Subpennaceous region** (modified from Dove 2000 character 1): (0) absent; (1) present.

**294. Subpennaceous region pigmentation** (modified from Dove 2000 character 2): (0) absent, both vanules unpigmented (1) present, both vanules pigmented.

**295. Subpennaceous region pigmentation position** (modified from Dove 2000 character 2): (0) distal vanule more pigmented; (1) both vanules equally pigmented**.**

**296. Subpennaceous length** (modified from Dove 2000 character 3): (0) short; (1) long; (2) very long.

**297. Barbule base pigmentation** (modified from Dove 2000 character 4): (0) absent; (1) present.

**298. Barbule base length** (modified from Dove 2000 character 5): (0) short; (1) long; (2) continuous with pennulum.

**299. Barbule base cells** (modified from Dove 2000 character 6): (0) not visible; (1) visible.

**300. Barbule base cell composition** (modified from Dove 2000 character 6): (0) single cell; (1) multiple cells; (2) both single and multiple.

**301. Barb length** (modified from Dove 2000 character 7): (0) short; (1) long; (2) both short and long.

**302. Barb pigmentataion** (modified from Dove 2000 character 8): (0) absent, not pigmented; (1) present, pigmented.

**303. Barb pigmentation position** (modified from Dove 2000 character 8): (0) pigmented base to tip; (1) proximally pigmented; (2) both fully pigmented and unpigmented; (3) both fully pigmented and half-pigmented.

**304. Barbule pigmentation** (modified from Dove 2000 character 11): (0) absent, no pigmented nodes; (1) present, all nodes pigmented.

**305. Barbule pigmentation position** (modified from Dove 2000 character 11): (0) proximal nodes pigmented; (1) all nodes pigmented.

**306. Node expansion** (modified from Dove 2000 character 12): (0) unexpanded; (1) expanded.

**307. Node expansion location** (modified from Dove 2000 character 12): (0) uniform; (1) proximal.

**308. Density of nodes per barbule** (modified from Dove 2000 character 13): (0) sparse; (1) dense.

**309. Proximal node shape** (modified from Dove 2000 character 14): (0) normal; (1) flared; (2) oblong; (3) straight.

**310. Midsection nodes shape** (modified from Dove 2000 character 15): (0) normal; (1) flared; (2) oblong; (3) straight.

**311. Distal nodes** (modified from Dove 2000 character 16): (0) indistinct, not visible; (1) distinct, visible.

**312. Distal node shape** (modified from Dove 2000 character 16): (0) normal; (1) oblong.

**313. Nodal spines** (modified from Dove 2000 character 17): **(**0) absent; (1) present.

**314. Nodal spine position** (modified from Dove 2000 character 17): (0) present at all nodes; (1) present at basal nodes; (3) with and without spines.

**315. Nodal prongs** (modified from Dove 2000 character 18): (0) absent; (1) present.

**316. Nodal points** (modified from Dove 2000 character 19): (0) absent; (1) present.

**317. Nodal point position** (modified from Dove 2000 character 19): (0) present at all nodes; (1) present at basal nodes; (2) present at distal nodes; (3) nodes with and without points.

**318. Proximal node pigment** (modified from Dove 2000 character 20): (0) absent or only a few pigment granules; (1) many pigment granules present.

**319. Proximal node pigment shape** (modified from Dove 2000 character 20): (0) long and constricted; (1) diamond shaped; (2) short and constricted; (3) round; (4) diffuse.

**320. Mid-node pigment** (modified from Dove 2000 character 21): (0) absent or only a few pigment granules; (1) many pigment granules present.

**321. Mid-node pigment shape** (modified from Dove 2000 character 21): (0) long and constricted; (1) diamond shaped; (2) short and constricted; (3) round; (4) diffuse.

**322. Distal node pigmentation** (modified from Dove 2000 character 22): (0) unpigmented nodes; (1) nodes pigmented.

**323. Distal pigment distribution** (modified from Dove 2000 character 22): (0) continuous pigmentation; (1) distal pigmentation; (2) trailing pigment; (3) node clear, internode pigmented.

**324. Nodal pigment intensity at basal nodes** (modified from Dove 2000 character 23): (0) absent; (1) present.

**325. Nodal pigment intensity at basal nodes** (modified from Dove 2000 character 23): (0) lightly pigmented; (1) heavily pigmented.

**326. Nodal pigment at distal nodes** (modified from Dove 2000 character 24): (0) absent; (1) present.

**327. Nodal pigment intensity at distal nodes** (modified from Dove 2000 character 24): (0) lightly pigmented; (2) heavily pigmented.

**328. Pigment color** (modified from Dove 2000 character 25): (0) brown; (1) black; (2) light reddish-brown.

**329. Morphology of first node** (modified from Dove 2000 character 26): (0) reduced; (1) similar to other nodes; (2) both reduced and expanded first nodes.

**330. Internode pigmentation** (modified from Dove 2000 character 27): (0) absent; (1) present.

**331. Internode pigmentation** (modified from Dove 2000 character 27): (0) stippled; (1) heavily pigmented; (2) uniformly pigmented.

**332. True down pigmentation** (modified from Dove 2000 character 30): (0) absent; (1) present.

**333. True down pigmentation** (modified from Dove 2000 character 30): (0) proximal; (1) present throughout.

**334. True down nodes** (modified from Dove 2000 character 31): (0) node indistinct, not visible (1) distinct, visible.

**335. True down nodes** (modified from Dove 2000 character 31): (0) flared; (1) normal; (2) both flared and normal.

**336. True down pigment shape** (modified from Dove 2000 character 32): (0) long and constricted; (1) diamond shaped; (2) short and constricted; (3) round; (4) diffuse.

**337. True down pigmented like contour down** (modified from Dove 2000 character 33): (0) no; (1) yes.

**338. True down pigmented like afterfeather down** (modified from Dove 2000 character 34): (0) no; (1) yes.

**339. Afterfeather pigmentation** (modified from Dove 2000 character 35): (0) absent; (1) present.

**340. Afterfeather pigmentation** (modified from Dove 2000 character 35): (0) proximal; (1) throughout; (2) distal.

**341. Afterfeather down pigmented like contour feather down** (modified from Dove 2000 character 36): (0) no; (1) yes.

**342. Villi** (modified from Dove 2000 character 37): (0) absent; (1) present.

**343. Distal prongs** (modified from Dove 2000 character 38): (0) absent; (1) present.

**344. Distal prong morphology** (modified from Dove 2000 character 38): (0) unequal length; (2) equal length.

### DNA sequence data

(see Appendix 5for Genbank accession numbers)

345. – 1487. cytb

1488. – 2528. ND2

2529. – 4343. ND5

4344. – 4865. ND6

4866. – 6416. CO1

6417. – 9287. RAG1

9288. – 10331. 12S

10332. – 11945. 16S

### Rejected characters

The following characters from the dataset of Chandler (1990) were rejected due to intraspecies variability: 15, 32, 48, 55, 72, 73, 77, 78; or because they were parsimony uninformative (i.e., they did not vary among taxa examined): 27, 31, 34, 45, 54, 56, 57, 66, 68, 76.

The following characters of Dove (2000) were not included the matrix because they did not vary in any taxa examined in this study: 9, 10, 28, 29. Several characters of Dove (2000) were split into two separate characters following the philosophy of character independence with respect to absence of character states outlined by Hawkins et al. (1997).

All the characters of Strauch (1985) and Chandler (1990) were rescored for this analysis using multiple specimens (see Appendix 1,comparative material). Many of the characters of Strauch (1985) and Chandler (1990) were modified to describe variability not originally noted by those authors (see notations in character list).

Although all characters from Hudson et al. (1969) were not rescored, scorings for *Uria aalge*, *Alle alle*, *Fratercula corniculata*, *Cepphus columba*, *Aethia psittacula*, and *Synthliboramphus antiquus* were confirmed through dissection of preserved specimens (see comparative material for specimen #’s).

## Appendix 4. Morphological character scorings

Morphological character scorings for all extant and extinct taxa, 18 Mancallinae specimen terminals (LACM, SDSNH, and USNM catalog numbers), and a Mancallinae supraspecific terminal (Mancallinae SST) are listed below. Polymorphic scorings are represented by capital letters (A:0&1; B:0&2; C:1&2).

**Table:**

								1	0									2	0									3	0									4	0									5	0									6	0									7	0			
*Aethia cristatella*	1	1	0	1	0	1	0	1	1	1	1	0	0	0	0	1	0	1	0	0	0	1	0	1	0	0	0	1	0	1	0	0	-	1	0	1	1	1	0	0	1	0	1	1	1	0	1	0	0	0	1	1	1	0	1	4	0	1	0	1	1	1	0	-	1	0	1	1	1	1	1	1	1	0	1
*Aethia pygmaea*	0	0	0	0	0	1	0	1	1	1	1	0	0	0	0	1	0	1	0	0	0	1	0	1	0	0	0	1	0	0	0	0	-	1	0	1	1	1	0	0	0	0	0	1	1	0	0	0	0	0	1	1	1	0	1	5	0	1	0	1	1	1	0	-	1	0	1	1	1	1	1	1	1	0	1
*Aethia psittacula*	0	1	0	1	0	1	0	1	1	1	1	0	0	0	0	1	0	1	0	0	0	1	0	1	0	0	0	1	0	1	0	0	-	1	0	0	1	1	1	0	0	0	0	1	1	0	1	0	0	0	1	1	1	0	1	5	0	1	0	1	2	1	0	-	1	0	1	1	1	1	1	1	1	0	1
*Aethia pusilla*	0	0	0	1	0	1	0	1	1	1	1	0	0	0	0	1	0	1	0	0	0	1	0	1	0	0	0	1	0	0	0	0	-	1	0	1	1	1	0	0	0	0	1	1	1	0	1	0	0	0	1	1	1	0	1	4	0	1	0	1	C	1	0	-	1	0	1	1	1	1	1	1	1	0	1
*Ptychoramphus aleuticus*	0	0	0	1	0	1	0	1	1	1	1	0	0	0	0	1	0	1	0	0	0	1	0	1	0	0	0	1	0	0	0	0	-	1	0	1	1	1	0	0	0	0	0	1	1	0	1	0	0	0	1	1	1	0	1	4	0	1	0	1	2	1	0	-	0	0	1	1	1	1	1	1	1	0	1
*Cerorhinca monocerata*	1	1	1	1	1	0	1	0	0	1	0	0	0	0	0	1	0	1	0	0	0	1	0	1	0	0	0	1	1	1	0	0	-	1	0	0	1	1	1	1	0	0	0	1	1	1	1	0	0	0	1	1	1	0	1	2	0	1	0	1	2	1	1	1	0	0	1	1	1	1	1	1	1	0	1
*Fratercula arctica*	1	1	1	1	1	1	1	0	0	1	0	0	0	0	0	1	0	1	0	0	0	1	0	1	0	0	0	1	1	1	0	0	-	0	0	0	1	1	1	1	1	0	0	1	1	1	1	0	0	1	1	1	1	1	1	2	0	1	0	1	2	1	1	1	0	0	1	1	1	1	1	1	1	0	1
*Fratercula corniculata*	1	1	1	1	1	1	1	0	0	1	0	0	0	0	0	1	0	1	0	0	0	1	0	1	0	0	0	1	1	1	0	0	-	0	0	0	1	1	1	1	1	0	0	1	1	1	1	0	0	1	1	1	1	1	1	3	0	1	0	1	1	1	1	1	0	0	1	1	1	1	1	1	1	0	1
*Fratercula cirrhata*	1	1	1	1	1	1	1	0	0	1	0	0	0	0	0	1	0	1	0	0	0	1	0	1	0	0	0	1	1	1	0	0	-	0	0	0	1	1	1	1	1	0	0	1	1	1	1	0	0	0	1	1	1	1	1	2	0	1	0	1	2	1	1	1	0	0	1	1	1	1	1	1	1	0	1
*Alca torda*	1	1	1	0	0	0	0	0	0	0	0	1	0	0	0	1	0	1	0	1	1	1	1	1	1	0	0	1	0	0	1	1	1	1	0	0	0	1	1	0	1	0	0	0	1	0	1	1	1	1	1	0	1	1	1	0	0	1	0	0	2	0	0	-	1	0	0	1	1	0	1	0	0	1	1
*Pinguinus impennis*	1	1	1	0	0	0	0	0	0	0	0	1	0	0	0	1	0	1	0	1	1	1	1	1	1	0	1	1	0	0	1	2	1	1	0	0	0	1	0	0	1	0	0	0	1	0	1	1	1	1	1	0	1	0	?	?	0	1	0	0	2	0	0	-	1	0	0	1	1	0	1	0	0	1	1
*Alle alle*	1	0	1	0	0	0	0	0	1	0	0	0	1	0	0	1	0	1	1	1	1	1	0	1	0	0	0	1	0	0	0	0	-	1	0	1	0	1	0	0	0	0	1	1	1	0	0	1	1	1	1	1	0	1	1	3	0	1	0	0	2	1	0	-	1	0	0	1	1	0	1	0	0	0	1
*Uria aalge*	0	0	1	0	0	0	0	0	1	0	0	1	0	1	0	1	0	1	0	1	1	1	1	1	0	0	0	1	0	0	1	1	A	0	0	0	1	1	0	0	0	0	1	1	0	0	1	1	1	1	0	0	0	1	0	0	1	0	0	2	0	0	-	1	0	0	1	1	0	1	0	0	1	1	1
*Uria lomvia*	0	0	1	0	0	0	0	0	1	0	0	1	0	0	0	1	0	1	0	1	1	1	1	1	0	0	0	1	0	0	1	1	0	0	0	0	0	1	1	0	0	0	0	1	1	0	0	1	1	1	1	0	0	0	1	0	0	1	0	0	2	0	0	-	1	0	0	1	1	0	1	0	0	1	1
*Cepphus carbo*	0	0	0	1	0	0	0	0	0	0	0	0	1	0	0	0	1	1	0	1	0	1	0	1	0	0	0	1	0	0	1	1	1	0	0	0	0	1	1	0	0	0	0	0	1	0	0	0	1	1	1	1	0	1	1	2	0	1	0	0	1	1	0	-	1	0	0	1	1	1	1	0	1	0	1
*Cepphus columba*	0	0	0	0	0	0	0	0	0	0	0	0	1	0	0	0	1	1	0	1	0	1	0	1	0	0	0	1	0	0	1	1	1	0	0	0	0	1	1	0	0	0	0	0	1	0	1	0	1	1	1	0	0	0	1	1	0	1	0	0	2	1	0	-	1	0	0	1	1	1	1	0	1	0	1
*Cepphus grylle*	0	0	0	0	0	0	0	0	0	0	0	0	1	0	0	0	1	1	0	1	0	1	0	1	0	0	0	1	0	0	1	1	1	0	0	0	0	1	0	0	0	0	0	0	1	0	1	0	1	1	1	0	0	0	1	1	0	1	0	0	2	1	0	-	1	0	0	1	1	1	1	0	1	0	1
*Brachyramphus brevirostris*	0	0	1	0	1	0	0	0	0	1	0	0	0	0	0	0	0	1	1	0	0	1	0	1	0	0	0	1	0	0	0	0	-	1	0	1	0	1	0	0	0	0	0	0	1	0	1	1	1	0	1	0	0	0	1	0	0	1	0	0	2	1	0	-	1	0	0	1	0	-	1	0	0	0	1
*Brachyramphus marmoratus*	0	0	1	0	1	0	0	0	0	1	0	0	0	0	0	0	0	1	1	0	0	1	0	1	0	0	0	1	0	0	0	0	-	1	0	1	0	1	0	0	0	0	0	0	1	0	1	1	1	0	1	0	0	0	1	0	0	1	0	0	2	1	0	-	1	0	0	1	0	-	1	0	0	0	1
*Brachyramphus perdix*	0	0	1	0	1	0	0	0	0	0	0	0	0	0	1	0	0	1	1	0	0	1	0	1	0	0	0	1	0	0	0	0	-	0	0	1	0	1	1	0	0	0	0	0	1	0	0	1	1	0	1	0	0	0	1	0	0	1	0	0	2	1	0	-	1	0	0	1	0	-	1	0	0	0	1
*Synthliboramphus antiquus*	0	0	1	0	1	0	0	0	0	1	0	0	1	1	1	1	0	1	0	0	0	1	0	1	0	0	0	1	0	0	0	0	-	1	0	0	0	1	1	0	0	0	0	0	1	0	0	1	1	0	1	1	0	1	1	1	0	1	0	0	2	1	0	-	1	0	0	1	1	0	1	0	1	0	1
*Synthliboramphus wumizusume*	0	0	0	1	1	0	0	0	0	0	0	0	?	?	1	0	0	1	0	1	0	1	0	1	0	0	0	1	0	0	1	0	-	1	0	0	0	1	0	0	0	0	0	0	1	0	0	1	1	0	1	0	0	0	1	2	0	1	0	0	2	1	0	-	1	0	0	1	0	-	1	0	1	0	1
*Synthliboramphus craveri*	0	0	0	1	1	0	0	0	0	0	0	0	0	0	1	0	0	1	0	1	0	1	0	1	0	0	0	1	0	0	1	0	-	1	0	0	0	1	1	0	0	0	0	0	1	0	1	1	1	0	1	0	0	0	1	2	0	1	0	0	2	1	0	-	1	0	0	1	0	-	1	0	1	0	1
*Synthliboramphus hypoleucus*	0	0	1	0	1	0	0	0	0	1	0	0	1	1	1	0	0	1	0	0	0	1	0	1	0	0	0	1	0	0	0	0	-	1	0	0	0	1	1	0	0	0	0	0	1	0	0	1	1	0	1	0	0	1	1	1	0	1	0	0	2	1	0	-	1	0	0	1	0	-	1	0	1	0	1
*Stercorarius longicaudus*	1	0	1	0	0	0	0	0	0	0	0	0	1	1	0	0	0	1	0	0	0	0	1	1	0	0	0	1	0	0	0	0	-	0	1	1	0	0	0	0	0	0	0	0	1	1	2	2	0	-	0	-	0	0	0	-	1	0	1	0	1	1	0	-	0	0	1	0	0	-	0	0	1	0	0
*Stercorarius skua*	1	0	1	0	0	0	1	0	0	0	0	0	0	0	0	0	0	1	1	1	0	0	1	1	0	0	0	1	0	0	0	0	-	0	1	0	0	0	0	0	0	0	0	0	1	1	2	2	0	0	0	-	1	0	0	-	1	0	1	0	1	1	0	-	0	0	1	0	0	-	0	0	1	0	0
*Anous tenuirostris*	0	0	0	0	0	0	0	0	0	0	0	0	1	0	1	0	0	0	0	0	0	1	1	1	0	1	0	0	0	1	1	1	0	0	1	0	0	0	1	0	0	1	0	1	1	0	1	2	0	0	0	-	0	0	0	-	1	0	1	0	1	1	1	1	0	0	0	0	0	-	0	0	1	0	0
*Chlidonias leucoptera*	0	0	0	0	0	0	1	0	0	1	0	1	1	1	0	1	0	0	-	0	0	1	1	1	0	1	0	0	0	0	1	0	-	0	1	1	1	0	1	0	0	1	0	0	1	0	1	2	0	0	0	-	0	0	0	-	1	0	0	0	1	1	1	0	0	0	0	0	0	-	0	0	1	0	0
*Sterna niloteca*	0	0	1	0	0	0	1	0	0	0	0	1	1	0	0	0	0	0	-	0	0	1	1	1	0	1	0	0	0	0	1	1	0	0	1	0	1	0	1	0	0	1	0	0	1	0	1	2	0	0	0	-	0	0	0	-	1	0	0	0	1	1	1	0	0	0	0	0	0	-	0	0	1	0	0
*Larosterna inca*	0	0	0	0	0	0	1	0	0	0	0	1	0	1	0	1	0	0	-	0	0	1	1	1	0	0	0	0	0	0	1	1	0	0	1	0	1	0	1	0	0	1	0	0	1	0	1	2	0	0	0	-	1	0	0	-	1	0	0	0	1	1	1	0	0	0	0	0	0	-	0	0	1	0	0
*Sterna maxima*	0	0	0	0	0	0	1	0	0	0	0	0	1	0	0	0	0	0	-	0	0	0	1	1	0	1	0	1	0	0	1	1	0	0	1	0	1	0	1	0	0	1	0	0	1	0	2	2	0	1	0	-	0	0	0	-	1	0	1	0	1	1	1	0	0	0	0	0	0	-	0	0	1	0	0
*Phaetusa simplex*	0	0	0	0	0	0	1	0	0	0	0	1	1	1	0	1	0	0	-	0	0	1	1	1	0	0	0	0	0	0	1	1	0	0	1	0	1	0	1	0	0	1	0	0	1	0	1	2	0	1	0	-	0	0	0	-	1	0	1	0	1	1	1	0	0	0	0	0	0	-	0	0	1	0	0
*Sternula superciliaris*	0	0	0	0	0	0	1	0	0	0	0	0	0	0	0	0	0	0	-	0	0	1	1	0	0	1	0	1	0	0	1	1	0	1	1	1	0	0	1	0	0	1	0	0	1	1	2	2	0	0	0	-	0	0	1	3	1	0	0	0	1	1	1	0	0	0	0	0	0	-	1	1	1	0	0
*Sterna anaethetus*	0	0	1	0	0	0	1	0	0	0	0	1	1	0	0	0	0	0	-	0	0	1	1	1	0	1	0	0	0	0	1	1	0	1	1	0	1	0	1	0	0	1	0	0	1	0	1	2	0	0	0	-	0	0	0	-	1	0	1	0	2	1	1	0	0	0	0	0	0	-	0	1	1	0	0
*Gygis alba*	0	0	0	0	0	0	1	0	0	0	0	0	1	0	0	0	0	0	-	0	0	1	1	1	0	1	0	0	0	0	0	0	-	0	1	1	1	0	1	0	0	1	0	1	0	-	0	2	0	0	0	-	0	1	0	-	1	0	0	0	1	1	1	0	0	0	0	0	0	-	0	0	1	0	0
*Rynchops niger*	0	0	0	0	0	0	1	0	0	0	0	0	0	0	0	0	0	0	-	0	0	1	1	1	1	0	1	1	0	1	1	1	1	0	1	0	1	0	1	0	0	1	0	0	1	0	2	2	0	0	0	-	0	1	0	-	1	0	1	0	1	1	1	0	0	0	0	0	0	-	0	0	1	0	0
*Creagrus furcatus*	1	0	0	0	0	0	1	0	0	0	0	1	0	1	1	0	0	1	0	1	0	0	1	1	0	1	0	0	0	0	1	1	0	0	1	0	1	0	0	0	0	0	0	0	1	0	1	2	0	0	0	-	0	0	0	-	1	0	1	0	1	1	1	0	0	0	0	0	1	0	0	0	1	0	0
*Rhodostethia rosea*	0	0	1	0	0	0	1	0	0	0	0	0	0	1	0	0	0	0	-	1	0	1	1	1	0	1	0	0	0	0	0	0	-	0	1	1	1	0	0	0	0	0	1	0	1	0	1	2	0	1	0	-	0	0	0	-	1	0	0	0	1	1	1	0	0	0	0	0	0	-	0	0	1	0	0
*Larus argentatus*	1	0	0	0	0	0	1	0	0	0	0	0	0	1	0	0	0	1	0	0	0	0	1	1	1	1	0	1	0	0	1	1	0	0	1	0	1	0	0	0	0	0	0	0	1	0	2	2	0	1	0	-	0	0	0	-	1	0	1	0	1	1	1	0	0	0	1	0	0	-	0	0	1	0	0
*Larus marinus*	1	0	0	0	0	0	1	0	0	0	0	0	0	1	0	0	0	1	0	0	0	0	1	1	1	1	0	1	0	0	1	1	0	0	1	0	1	0	0	0	0	0	0	0	1	0	2	2	0	1	0	-	0	0	0	-	1	0	1	0	1	1	1	0	0	0	1	0	0	-	0	0	1	0	0
*Pagophila eburnea*	0	0	1	1	0	0	1	0	0	0	0	1	0	0	1	0	0	0	-	1	0	0	1	1	0	1	0	0	0	0	1	1	0	0	1	1	0	0	0	0	0	0	0	1	0	1	2	0	1	0	-	0	0	0	-	1	0	0	0	2	1	1	0	0	0	0	0	0	-	0	0	1	0	0	0
*Xema sabini*	0	0	0	1	0	0	1	0	0	0	0	0	0	1	0	0	0	1	1	1	0	0	1	1	0	1	0	0	0	0	0	1	0	0	1	1	1	0	0	0	0	0	0	1	0	0	0	2	0	0	0	-	0	0	0	-	1	0	0	0	1	1	1	0	0	0	0	0	0	-	0	0	1	0	0
*Rissa tridactyla*	0	0	1	0	0	0	1	0	0	0	0	1	0	1	1	0	0	1	0	0	0	1	0	1	0	1	0	0	0	0	1	1	0	1	1	0	1	0	0	0	0	0	0	0	1	0	0	2	0	1	0	-	0	1	0	-	1	0	0	0	1	1	1	0	0	0	0	0	0	-	0	0	1	0	0
*Cusorius temmneckii*	0	0	1	0	0	0	0	0	0	0	0	0	1	0	1	0	0	0	-	0	0	1	1	1	0	0	0	0	0	0	0	0	-	0	1	1	1	0	0	0	0	0	0	1	1	0	1	2	0	0	1	0	0	1	0	-	1	1	1	0	1	1	1	0	0	?	0	0	0	-	1	0	1	0	0
*Glareola maldivarum*	0	0	1	0	0	0	0	0	0	0	0	0	1	0	0	1	0	0	-	0	0	0	1	1	1	0	0	1	0	1	0	1	?	0	1	0	1	0	0	0	0	0	1	1	1	0	1	2	0	0	1	0	0	0	0	-	1	0	1	0	0	1	1	0	0	?	0	0	0	-	0	0	0	0	0
*Stiltia isabella*	1	0	1	0	0	0	0	0	0	0	0	0	1	0	0	0	1	1	1	0	0	0	1	0	1	0	0	1	0	1	1	0	-	0	1	1	1	0	0	0	0	0	1	1	1	0	2	2	0	0	1	0	1	0	1	3	1	1	1	0	0	1	1	0	0	1	0	0	0	-	1	0	1	0	0
*Rhinoptilus chalcopterus*	0	0	1	0	0	0	1	0	0	0	0	0	1	0	0	0	0	1	0	0	0	1	1	1	1	0	0	1	0	0	0	0	-	0	1	1	1	0	0	0	0	0	1	1	1	0	1	2	0	0	0	-	0	0	1	4	1	0	1	0	0	1	1	0	0	?	0	0	0	-	1	1	0	0	0
*Bartramia longicauda*	0	0	0	0	0	0	1	0	0	0	0	0	1	1	1	1	0	0	-	0	0	1	1	1	0	0	0	0	0	0	0	0	-	0	1	1	0	0	1	0	0	1	0	1	1	0	1	2	0	0	1	0	0	0	0	-	1	0	1	0	1	1	1	1	1	1	0	1	0	-	1	0	1	0	0
*Numenius minutus*	0	0	0	0	0	0	0	0	0	0	0	0	0	0	0	1	0	0	-	0	0	1	0	1	1	0	0	1	0	0	0	0	-	1	1	1	0	0	1	0	0	0	0	1	1	1	1	2	0	0	1	0	0	0	1	4	1	0	1	0	1	1	1	0	0	?	0	1	0	-	1	0	0	0	0
*Tryngites subruficollis*	0	0	0	0	0	0	1	0	0	0	0	1	0	1	0	0	0	0	-	0	0	1	1	1	0	0	0	0	0	0	0	0	-	0	1	1	0	0	1	0	0	0	0	0	1	0	1	2	0	0	0	-	0	0	0	-	1	1	0	0	1	1	0	-	0	0	0	0	0	-	1	0	1	0	0
*Hydrophasianus chirurgus*	0	0	0	0	0	0	1	0	0	0	0	0	0	0	1	0	0	0	-	0	0	1	0	0	0	1	0	0	0	0	0	0	-	0	1	1	1	0	0	0	0	0	0	0	1	0	1	1	0	0	1	0	0	0	0	-	1	0	0	0	0	1	0	-	0	1	0	0	0	-	1	1	1	0	0
*Charadrius vociferus*	0	0	1	0	0	0	1	0	0	0	0	0	0	1	0	0	0	1	1	0	0	1	1	0	0	0	0	0	0	0	0	0	-	1	1	1	1	0	0	0	0	0	0	0	0	-	1	1	0	-	1	0	0	0	0	-	1	1	0	0	1	1	1	1	0	0	0	0	0	-	1	1	0	0	0
*Charadrius wilsonia*	0	0	1	0	0	0	1	0	0	0	0	0	0	1	0	0	0	1	0	0	0	1	1	0	0	0	0	0	0	0	0	0	-	1	1	1	1	0	1	0	0	0	0	0	0	-	1	1	0	-	1	0	0	0	0	-	1	1	0	0	1	1	1	0	0	0	0	0	0	-	1	0	0	0	0
LACM 15410	?	?	?	?	?	?	?	?	?	?	?	?	?	?	?	?	?	?	?	?	?	?	?	?	?	?	?	?	?	?	?	?	?	?	?	?	?	?	?	?	?	?	?	?	?	?	?	?	?	?	?	?	?	?	?	?	?	?	?	?	?	?	?	?	?	?	?	?	?	?	?	?	?	?	?
LACM 128870	?	?	?	?	?	?	?	?	?	?	?	?	?	?	?	?	?	?	?	?	?	?	?	?	?	?	?	?	?	?	?	?	?	?	?	?	?	?	?	?	?	?	?	?	?	?	?	?	?	?	?	?	?	?	?	?	?	?	?	?	?	?	?	?	?	?	?	?	?	?	?	?	?	?	?
LACM 15425	?	?	?	?	?	?	?	?	?	?	?	?	?	?	?	?	?	?	?	?	?	?	?	?	?	?	?	?	?	?	?	?	?	?	?	?	?	?	?	?	?	?	?	?	?	?	?	?	?	?	?	?	?	?	?	?	?	?	?	?	?	?	?	?	?	?	?	?	?	?	?	?	?	?	?
LACM 23739	?	?	?	?	?	?	?	?	?	?	?	?	?	?	?	?	?	?	?	?	?	?	?	?	?	?	?	?	?	?	?	?	?	?	?	?	?	?	?	?	?	?	?	?	?	?	?	?	?	?	?	?	?	?	?	?	?	?	?	?	?	?	?	?	?	?	?	?	?	?	?	?	?	?	?
USNM 243765	?	?	?	?	?	?	?	?	?	?	?	?	?	?	?	?	?	?	?	?	?	?	?	?	?	?	?	?	?	?	?	?	?	?	?	?	?	?	?	?	?	?	?	?	?	?	?	?	?	?	?	?	?	?	?	?	?	?	?	?	?	?	?	?	?	?	?	?	?	?	?	?	?	?	?
LACM 107028	?	?	?	?	?	?	?	?	?	?	?	?	?	?	?	?	?	?	?	?	?	?	?	?	?	?	?	?	?	?	?	?	?	?	?	?	?	?	?	?	?	?	?	?	?	?	?	?	?	?	?	?	?	?	?	?	?	?	?	?	?	?	?	?	?	?	?	?	?	?	?	?	?	?	?
SDSNH 24262	?	?	?	?	?	?	?	?	?	?	?	?	?	?	?	?	?	?	?	?	?	?	?	?	?	?	?	?	?	?	?	?	?	?	?	?	?	?	?	?	?	?	?	?	?	?	?	?	?	?	?	?	?	?	?	?	?	1	0	1	2	1	?	?	0	1	0	0	0	-	1	?	?	?	?
SDSNH 59047	?	?	?	?	?	?	?	?	?	?	?	?	?	?	?	?	?	?	?	?	?	?	?	?	?	?	?	?	?	?	?	?	?	?	?	?	?	?	?	?	?	?	?	?	?	?	?	?	?	?	?	?	?	?	?	?	?	?	?	?	?	?	?	?	?	?	?	?	?	?	?	?	?	?	?
SDSNH 28152	?	?	?	?	?	?	?	?	?	?	?	?	?	?	?	?	?	?	?	?	?	?	?	?	?	?	?	?	?	?	?	?	?	?	?	?	?	?	?	?	?	?	?	?	?	?	?	?	?	?	?	?	?	?	?	?	?	?	?	?	?	?	?	?	?	?	?	?	?	?	?	?	?	?	?
SDSNH 42532	?	?	?	?	?	?	?	?	?	?	?	?	?	?	?	?	?	?	?	?	?	?	?	?	?	?	?	?	?	?	?	?	?	?	?	?	?	?	?	?	?	?	?	?	?	?	?	?	?	?	?	?	?	?	?	?	?	?	?	?	?	?	?	?	?	?	?	?	?	?	?	?	?	?	?
SDSNH 21295	?	?	?	?	?	?	?	?	?	?	?	?	?	?	?	?	?	?	?	?	?	?	?	?	?	?	?	?	?	?	?	?	?	?	?	?	?	?	?	?	?	?	?	?	?	?	?	?	?	?	?	?	?	?	?	?	?	0	0	1	2	1	?	?	?	?	?	?	?	?	?	0	0	0	1
LACM 103940	?	0	?	?	1	0	?	?	?	?	?	?	?	?	?	?	?	?	?	?	1	?	?	?	?	?	?	?	?	?	1	1	0	?	?	?	?	?	?	?	0	0	?	1	1	0	1	2	0	1	1	0	?	?	?	?	?	?	?	?	?	?	?	?	?	?	?	?	?	?	?	?	?	?	?
SDSNH 25236	0	0	1	0	1	0	?	1	?	0	0	1	?	?	?	1	0	0	0	0	1	1	1	1	?	0	0	1	?	0	1	1	0	0	0	0	1	1	0	0	0	0	0	1	1	1	1	2	0	1	1	0	?	?	?	?	?	?	?	?	?	?	?	?	?	?	?	?	?	?	?	?	?	?	?
*Mancalla californiensis*	?	?	?	?	?	?	?	?	?	?	?	?	?	?	?	?	?	?	?	?	?	?	?	?	?	?	?	?	?	?	?	?	?	?	?	?	?	?	?	?	?	?	?	?	?	?	?	?	?	?	?	?	?	?	?	?	?	?	?	?	?	?	?	?	?	?	?	?	?	?	?	?	?	?	?
*Mancalla cedrosensis*	?	?	?	?	?	?	?	?	?	?	?	?	?	?	?	?	?	?	?	?	?	?	?	?	?	?	?	?	?	?	?	?	?	?	?	?	?	?	?	?	?	?	?	?	?	?	?	?	?	?	?	?	?	?	?	?	?	?	?	?	?	?	?	?	?	?	?	?	?	?	?	?	?	?	?
*Mancalla lucasi*	?	?	?	?	?	?	?	?	?	?	?	?	?	?	?	?	?	?	?	?	?	?	?	?	?	?	?	?	?	?	?	?	?	?	?	?	?	?	?	?	?	?	?	?	?	?	?	?	?	?	?	?	?	?	?	?	?	0	0	1	?	?	?	?	?	?	?	?	?	?	?	?	?	?	?
*Mancalla vegrandis*	?	?	?	?	?	?	?	?	?	?	?	?	?	?	?	?	?	?	?	?	?	?	?	?	?	?	?	?	?	?	?	?	?	?	?	?	?	?	?	?	?	?	?	?	?	?	?	?	?	?	?	?	?	?	?	?	?	?	?	?	?	1	0	-	0	1	0	0	?	?	?	?	0	0	1
*Miomancalla howardi*	0	1	?	?	?	0	?	?	?	?	?	?	?	?	?	?	?	?	?	0	1	?	?	?	?	?	?	?	?	?	1	1	?	?	?	?	?	?	1	0	1	?	?	?	1	1	?	?	?	?	?	?	?	?	?	?	?	?	?	?	?	?	?	?	?	?	?	?	?	?	?	?	?	?	?
*Miomancalla wetmorei*	?	?	?	?	?	?	?	?	?	?	?	?	?	?	?	?	?	?	?	?	?	?	?	?	?	?	?	?	?	?	?	?	?	?	?	?	?	?	?	?	?	?	?	?	?	?	?	?	?	?	?	?	?	?	?	?	?	?	?	?	?	?	?	?	?	?	?	?	?	?	?	?	?	?	?
Mancallinae SST	0	A	1	0	1	0	?	1	?	0	0	1	?	?	?	1	0	0	0	0	1	1	1	1	?	0	0	1	?	0	1	1	0	0	0	0	1	1	1	0	A	0	0	1	1	A	1	2	0	1	1	0	?	?	?	?	?	A	0	1	2	1	0	-	0	1	0	0	0	-	1	0	0	0	1
			8	0									9	0								1	0	0								1	1	0								1	2	0								1	3	0								1	4	0								1	5	0
*Aethia cristatella*	0	1	1	1	0	0	1	1	0	2	0	-	0	0	0	-	0	0	1	0	1	1	0	1	1	3	1	1	0	1	1	1	0	0	0	0	1	2	0	1	0	0	1	1	0	-	-	-	0	1	2	0	-	1	0	0	0	0	0	1	0	0	0	0	0	2	1	1	0	0	0	0	0	1	0
*Aethia pygmaea*	0	1	1	1	0	0	1	1	0	2	0	-	0	0	0	-	0	0	1	0	1	1	0	1	1	3	1	0	0	1	1	1	1	0	0	1	1	2	0	0	0	0	1	1	0	-	-	-	1	0	1	0	-	1	0	0	0	0	0	1	0	0	0	0	0	2	1	1	0	1	0	0	0	1	0
*Aethia psittacula*	0	1	1	1	0	0	1	1	0	2	0	-	0	0	0	-	0	0	1	0	1	1	0	1	1	3	1	1	0	1	1	1	0	0	0	0	1	2	0	1	0	0	1	1	0	-	-	-	0	1	2	0	-	1	0	0	0	0	0	1	0	0	0	0	0	2	1	1	0	0	0	0	0	1	0
*Aethia pusilla*	0	1	1	1	0	0	1	1	0	2	0	-	0	0	0	-	0	0	1	0	1	1	0	1	1	3	1	1	0	1	1	1	1	0	0	0	1	2	0	1	0	0	1	1	0	-	-	-	0	0	2	0	-	1	0	0	0	0	0	1	0	0	0	0	0	2	1	1	0	0	0	0	0	1	0
*Ptychoramphus aleuticus*	0	1	1	1	0	0	1	1	0	2	0	-	0	0	0	-	0	0	1	0	1	1	0	0	1	3	1	1	0	1	1	1	1	0	0	0	1	2	0	1	0	0	1	1	0	-	-	-	0	0	2	0	-	1	0	0	0	0	0	1	0	0	0	0	0	2	1	1	0	0	0	0	0	1	0
*Cerorhinca monocerata*	1	1	1	1	0	1	1	1	1	2	1	0	0	0	1	0	1	0	0	1	1	1	0	1	0	3	1	0	0	1	1	1	1	0	1	0	0	1	1	1	0	1	2	1	0	-	-	-	1	1	2	1	1	0	0	0	1	0	0	0	0	0	0	0	0	2	0	2	0	1	0	0	0	1	0
*Fratercula arctica*	1	1	1	1	0	1	1	1	1	2	1	0	0	0	1	0	1	0	0	1	1	1	0	1	1	3	1	0	0	1	1	1	1	0	1	0	0	1	0	0	0	1	1	1	0	-	-	-	1	1	2	1	1	0	0	0	0	0	0	0	1	0	0	0	0	2	0	2	0	1	0	0	0	1	0
*Fratercula corniculata*	1	1	1	1	0	1	1	1	1	2	1	0	0	0	1	0	1	0	0	1	1	1	0	1	1	3	1	0	0	1	1	1	1	0	1	0	0	1	0	1	0	0	1	1	0	-	-	-	1	1	2	1	1	0	0	0	0	0	0	0	1	0	0	0	0	2	0	2	0	1	0	0	0	1	0
*Fratercula cirrhata*	1	1	1	1	0	1	1	1	1	2	1	0	0	0	1	0	1	0	0	1	1	1	0	1	1	3	1	0	0	1	1	1	1	0	1	0	0	1	0	0	1	0	1	1	0	-	-	-	1	1	2	1	1	0	0	0	0	0	0	0	1	0	0	0	0	2	0	2	0	1	0	0	0	1	0
*Alca torda*	1	1	0	1	0	0	1	0	0	2	1	1	1	0	1	0	1	0	0	1	1	0	0	0	1	2	0	1	0	0	1	2	0	0	1	1	1	2	0	1	0	0	2	0	0	-	-	-	1	0	1	0	-	1	0	1	0	0	0	0	1	0	0	0	0	2	0	1	0	0	1	1	0	1	0
*Pinguinus impennis*	1	1	0	1	1	1	1	0	1	1	1	0	0	0	1	0	1	0	0	1	1	0	0	0	1	2	0	1	1	0	1	2	1	0	1	0	1	2	0	0	0	0	2	0	0	-	-	-	1	0	1	0	-	1	0	1	0	0	1	0	1	0	0	0	0	2	0	3	0	0	1	1	0	1	0
*Alle alle*	1	1	0	1	0	1	1	1	0	2	1	0	1	0	0	-	1	0	0	1	1	0	0	0	1	3	1	1	0	1	1	2	1	1	0	1	1	2	0	1	0	0	1	1	0	-	-	-	0	0	1	0	-	1	0	1	0	0	0	0	1	0	0	0	0	2	1	1	0	0	0	2	0	0	0
*Uria aalge*	1	1	0	1	0	1	1	0	1	1	1	0	1	0	1	0	1	0	0	0	1	0	1	0	1	2	1	0	0	0	1	2	0	0	0	0	1	2	0	0	0	0	1	1	0	-	-	-	1	0	1	0	-	1	0	1	0	0	0	0	1	0	0	0	0	2	0	3	0	0	1	0	0	1	0
*Uria lomvia*	1	1	0	1	0	1	1	0	1	1	1	0	1	0	1	0	1	0	0	0	1	0	1	0	1	2	1	0	0	0	1	2	0	0	0	0	1	2	0	0	0	0	1	1	0	-	-	-	1	0	1	0	-	1	0	1	0	0	0	0	1	0	0	0	0	2	0	3	0	0	1	0	0	1	0
*Cepphus carbo*	1	1	0	1	0	0	1	1	0	2	1	0	1	1	1	1	1	0	0	1	1	1	0	0	1	1	0	0	0	1	1	1	0	1	0	1	1	1	0	1	0	0	1	1	0	-	-	-	0	1	1	1	1	0	1	1	0	0	0	1	1	0	0	0	0	1	1	3	0	0	1	1	0	1	0
*Cepphus columba*	1	1	0	1	0	0	1	0	0	2	1	0	1	1	1	1	1	0	0	1	1	1	0	0	1	1	1	0	0	1	1	1	0	1	0	1	1	1	1	1	0	0	1	1	0	-	-	-	0	1	1	1	1	0	0	1	0	1	0	1	1	0	0	0	0	1	1	3	0	0	1	0	0	1	0
*Cepphus grylle*	1	1	0	1	0	0	1	0	1	1	1	0	1	1	1	1	1	0	0	1	1	1	0	0	1	1	1	0	0	1	1	1	0	1	0	1	1	1	1	1	0	0	1	1	0	-	-	-	0	1	1	1	1	0	0	1	0	1	0	1	1	0	0	0	0	1	1	3	0	0	1	0	0	1	0
*Brachyramphus brevirostris*	1	1	0	1	0	1	1	1	0	1	1	0	1	0	0	-	0	1	0	0	1	0	0	0	0	2	0	1	0	0	1	2	0	0	0	0	1	2	0	1	0	0	1	1	0	-	-	-	1	0	1	0	-	0	1	0	0	1	0	0	1	0	0	0	0	2	0	1	1	0	1	0	0	1	0
*Brachyramphus marmoratus*	1	1	0	1	0	1	1	1	0	1	1	0	1	0	0	-	0	1	0	0	1	0	0	0	0	2	1	1	0	0	1	2	0	0	0	0	1	2	0	0	0	0	1	1	0	-	-	-	1	0	1	0	-	0	1	0	0	1	0	0	1	0	0	0	0	2	0	1	1	0	1	0	0	1	0
*Brachyramphus perdix*	1	1	0	1	0	1	1	1	0	1	1	0	1	0	0	-	0	1	0	0	1	0	0	0	0	2	0	1	0	0	1	2	0	0	0	0	1	2	0	1	0	0	1	1	0	-	-	-	1	0	1	0	-	0	1	0	0	1	0	0	1	0	0	0	0	2	0	1	1	0	1	0	0	1	0
*Synthliboramphus antiquus*	1	1	0	1	0	0	1	0	0	2	1	0	0	0	1	0	1	0	0	1	1	1	0	0	0	1	1	1	0	1	1	2	0	0	0	0	1	1	0	1	0	0	1	1	0	-	-	-	0	0	1	0	-	1	0	1	0	0	0	1	1	0	0	0	0	2	0	3	1	0	1	0	0	1	0
*Synthliboramphus wumizusume*	1	1	0	1	0	1	1	0	0	2	1	0	0	0	1	0	1	0	0	1	1	1	0	0	0	1	0	1	0	0	1	2	0	0	0	1	1	2	0	0	0	0	1	1	0	-	-	-	1	0	2	0	-	0	0	0	0	0	0	1	1	0	0	0	0	2	0	3	1	0	1	1	0	1	0
*Synthliboramphus craveri*	1	1	0	1	0	1	1	0	0	2	1	0	0	0	1	0	1	0	0	1	1	1	0	0	1	1	0	1	0	0	1	2	0	0	0	0	1	2	0	0	0	0	1	1	0	-	-	-	1	0	2	0	-	0	0	0	0	0	0	0	1	0	0	0	0	2	0	3	1	0	1	1	0	1	0
*Synthliboramphus hypoleucus*	1	1	0	1	0	0	1	0	0	2	1	0	0	0	1	0	1	0	0	1	1	1	0	0	1	1	1	1	0	1	1	2	0	0	0	1	1	1	0	1	0	0	1	1	0	-	-	-	0	0	2	0	-	1	0	1	0	0	0	1	1	0	0	0	0	2	0	3	1	0	1	0	0	1	0
*Stercorarius longicaudus*	0	0	1	1	0	0	1	1	0	0	1	1	1	1	1	1	0	0	2	0	0	-	0	0	0	1	0	0	0	1	0	0	0	1	1	1	1	0	2	0	0	0	0	1	0	-	-	-	1	1	0	0	-	0	0	1	0	0	0	0	0	0	0	0	0	0	0	0	0	0	0	0	0	0	0
*Stercorarius skua*	0	0	1	1	0	0	0	0	0	0	1	1	0	0	1	0	0	0	2	0	0	-	0	0	0	1	0	0	0	1	0	0	0	0	1	1	1	0	2	0	0	0	0	1	0	-	-	-	1	1	0	0	-	0	0	1	0	0	0	0	1	0	0	0	0	0	0	0	0	0	0	0	0	0	0
*Anous tenuirostris*	0	0	1	1	0	0	1	0	0	0	1	0	0	0	1	1	0	0	2	0	0	-	0	0	0	2	0	0	0	1	0	0	0	0	0	1	1	0	2	0	0	0	0	1	0	-	-	-	0	1	0	0	-	0	1	1	0	0	0	0	0	0	0	0	0	0	0	0	0	0	0	0	0	0	0
*Chlidonias leucoptera*	0	0	0	0	0	0	0	1	0	0	1	0	0	1	1	1	0	0	2	0	0	-	0	0	0	2	0	0	0	1	0	0	0	0	0	1	1	0	2	0	0	0	0	1	0	-	-	-	0	1	1	1	0	0	0	1	0	0	0	1	0	0	0	0	0	0	1	0	1	0	0	0	0	0	0
*Sterna niloteca*	0	0	0	1	0	0	1	1	0	0	1	1	0	1	1	0	0	0	1	0	0	-	0	0	0	1	0	0	0	1	0	0	0	0	1	0	1	0	2	0	0	0	0	1	0	-	-	-	0	1	0	1	0	0	0	1	0	0	0	1	1	0	0	0	0	0	1	0	1	0	0	0	0	0	0
*Larosterna inca*	0	0	0	0	0	0	1	1	0	0	1	1	0	1	1	1	0	0	2	0	1	1	0	0	0	2	0	0	0	1	0	0	0	0	0	1	1	0	2	0	0	0	0	1	0	-	-	-	0	1	0	1	0	0	0	1	0	0	0	1	0	0	0	0	0	0	1	0	1	0	0	0	0	0	0
*Sterna maxima*	0	0	0	1	0	0	0	1	0	0	1	1	1	1	1	1	0	0	2	0	0	-	0	0	0	1	0	0	0	1	0	0	0	0	1	1	1	0	2	0	0	0	0	1	0	-	-	-	0	1	0	1	0	0	0	1	0	0	0	0	0	0	0	0	0	0	1	0	1	0	0	0	0	0	0
*Phaetusa simplex*	0	0	1	0	0	0	1	1	0	0	1	1	0	1	1	1	0	0	2	0	1	1	0	0	1	2	0	0	0	1	0	0	0	0	0	1	1	0	2	0	0	0	0	1	0	-	-	-	0	1	1	1	0	0	0	1	1	0	0	1	1	0	0	0	0	0	1	0	1	0	0	0	0	0	0
*Sternula superciliaris*	0	0	0	1	0	0	0	0	0	?	1	1	0	1	1	1	1	0	2	0	0	-	0	0	0	1	0	0	0	1	0	1	0	?	?	1	1	0	2	0	0	0	0	?	0	-	-	-	0	1	1	1	0	0	?	1	0	?	0	0	0	0	?	0	0	0	?	0	1	0	0	0	0	0	?
*Sterna anaethetus*	0	0	0	1	0	0	1	1	0	0	1	1	0	1	1	0	0	0	1	0	0	-	0	0	0	2	0	0	0	1	0	0	0	0	1	0	1	0	2	0	0	0	0	1	0	-	-	-	0	1	0	1	1	0	0	1	0	0	0	1	1	0	0	0	0	0	1	0	1	0	0	0	0	0	0
*Gygis alba*	0	0	1	0	0	0	1	1	0	0	1	1	0	1	1	0	0	0	1	0	1	0	0	0	0	1	0	0	0	1	0	0	0	1	0	0	1	0	2	0	0	0	0	1	0	-	-	-	0	1	1	1	0	0	0	1	0	0	0	1	1	0	0	0	0	0	1	0	0	0	0	0	0	0	0
*Rynchops niger*	0	0	0	1	0	0	0	1	0	0	1	1	1	1	1	1	0	0	2	0	0	-	0	0	0	1	0	1	0	1	0	0	0	1	0	1	1	0	2	0	0	0	0	1	0	-	-	-	0	1	0	1	1	0	0	1	0	0	0	1	0	0	0	0	0	0	0	0	0	0	0	0	0	1	0
*Creagrus furcatus*	0	0	1	0	0	1	0	1	0	0	1	1	0	1	1	1	0	0	2	0	0	-	0	0	1	1	0	0	0	1	0	0	0	1	1	1	1	0	2	0	0	0	0	1	0	-	-	-	0	1	0	1	0	0	0	1	0	0	0	0	1	0	0	0	0	0	0	0	0	0	0	0	0	0	0
*Rhodostethia rosea*	0	0	1	0	0	1	0	0	0	1	1	1	0	1	1	1	0	0	2	0	0	-	0	0	0	1	0	0	0	1	0	0	0	1	0	1	1	0	2	0	0	0	0	1	0	-	-	-	0	1	0	1	1	0	0	1	0	0	0	1	0	0	0	0	0	0	0	0	0	0	0	0	0	0	0
*Larus argentatus*	0	0	0	0	0	0	0	1	0	0	1	1	0	1	1	1	0	0	2	0	1	1	0	0	0	2	0	0	0	1	0	0	0	0	0	0	1	0	2	0	0	0	0	1	0	-	-	-	0	1	0	1	0	0	0	1	0	0	0	1	0	0	0	0	0	0	0	0	0	0	0	0	0	0	0
*Larus marinus*	0	0	0	0	0	0	0	1	0	0	1	1	0	1	1	1	0	0	2	0	1	1	0	0	0	2	0	0	0	1	0	0	0	0	0	0	1	0	2	0	0	0	0	1	0	-	-	-	0	1	0	1	0	0	0	1	0	0	0	0	0	0	0	0	0	0	0	0	0	0	0	0	0	0	0
*Pagophila eburnea*	0	0	1	0	0	0	1	0	0	0	1	1	0	1	1	0	0	0	1	1	1	1	0	0	1	1	0	0	0	1	0	0	0	0	0	0	1	0	2	0	0	0	0	1	0	-	-	-	0	1	1	1	0	0	0	1	0	0	0	1	1	0	0	0	0	0	0	0	0	0	0	0	0	0	0
*Xema sabini*	0	0	1	0	0	0	1	1	0	0	1	1	0	1	0	-	1	0	1	1	0	-	0	0	0	1	0	0	0	1	0	0	0	0	0	0	1	0	2	0	0	0	0	1	0	-	-	-	0	1	0	1	0	0	0	1	0	0	0	1	1	0	0	0	0	0	0	0	0	0	0	0	0	0	0
*Rissa tridactyla*	0	0	1	0	0	0	1	0	0	0	1	1	0	1	1	0	0	0	1	0	0	-	0	0	0	1	0	0	0	1	0	0	0	0	0	0	1	0	2	0	0	0	0	1	0	-	-	-	0	1	0	1	0	0	0	1	0	0	0	1	1	0	0	0	0	0	0	0	0	0	0	0	0	0	0
*Cusorius temmneckii*	0	0	0	1	0	0	0	0	0	?	?	?	0	1	0	-	0	0	2	0	0	-	0	0	?	1	0	0	0	1	?	2	0	?	?	0	1	0	2	1	0	0	1	?	0	-	-	-	0	0	0	0	-	0	?	0	0	?	0	0	1	0	?	0	0	0	?	0	0	0	0	0	0	0	?
*Glareola maldivarum*	0	0	0	1	0	1	1	0	0	?	?	?	0	1	0	-	0	0	2	0	0	-	0	0	?	1	0	0	0	1	?	1	0	?	?	0	1	0	2	0	0	0	1	?	0	-	-	-	0	1	0	0	-	0	?	1	0	?	0	0	0	0	?	0	0	0	?	0	1	0	0	0	0	0	?
*Stiltia isabella*	0	0	0	0	0	1	1	1	0	1	1	0	0	1	0	-	0	0	2	0	0	-	0	0	0	1	0	0	0	1	0	1	0	1	0	1	1	0	2	0	0	0	1	1	0	-	-	-	0	1	1	0	-	0	0	1	0	0	0	0	0	0	0	0	0	0	1	0	0	0	0	0	0	0	0
*Rhinoptilus chalcopterus*	0	0	0	0	0	1	1	0	0	?	1	?	1	0	0	-	0	0	2	0	0	-	0	0	?	1	0	0	0	1	?	2	0	?	?	0	1	0	2	0	0	0	0	?	0	-	-	-	1	0	0	0	-	0	?	1	0	?	0	1	1	0	?	0	0	0	?	0	0	0	0	0	0	0	?
*Bartramia longicauda*	0	0	0	1	0	1	1	0	0	1	1	0	0	1	0	-	0	0	2	0	0	-	0	0	0	0	0	0	0	1	0	1	0	1	0	1	1	0	2	0	0	0	0	1	0	-	-	-	0	1	1	1	1	0	0	0	0	0	0	0	1	0	0	0	0	0	1	0	0	0	0	0	0	1	0
*Numenius minutus*	0	0	0	1	0	0	1	1	0	?	1	?	0	1	0	-	0	0	2	0	0	-	0	0	?	0	0	0	0	1	?	1	0	?	?	1	1	0	2	0	0	0	1	?	0	-	-	-	1	0	0	0	-	0	?	1	0	?	0	0	1	0	?	0	0	0	?	0	0	0	0	0	0	0	?
*Tryngites subruficollis*	0	0	1	1	0	0	1	1	0	1	1	1	0	1	0	-	0	0	2	0	0	-	0	0	1	0	0	0	0	1	1	1	0	1	0	1	1	0	2	0	0	0	0	1	0	-	-	-	0	1	0	0	-	-	0	1	0	0	0	0	1	0	0	0	0	0	1	0	0	0	0	0	0	1	0
*Hydrophasianus chirurgus*	0	0	0	0	0	0	0	0	0	0	1	0	0	1	0	-	0	0	2	1	0	-	0	0	1	0	0	0	0	1	0	2	0	0	0	1	1	0	2	0	0	0	0	1	0	-	-	-	0	1	1	0	-	-	1	0	0	1	0	0	0	0	0	0	0	0	0	1	0	0	0	0	0	0	0
*Charadrius vociferus*	0	0	0	1	0	0	0	0	0	1	1	0	0	1	1	1	0	0	2	0	0	-	0	0	1	0	0	0	0	1	1	0	0	1	0	1	1	0	2	0	0	0	0	1	0	-	-	-	0	1	0	0	-	0	0	1	0	0	0	0	1	0	0	0	0	0	1	0	0	0	0	0	0	1	0
*Charadrius wilsonia*	0	0	0	1	0	0	0	0	0	1	1	0	0	1	1	1	0	0	2	0	0	-	0	0	1	0	0	0	0	1	1	0	0	1	0	1	1	0	2	0	0	0	0	1	0	-	-	-	0	1	0	0	-	0	0	1	0	0	0	0	1	0	0	0	0	0	1	0	0	0	0	0	0	1	0
LACM 15410	?	?	0	0	1	?	?	1	0	1	1	0	1	0	?	?	?	0	?	?	1	1	0	?	?	0	?	?	?	?	?	?	?	?	?	?	?	?	?	?	?	?	?	?	?	?	?	?	?	?	?	?	?	?	?	?	?	?	?	?	?	?	?	?	?	2	?	?	?	0	0	0	1	1	1
LACM 128870	?	?	?	?	?	?	?	?	?	?	?	?	?	?	?	?	?	?	?	?	?	?	?	?	?	?	?	?	?	?	?	?	?	?	?	?	1	2	?	?	?	?	?	?	?	?	?	?	?	?	?	0	-	0	?	?	?	?	?	?	2	?	?	?	?	?	0	4	1	0	0	0	1	1	1
LACM 15425	?	?	?	?	?	?	?	?	?	?	?	?	?	?	?	?	?	?	?	?	?	?	?	?	?	?	?	?	?	?	?	?	?	?	?	?	?	?	?	?	?	?	?	?	?	?	?	?	?	?	?	?	?	?	?	?	?	?	?	?	?	?	?	?	?	?	?	?	?	?	?	?	?	?	?
LACM 23739	?	?	?	?	?	?	?	?	?	?	?	?	?	?	?	?	?	?	?	?	?	?	?	?	?	?	?	?	?	?	?	?	?	?	?	?	?	?	?	?	?	?	?	?	?	?	?	?	?	?	?	?	?	?	?	?	?	?	?	?	?	?	?	?	?	?	?	?	?	?	?	?	?	?	?
USNM 243765	?	?	?	?	?	?	?	?	?	?	?	?	?	?	?	?	?	?	?	?	?	?	?	?	?	?	?	?	?	?	?	?	?	?	?	?	?	?	?	?	?	?	?	?	?	?	?	?	?	?	?	?	?	?	?	?	?	?	?	?	?	?	?	?	?	?	?	?	?	?	?	?	?	?	?
LACM 107028	?	?	?	?	?	?	?	?	?	?	?	?	?	?	?	?	?	?	?	?	?	?	?	?	?	?	?	?	?	?	?	?	?	?	?	?	?	?	?	?	?	?	?	?	?	?	?	?	?	?	?	?	?	?	?	?	?	?	?	?	?	?	?	?	?	?	?	?	?	?	?	?	?	?	?
SDSNH 24262	?	?	?	?	?	?	?	?	?	?	?	?	?	?	?	?	?	?	?	?	?	?	?	?	?	?	?	?	?	?	?	?	?	?	?	?	?	?	?	?	?	?	?	?	?	?	?	?	?	?	?	?	?	?	?	?	?	?	?	?	?	?	?	?	?	?	?	?	?	?	?	?	?	?	?
SDSNH 59047	?	?	?	?	?	?	?	?	?	?	?	?	?	?	?	?	?	?	?	?	?	?	?	?	?	?	0	0	2	1	1	2	0	0	0	1	1	2	0	0	0	0	2	1	1	1	0	1	-	-	0	0	-	1	1	1	0	1	0	0	2	1	0	1	1	2	0	4	1	0	0	0	1	1	1
SDSNH 28152	?	?	?	?	?	?	?	?	?	?	?	?	?	?	?	?	?	?	?	?	?	?	?	?	?	?	0	0	2	1	1	2	0	0	0	0	1	2	0	0	0	0	2	0	1	0	0	1	-	-	0	0	-	0	1	1	0	1	0	0	2	1	0	1	1	2	0	4	1	0	0	0	1	1	1
SDSNH 42532	?	?	?	?	?	?	?	?	?	?	?	?	?	?	?	?	?	?	?	?	?	?	?	?	?	?	0	0	2	1	1	2	0	0	0	0	1	2	0	0	0	0	2	0	1	0	0	1	-	-	0	0	-	0	1	1	0	1	0	0	2	1	0	1	1	2	0	4	1	0	0	0	1	1	1
SDSNH 21295	1	1	?	?	?	?	?	0	0	1	1	1	1	1	0	-	1	0	0	0	1	0	0	0	?	0	?	?	?	?	?	?	?	?	?	?	?	?	?	?	?	?	?	?	?	?	?	?	?	?	?	?	?	?	?	?	?	?	?	?	?	?	?	?	?	?	?	?	?	?	?	?	?	?	?
LACM 103940	?	?	?	?	?	?	?	?	?	?	?	?	?	?	?	?	?	?	?	?	?	?	?	?	?	?	?	0	?	?	?	?	?	?	?	?	?	?	?	?	?	?	2	1	1	?	0	?	?	?	?	0	-	?	?	1	0	1	0	?	2	?	0	?	?	?	?	?	?	?	?	?	?	?	?
SDSNH 25236	?	?	?	?	?	?	?	?	?	?	?	?	?	?	?	?	?	?	?	?	?	?	?	?	?	?	?	?	?	?	?	?	?	?	?	?	?	?	?	?	?	?	?	?	?	?	?	?	?	?	?	?	?	?	?	?	?	?	?	?	?	?	?	?	?	?	?	?	?	?	?	?	?	?	?
*Mancalla californiensis*	?	?	?	?	?	?	?	?	?	?	?	?	?	?	?	?	?	?	?	?	?	?	?	?	?	?	0	0	2	1	1	2	0	1	0	1	1	2	1	0	0	0	2	1	1	0	0	0	1	0	0	0	-	0	1	0	0	1	0	0	2	1	1	1	1	2	0	4	?	?	?	?	?	?	?
*Mancalla cedrosensis*	?	?	0	0	1	1	0	0	0	1	1	0	1	1	0	-	1	0	0	0	1	1	0	?	1	0	0	0	2	1	1	2	0	1	0	1	1	2	0	0	0	0	2	1	1	1	0	0	1	0	0	0	-	0	1	1	0	1	0	0	2	1	0	1	1	2	0	4	1	0	0	0	1	1	1
*Mancalla lucasi*	?	?	0	0	1	1	0	?	?	?	?	?	?	?	?	?	?	?	?	?	?	?	?	?	?	?	0	0	2	1	1	2	0	0	0	1	1	2	0	0	0	0	2	1	1	1	0	1	?	-	0	0	-	1	1	1	0	1	0	0	2	1	0	1	1	2	0	4	1	0	0	0	1	1	1
*Mancalla vegrandis*	1	1	0	0	1	1	0	0	0	1	1	0	1	1	0	-	1	0	0	0	1	1	0	0	1	0	0	0	2	1	1	2	0	0	0	0	1	2	0	0	0	0	2	0	1	0	0	1	?	-	0	0	-	0	1	1	0	1	0	0	2	1	0	1	1	2	0	4	1	0	0	0	1	1	1
*Miomancalla howardi*	?	?	?	?	?	?	?	?	?	?	?	?	?	?	?	?	?	?	?	?	?	?	?	?	?	?	0	0	2	0	1	2	0	0	0	1	1	1	1	0	0	0	2	1	1	0	1	1	1	0	2	0	-	0	0	1	0	1	1	?	2	0	0	1	1	2	0	4	1	0	0	0	1	1	1
*Miomancalla wetmorei*	?	?	?	?	?	?	?	?	?	?	?	?	?	?	?	?	?	?	?	?	?	?	?	?	?	?	0	0	2	0	1	2	0	0	0	0	1	1	0	0	0	0	2	0	1	0	1	1	1	0	2	0	-	0	0	1	0	1	1	0	2	0	0	1	1	2	0	4	1	0	?	?	?	?	?
Mancallinae SST	1	1	0	0	1	1	0	A	0	1	1	A	1	1	0	-	1	0	0	0	1	A	0	0	1	0	0	0	2	A	1	2	0	A	0	A	1	C	A	0	0	0	2	A	1	A	A	A	1	0	B	0	-	A	A	A	0	1	A	0	2	A	A	1	1	2	0	4	1	0	0	0	1	1	1
									1	6	0								1	7	0								1	8	0								1	9	0								2	0	0								2	1	0								2	2	0		
*Aethia cristatella*	1	0	1	0	0	0	-	1	0	-	0	-	-	0	0	1	0	1	1	0	1	1	0	1	0	2	0	0	0	0	0	1	1	0	0	0	2	0	0	0	0	0	0	1	1	0	0	0	1	1	1	1	1	1	0	0	0	1	0	1	1	1	1	0	1	0	0	1	0	1	0	1	1	1	0
*Aethia pygmaea*	1	0	1	0	0	1	0	1	1	0	0	-	-	0	1	1	0	1	0	1	0	1	0	1	0	2	0	0	0	0	0	1	1	0	0	0	2	0	2	0	0	0	0	1	1	0	0	0	0	1	1	1	1	1	0	1	0	0	0	0	1	1	1	0	1	0	0	1	1	1	0	0	1	2	0
*Aethia psittacula*	1	0	1	0	0	0	-	1	1	1	0	-	-	0	1	1	0	1	1	0	1	1	0	1	0	2	0	0	0	0	0	1	1	0	0	0	2	0	0	0	0	0	0	1	1	0	0	0	0	1	1	1	1	1	0	0	0	1	0	0	1	1	1	0	1	0	0	1	0	1	0	1	1	1	0
*Aethia pusilla*	1	0	1	0	0	0	-	1	0	-	0	-	-	0	0	1	0	1	1	0	1	1	0	1	0	2	0	0	0	0	0	1	1	0	0	0	2	0	1	0	0	0	0	0	1	0	0	0	0	1	1	1	1	1	0	1	0	0	0	0	1	1	1	0	1	0	0	1	0	1	0	1	1	2	0
*Ptychoramphus aleuticus*	1	0	1	0	0	0	-	1	1	1	0	-	-	0	0	0	0	1	1	0	1	1	0	0	0	2	0	0	0	0	0	1	1	0	0	0	2	0	1	1	0	0	0	0	1	0	1	0	1	1	1	1	1	1	0	0	1	0	0	1	1	1	1	0	1	0	0	0	0	0	0	1	1	0	1
*Cerorhinca monocerata*	1	0	1	0	0	0	-	1	1	1	1	1	2	0	1	1	0	0	1	0	1	1	0	1	0	2	0	0	0	0	0	1	1	0	0	0	1	0	1	1	0	0	0	0	1	0	0	0	1	1	0	0	1	2	0	0	0	1	1	1	1	1	1	0	1	0	0	0	1	1	0	1	1	0	0
*Fratercula arctica*	1	0	1	0	0	0	-	1	1	1	1	1	2	0	1	1	0	0	1	0	1	1	0	1	0	2	0	0	0	0	0	1	1	0	0	0	1	0	1	1	0	0	0	0	1	0	0	0	1	1	0	0	1	2	0	0	0	1	1	0	1	1	1	0	1	0	0	0	1	1	0	1	1	1	0
*Fratercula corniculata*	1	0	1	0	0	0	-	1	1	1	1	1	2	0	1	1	0	0	1	0	1	1	0	1	0	2	0	0	0	0	0	1	1	0	0	0	1	0	1	1	0	0	0	1	1	0	0	0	0	1	0	0	1	2	0	0	0	0	1	0	1	1	1	0	1	0	0	0	1	1	0	1	1	1	0
*Fratercula cirrhata*	1	0	1	0	0	0	-	1	1	1	1	1	0	0	1	1	0	0	1	0	1	1	0	1	0	2	0	0	0	0	0	1	1	0	0	0	1	0	1	1	0	0	0	0	1	0	0	0	1	1	0	0	1	2	0	0	0	1	1	1	1	1	1	0	1	0	0	0	1	1	0	1	1	1	0
*Alca torda*	1	0	1	0	0	1	0	1	1	0	1	1	0	1	0	0	1	0	0	0	0	1	0	1	1	2	1	0	0	0	0	1	1	0	1	1	0	0	0	0	0	1	0	1	0	1	1	1	1	1	0	0	1	1	0	1	0	0	0	0	1	1	0	1	0	0	0	1	1	1	0	1	1	0	1
*Pinguinus impennis*	1	1	1	0	0	1	0	1	0	-	0	-	-	1	0	1	0	0	0	0	0	1	0	1	0	2	0	0	1	0	0	1	1	0	1	2	0	0	0	1	0	1	1	1	0	1	1	1	1	1	0	0	1	1	0	0	1	0	1	1	0	1	0	1	0	0	0	1	1	0	0	1	1	0	1
*Alle alle*	1	0	1	0	0	1	0	0	0	-	1	1	-	0	0	0	0	0	0	1	0	1	0	1	0	2	0	0	0	0	0	1	1	0	1	1	0	0	1	0	0	0	0	0	1	1	0	1	1	1	0	0	1	0	0	1	1	0	0	0	1	1	1	0	1	0	0	1	1	1	0	1	1	0	1
*Uria aalge*	1	0	1	0	0	1	0	1	0	-	0	-	-	1	1	0	0	1	0	1	0	1	0	0	0	2	1	0	0	0	0	1	1	0	1	1	0	0	0	1	0	1	0	1	1	1	1	1	0	1	0	0	1	1	0	1	1	0	0	1	1	1	0	1	0	0	0	1	1	0	0	1	1	2	1
*Uria lomvia*	1	0	1	0	0	1	0	1	0	-	0	-	-	1	1	0	0	1	0	1	0	1	0	1	0	2	1	0	0	0	0	1	1	0	1	1	0	0	0	1	0	1	0	1	1	1	1	1	0	1	0	0	1	1	0	1	1	0	0	1	1	1	0	1	0	0	0	1	1	0	0	1	1	2	1
*Cepphus carbo*	1	0	1	0	0	1	0	0	0	-	1	1	0	1	1	0	0	1	0	1	0	1	0	1	0	1	0	1	1	0	0	1	1	0	0	1	0	0	2	0	0	1	0	1	0	1	1	0	0	0	0	0	1	0	0	0	0	0	1	1	1	1	1	0	1	0	0	1	0	1	0	1	1	0	1
*Cepphus columba*	1	0	1	0	0	0	-	0	1	0	1	1	2	1	0	0	0	1	0	0	0	1	0	1	0	1	0	1	0	0	0	1	1	0	0	1	0	0	1	0	0	1	0	1	1	0	1	0	0	0	0	0	1	0	0	1	0	0	1	0	1	1	1	0	1	0	0	1	0	1	0	1	1	0	1
*Cepphus grylle*	1	0	1	0	0	0	-	0	1	0	1	1	0	1	1	0	0	1	0	0	0	1	0	1	0	1	1	1	0	0	0	1	1	0	0	1	0	0	1	0	0	1	0	1	1	0	0	0	0	0	0	0	1	0	0	1	0	0	1	0	1	1	1	0	1	0	0	1	0	1	0	1	1	0	1
*Brachyramphus brevirostris*	1	0	1	1	0	1	0	0	1	0	0	-	-	1	0	1	1	1	0	1	0	1	0	0	0	2	0	0	0	0	0	1	1	0	0	1	0	0	0	1	0	1	0	1	0	1	0	0	1	1	0	0	1	0	0	1	1	0	0	1	1	1	0	1	0	0	0	1	1	1	0	1	1	0	1
*Brachyramphus marmoratus*	1	0	1	1	0	0	-	0	1	0	0	-	-	1	0	1	0	1	1	0	0	1	0	0	0	2	0	0	0	0	0	1	1	0	0	1	0	0	0	1	0	0	0	1	1	1	0	0	1	1	0	0	1	0	0	1	1	0	0	1	1	1	0	1	0	0	0	0	1	1	0	1	1	0	1
*Brachyramphus perdix*	1	0	1	1	0	1	0	0	1	0	0	-	-	1	1	1	1	1	0	1	0	1	0	0	1	2	0	0	0	0	0	1	1	0	0	1	0	0	0	1	0	1	0	1	0	1	0	0	1	1	0	0	1	0	0	1	1	0	1	1	1	1	0	1	0	0	0	1	1	1	0	1	1	0	1
*Synthliboramphus antiquus*	1	0	1	0	0	1	0	0	0	-	0	-	-	1	1	0	1	1	1	0	1	1	0	0	0	2	0	0	0	0	0	1	1	0	0	2	0	0	0	0	0	1	0	0	0	1	1	1	1	1	0	0	1	0	1	1	1	0	0	0	1	0	1	0	0	1	0	1	0	0	1	0	0	0	2
*Synthliboramphus wumizusume*	1	0	1	0	0	1	0	0	1	0	0	-	-	1	1	0	1	1	0	1	0	1	0	1	0	2	0	0	1	0	0	1	1	0	0	1	0	0	2	0	0	1	0	0	0	1	0	1	1	1	0	0	1	0	0	1	1	0	0	0	1	0	1	0	0	1	0	1	0	1	1	0	0	0	2
*Synthliboramphus craveri*	1	0	1	0	0	1	0	0	1	0	0	-	-	1	1	0	1	1	0	1	0	1	0	0	1	2	0	0	1	0	0	1	1	0	0	2	0	0	2	0	0	1	0	1	0	1	0	1	1	1	0	0	1	0	0	1	1	0	0	0	1	0	1	0	0	1	0	1	0	1	1	0	0	0	1
*Synthliboramphus hypoleucus*	1	0	1	0	0	1	0	0	0	-	0	-	-	1	1	0	1	1	1	0	0	1	0	0	0	2	1	0	0	0	0	1	1	0	0	2	0	0	0	0	0	0	0	0	0	0	1	1	1	1	0	0	1	0	1	1	1	0	0	0	1	0	1	0	0	0	0	1	0	0	1	0	0	0	1
*Stercorarius longicaudus*	0	0	0	0	0	1	0	1	1	0	1	0	-	0	1	0	1	0	0	0	0	0	1	0	1	0	1	0	0	0	0	1	1	0	0	0	0	0	0	1	1	1	0	1	1	0	1	0	1	0	0	0	0	1	0	0	0	1	1	0	1	0	0	0	0	0	1	0	0	0	0	1	0	0	1
*Stercorarius skua*	0	0	0	0	0	1	0	1	1	0	1	0	-	0	1	0	0	1	1	0	0	0	1	1	1	0	0	0	0	0	0	1	1	0	0	0	0	0	0	1	1	0	1	0	1	0	0	0	1	0	0	1	0	0	1	0	0	1	1	0	1	0	0	0	0	1	1	1	0	0	0	1	0	0	1
*Anous tenuirostris*	0	0	0	0	0	1	0	1	1	0	1	0	-	0	1	1	1	1	0	0	0	0	2	1	1	0	0	0	1	0	0	1	1	0	0	0	1	0	0	0	1	0	0	0	1	0	0	0	1	0	0	1	0	0	0	1	1	1	0	1	1	0	1	2	0	0	1	1	0	0	0	1	1	0	1
*Chlidonias leucoptera*	0	0	0	0	0	1	0	1	1	0	1	0	-	0	1	1	0	0	0	0	0	0	1	1	1	0	0	0	0	0	0	1	1	0	0	0	1	0	1	0	1	0	1	1	1	0	0	0	0	0	0	0	0	2	0	1	0	1	1	1	1	0	1	2	0	0	0	0	0	0	0	0	1	0	1
*Sterna niloteca*	0	0	0	0	0	1	0	0	1	0	1	0	-	0	1	0	1	1	0	0	0	0	1	1	1	0	0	0	0	0	0	1	1	0	0	0	1	0	0	0	1	0	0	0	1	0	1	0	0	0	0	1	1	0	1	1	0	1	1	1	1	0	1	0	0	1	1	1	0	1	0	1	0	0	1
*Larosterna inca*	0	0	0	0	0	1	0	1	1	0	1	0	-	0	1	0	0	1	0	0	0	0	1	1	0	0	0	0	0	0	0	1	1	0	0	0	1	0	1	0	1	0	1	1	1	0	1	0	0	0	0	0	0	0	0	0	0	1	1	0	1	1	1	0	0	0	1	0	1	1	0	1	1	1	0
*Sterna maxima*	0	0	0	0	0	1	0	1	1	2	1	0	-	0	1	0	1	0	0	0	0	0	1	1	1	0	0	0	0	0	0	1	1	0	0	0	1	0	0	0	1	1	1	0	1	0	1	0	0	0	0	1	0	0	0	0	0	1	1	0	1	1	1	0	0	0	1	0	0	1	0	1	1	0	0
*Phaetusa simplex*	0	0	0	0	0	1	0	1	1	2	1	0	-	0	1	0	1	1	0	0	0	0	1	1	1	0	0	0	0	0	0	1	1	0	0	0	1	0	1	0	1	0	1	0	1	0	0	0	0	0	0	0	0	0	0	0	1	1	1	1	1	0	1	2	0	1	0	0	0	1	0	1	1	1	0
*Sternula superciliaris*	0	0	0	?	0	?	?	1	0	-	1	0	-	0	1	0	1	1	0	0	0	0	1	1	1	0	1	0	1	0	0	1	1	0	0	0	1	0	1	1	1	0	1	1	1	0	0	1	0	0	0	1	0	0	1	1	0	1	1	1	1	0	1	0	0	0	1	0	0	?	0	1	1	1	0
*Sterna anaethetus*	0	0	0	0	0	1	0	0	0	-	1	0	-	0	1	0	1	1	0	0	0	0	1	1	1	0	0	0	0	0	0	1	1	0	0	0	0	0	0	0	1	0	1	0	1	0	1	1	0	0	0	1	1	0	1	0	0	1	1	1	1	1	1	0	0	0	0	1	1	1	0	1	1	0	1
*Gygis alba*	0	0	0	0	0	1	0	0	0	-	1	0	-	0	1	0	1	0	0	0	0	0	1	1	1	0	0	0	0	0	0	1	1	0	0	0	1	0	0	0	1	0	1	0	1	0	1	1	0	0	0	1	1	1	0	1	0	1	0	1	1	1	1	0	0	0	0	1	1	1	0	0	1	0	1
*Rynchops niger*	1	0	0	0	0	1	0	1	1	0	1	0	-	0	1	0	1	0	0	0	0	0	1	0	1	0	0	0	0	0	0	1	1	0	0	0	0	0	0	1	1	0	1	1	1	0	1	0	0	0	1	1	0	0	0	1	0	1	1	1	1	0	0	0	0	0	1	0	0	1	0	1	1	0	0
*Creagrus furcatus*	0	0	0	0	0	0	-	1	1	0	1	0	-	0	0	1	1	1	0	0	0	0	1	1	1	0	0	0	1	0	0	1	1	0	0	0	0	0	0	1	1	0	0	1	1	0	1	0	0	0	0	0	0	0	0	0	1	1	1	0	1	0	0	0	0	1	1	1	0	1	0	1	0	1	1
*Rhodostethia rosea*	0	0	0	0	0	1	0	1	1	0	1	0	-	0	0	0	0	1	0	0	0	0	1	1	1	0	1	0	0	0	0	1	1	0	0	0	1	0	1	1	1	0	1	1	1	0	0	0	0	0	0	0	0	0	0	1	1	1	1	1	1	0	0	0	0	0	1	1	0	0	0	1	0	0	1
*Larus argentatus*	0	0	0	0	0	1	0	1	1	0	1	0	-	0	0	0	0	1	0	0	0	0	1	1	1	0	1	0	0	0	0	1	1	0	0	0	1	0	0	0	1	1	0	1	1	0	1	0	0	0	0	1	0	0	0	0	0	1	1	1	1	0	0	2	0	1	1	1	0	1	0	1	0	1	0
*Larus marinus*	0	0	0	0	0	1	0	1	1	0	1	0	-	0	0	0	0	1	0	0	0	0	1	1	1	0	0	0	0	0	0	1	1	0	0	0	1	0	0	0	1	1	0	1	1	0	1	0	0	0	0	1	0	0	0	0	0	0	1	0	1	0	0	2	0	1	1	0	0	1	0	1	0	0	0
*Pagophila eburnea*	0	0	0	0	0	1	0	1	1	0	1	0	-	0	0	0	1	0	0	0	0	0	1	1	1	0	0	0	0	0	0	1	1	0	0	0	1	0	0	0	1	1	1	1	1	0	1	1	0	0	0	1	1	1	1	0	0	1	1	1	1	1	0	0	0	0	1	1	0	1	0	1	1	1	1
*Xema sabini*	0	0	0	0	0	1	0	1	1	0	1	0	-	0	1	0	0	0	0	0	0	0	1	1	1	0	0	0	0	0	0	1	1	0	0	0	1	0	0	0	1	0	0	1	1	0	1	0	0	0	0	1	1	1	1	1	0	1	1	1	1	0	0	2	0	1	1	1	0	1	0	1	0	1	1
*Rissa tridactyla*	0	0	0	0	0	0	-	0	1	0	1	0	-	0	1	0	0	1	0	0	0	0	1	1	1	0	0	0	0	0	0	1	1	0	0	0	0	0	0	0	1	0	0	1	1	0	1	1	0	0	0	1	1	0	1	0	0	1	1	1	1	1	0	0	0	0	1	1	0	0	0	1	1	0	1
*Cusorius temmneckii*	0	0	0	?	0	?	?	1	0	-	1	0	-	0	0	1	1	1	0	0	0	0	1	1	1	0	1	0	1	0	0	1	1	0	0	0	1	0	0	0	1	0	0	1	1	0	1	1	0	0	0	1	0	0	1	0	0	1	1	1	1	0	1	0	1	0	1	1	0	?	1	0	0	0	1
*Glareola maldivarum*	0	0	0	?	0	?	?	1	1	2	?	?	?	0	0	1	1	1	0	0	0	0	1	1	1	0	0	0	1	0	0	1	1	0	0	0	1	0	1	0	1	0	1	1	1	0	1	1	0	0	0	0	0	0	0	0	1	1	1	1	1	0	0	1	0	1	0	1	0	?	1	0	0	0	1
*Stiltia isabella*	0	0	0	0	0	0	-	1	1	0	1	0	-	0	0	0	1	1	0	0	0	0	1	1	0	0	0	0	0	0	0	1	1	0	0	0	0	0	1	1	1	0	0	0	1	0	1	0	0	0	0	0	0	0	1	0	0	1	0	1	1	0	0	2	0	1	1	1	0	0	1	0	0	0	0
*Rhinoptilus chalcopterus*	0	0	0	?	0	?	?	1	1	0	1	0	-	0	0	0	1	1	0	0	0	0	1	1	0	0	1	0	1	0	0	1	1	0	0	0	2	0	0	0	0	0	0	0	1	0	1	1	1	0	0	1	0	0	1	0	0	1	0	1	1	0	1	0	0	1	1	0	0	?	1	0	0	0	1
*Bartramia longicauda*	1	0	0	0	0	1	0	1	1	0	1	0	-	0	0	1	0	0	0	0	0	0	2	1	1	0	0	0	0	0	0	1	1	0	0	0	0	0	0	1	0	0	0	0	1	0	1	0	0	0	0	0	0	0	0	1	0	1	1	1	1	0	1	2	0	1	1	1	0	0	0	0	0	0	1
*Numenius minutus*	1	0	0	?	0	?	?	1	1	0	1	0	-	0	0	0	1	0	0	0	0	0	1	1	0	0	1	0	0	0	0	1	1	0	0	0	1	0	1	1	0	0	0	1	1	0	1	0	0	0	0	0	0	0	1	0	0	0	1	0	1	0	1	2	0	0	1	1	0	?	1	0	0	0	1
*Tryngites subruficollis*	0	0	0	0	0	1	0	1	1	0	1	0	-	0	0	0	1	1	0	0	0	0	1	1	0	0	0	0	0	0	0	1	1	0	0	0	0	0	1	1	0	0	0	0	1	0	0	0	0	0	0	0	0	1	0	1	0	1	0	1	1	0	1	2	0	0	1	1	0	1	0	1	0	0	1
*Hydrophasianus chirurgus*	0	0	0	0	0	0	-	1	1	0	1	0	-	0	1	0	1	1	0	0	0	0	1	1	0	0	0	0	0	0	0	1	1	0	0	0	0	0	0	0	0	0	0	0	1	0	0	0	0	0	0	0	0	0	1	0	0	1	0	1	1	0	1	2	0	1	1	0	0	0	0	1	0	0	1
*Charadrius vociferus*	1	0	0	0	0	0	-	1	1	0	1	0	-	0	0	1	1	1	0	0	0	0	1	0	0	0	0	0	0	0	0	1	1	0	0	0	0	0	0	0	0	0	0	0	1	0	1	1	0	0	0	0	0	0	1	0	0	1	1	0	1	0	1	2	0	1	1	1	0	1	0	0	0	0	1
*Charadrius wilsonia*	1	0	0	0	0	0	-	1	0	-	1	0	-	0	0	1	1	1	0	0	0	0	1	1	0	0	0	0	0	0	0	1	1	0	0	0	0	0	0	1	0	0	0	0	1	0	1	1	0	0	0	1	0	0	1	0	0	1	1	0	1	0	1	2	0	1	1	1	0	1	0	0	0	0	1
LACM 15410	1	0	0	0	1	1	0	1	0	-	?	?	?	?	?	?	?	?	?	?	?	?	?	?	?	?	?	?	?	?	?	?	?	?	?	?	?	?	?	?	?	?	?	?	?	?	?	?	?	?	?	?	?	?	?	?	?	?	?	?	?	?	?	?	?	?	?	?	?	?	?	?	?	?	?
LACM 128870	1	0	0	0	1	1	0	1	0	-	?	?	?	?	?	2	0	1	0	0	0	0	0	0	1	2	0	?	?	?	1	0	?	?	?	?	?	?	?	?	?	?	?	?	?	?	?	?	?	?	?	?	?	?	?	?	?	?	?	?	?	?	?	?	?	?	?	?	?	?	?	?	?	?	?
LACM 15425	?	?	?	?	?	?	?	?	?	?	?	?	?	?	?	?	?	?	?	?	?	?	?	?	?	?	?	?	?	?	?	?	?	?	?	?	?	?	?	?	?	?	?	?	?	?	?	?	?	?	?	?	?	?	?	1	?	0	0	?	1	?	?	?	?	?	?	?	?	?	?	?	?	?	?
LACM 23739	?	?	?	?	?	?	?	?	?	?	?	?	?	?	?	?	?	?	?	?	?	?	?	?	?	?	?	?	?	?	?	?	?	?	?	?	?	?	?	?	?	?	?	?	?	?	?	?	?	?	?	?	?	0	0	?	?	0	0	1	?	1	?	?	?	1	0	0	1	1	0	0	1	?	?
USNM 243765	?	?	?	?	?	?	?	?	?	?	?	?	?	?	?	2	0	1	0	1	1	0	0	0	1	2	0	0	0	?	1	0	?	?	?	?	?	?	?	?	?	?	?	?	?	?	?	?	?	?	?	?	?	?	?	?	?	?	?	?	?	?	?	?	?	?	?	?	?	?	?	?	?	?	?
LACM 107028	?	?	?	?	?	?	?	?	?	?	?	?	?	?	?	2	0	1	0	1	1	0	0	0	1	2	0	0	0	1	1	0	0	1	-	2	?	1	0	?	?	?	?	?	?	?	?	?	?	?	?	?	?	?	?	?	0	?	1	1	0	1	1	0	1	1	0	0	1	1	0	0	?	?	?
SDSNH 24262	?	?	?	?	?	?	?	?	?	?	?	?	?	?	?	?	?	?	?	?	?	?	?	?	?	?	?	?	?	?	?	?	?	?	?	?	?	?	?	?	?	?	?	?	?	?	?	?	?	?	?	?	?	?	?	?	?	?	?	?	?	?	?	?	?	?	?	?	?	?	?	?	?	?	?
SDSNH 59047	1	0	0	0	1	1	1	1	1	-	?	?	?	?	?	?	?	?	?	?	?	?	?	?	?	?	?	?	?	?	?	?	?	?	?	?	?	?	?	?	?	?	?	?	?	?	?	?	?	?	?	?	?	?	?	?	?	?	?	?	?	?	?	?	?	?	?	?	?	?	?	?	?	?	?
SDSNH 28152	1	0	0	0	1	1	0	1	0	-	?	?	?	?	?	?	?	?	?	?	?	?	?	?	?	?	?	?	?	?	?	?	?	?	?	?	?	?	?	?	?	?	?	?	?	?	?	?	?	?	?	?	?	?	?	?	?	?	?	?	?	?	?	?	?	?	?	?	?	?	?	?	?	?	?
SDSNH 42532	1	0	0	0	1	1	0	1	0	-	?	?	?	?	?	?	?	?	?	?	?	?	?	?	?	?	?	?	?	?	?	?	?	?	?	?	?	?	?	?	?	?	?	?	?	?	?	?	?	?	?	?	?	?	?	?	?	?	?	?	?	?	?	?	?	?	?	?	?	?	?	?	?	?	?
SDSNH 21295	?	?	?	?	?	?	?	?	?	?	?	?	?	?	?	?	?	?	?	?	?	?	?	?	?	?	?	?	?	?	?	?	?	?	?	?	?	?	?	?	?	?	?	?	?	?	?	?	?	?	?	?	?	?	?	?	?	?	?	?	?	?	?	?	?	?	?	?	?	?	?	?	?	?	?
LACM 103940	?	?	?	?	?	?	?	?	?	?	?	?	?	?	?	?	?	?	?	?	?	?	?	?	?	?	?	?	?	?	?	?	?	?	?	?	?	?	?	?	?	?	?	?	?	?	?	?	?	?	?	?	?	?	?	?	?	?	?	?	?	?	?	?	?	?	?	?	?	?	?	?	?	?	?
SDSNH 25236	?	?	?	?	?	?	?	?	?	?	?	?	?	?	?	?	?	?	?	?	?	?	?	?	?	?	?	?	?	?	?	?	?	?	?	?	?	?	?	?	?	?	?	?	?	?	?	?	?	?	?	?	?	?	?	?	?	?	?	?	?	?	?	?	?	?	?	?	?	?	?	?	?	?	?
*Mancalla californiensis*	?	?	?	?	?	?	?	?	?	?	?	?	?	?	?	?	?	?	?	?	?	?	?	?	?	?	?	?	?	?	?	?	?	?	?	?	?	?	?	?	?	?	?	?	?	?	?	?	?	?	?	?	?	?	?	?	?	?	?	?	?	?	?	?	?	?	?	?	?	?	?	?	?	?	?
*Mancalla cedrosensis*	1	0	0	0	1	1	0	0	0	-	1	1	2	1	0	2	0	1	0	0	0	0	0	1	1	2	1	0	0	1	1	0	0	1	-	2	2	1	0	1	?	?	?	?	?	?	?	?	?	?	?	?	?	0	0	1	0	0	0	1	1	1	?	?	?	1	0	0	1	1	0	0	1	?	?
*Mancalla lucasi*	1	0	0	0	1	1	1	1	1	-	?	?	?	?	?	?	?	?	?	?	?	?	?	?	?	?	?	?	?	?	?	?	?	?	?	?	?	?	?	?	?	?	?	?	?	?	?	?	?	?	?	?	?	?	?	?	?	?	?	?	?	?	?	?	?	?	?	?	?	?	?	?	?	?	?
*Mancalla vegrandis*	1	0	0	0	1	1	0	1	0	-	?	?	?	?	?	?	?	?	?	?	?	?	?	?	?	?	?	?	?	?	?	?	?	?	?	?	?	?	?	?	?	?	?	1	?	?	1	1	1	1	0	?	1	?	?	?	?	?	?	?	?	?	?	?	?	?	?	?	?	?	?	?	?	?	?
*Miomancalla howardi*	1	0	0	0	1	1	0	1	1	0	?	?	?	?	?	?	?	?	?	?	?	?	?	?	?	?	?	?	?	?	?	?	0	1	-	2	2	?	?	1	?	?	?	1	?	?	1	?	1	?	0	?	1	0	0	?	?	?	?	?	?	?	?	?	?	?	?	0	?	?	?	?	?	?	?
*Miomancalla wetmorei*	1	0	0	0	0	?	?	1	0	-	?	?	?	?	?	?	?	?	?	?	?	?	?	?	?	?	?	?	?	?	?	?	?	?	?	?	?	?	?	?	?	?	?	?	?	?	?	?	?	?	?	?	?	?	?	?	?	?	?	?	?	?	?	?	?	?	?	?	?	?	?	?	?	?	?
Mancallinae SST	1	0	0	0	A	1	A	A	A	0	1	1	2	1	0	2	0	1	0	A	A	0	0	A	1	2	A	0	0	1	1	0	0	1	-	2	2	1	0	1	?	?	?	1	?	?	1	1	1	1	0	?	1	0	0	1	0	0	A	1	A	1	1	0	1	1	0	0	1	1	0	0	1	?	?
		2	3	0								2	4	0								2	5	0								2	6	0								2	7	0								2	8	0								2	9	0								3	0	0
*Aethia cristatella*	0	0	0	2	1	1	1	0	0	1	1	0	0	0	0	0	2	0	1	0	1	0	1	1	0	1	0	0	3	0	0	1	0	0	0	1	0	0	0	1	2	0	0	?	?	?	?	?	?	?	?	?	?	?	?	?	?	?	?	?	?	?	?	?	?	?	?	?	?	?	?	?	?	?	?
*Aethia pygmaea*	0	0	0	2	1	1	1	0	1	1	1	0	0	0	0	0	2	0	1	0	1	0	1	1	0	1	0	0	3	0	0	1	0	0	0	0	0	0	0	1	2	0	0	?	?	?	?	?	?	?	?	?	?	?	?	?	?	?	?	?	?	?	?	?	?	?	?	?	?	?	?	?	?	?	?
*Aethia psittacula*	0	0	0	2	1	1	1	0	0	1	0	0	0	0	0	0	2	0	0	0	1	0	1	1	0	1	0	0	0	0	0	1	0	0	0	3	0	0	0	1	2	0	0	?	?	?	?	?	?	?	?	?	?	?	?	?	?	?	?	?	?	?	?	?	?	?	?	1	1	1	2	1	2	0	-
*Aethia pusilla*	0	1	1	2	1	1	1	0	0	1	0	0	0	0	0	0	2	0	0	0	1	0	1	1	0	1	0	0	0	0	0	1	0	0	0	0	0	0	0	1	2	0	0	?	?	?	?	?	?	?	?	?	?	?	?	?	?	?	?	?	?	?	?	?	?	?	?	1	1	1	1	1	2	0	-
*Ptychoramphus aleuticus*	0	0	0	2	0	1	1	0	0	0	0	0	0	0	0	0	2	0	1	0	1	0	1	1	0	1	0	0	0	0	0	0	0	0	0	0	0	0	0	2	2	0	0	1	0	1	1	1	0	0	0	0	1	1	1	0	1	0	0	0	0	0	1	1	0	1	1	1	1	1	0	1	2	0	-
*Cerorhinca monocerata*	0	1	1	?	1	0	0	0	0	1	0	0	0	0	0	0	3	0	1	0	0	0	0	1	0	1	0	0	0	0	0	2	0	0	0	3	0	0	0	1	2	1	1	0	0	0	0	1	0	1	0	1	0	0	0	0	0	0	0	0	1	1	1	0	0	0	1	1	1	1	0	1	2	0	-
*Fratercula arctica*	1	0	1	0	1	0	A	1	0	0	0	0	0	0	0	0	3	0	0	0	0	1	0	1	1	1	0	1	0	0	0	2	0	0	0	3	0	0	0	1	2	1	1	0	0	0	0	1	0	1	0	1	0	0	0	0	0	0	0	0	1	1	1	0	0	1	1	1	1	1	1	1	2	0	-
*Fratercula corniculata*	1	0	1	1	1	0	0	1	0	0	0	0	0	0	0	0	3	0	0	0	0	1	0	1	1	1	0	1	0	0	0	2	0	0	0	3	0	0	0	1	2	1	1	0	0	0	0	1	0	1	0	1	0	0	0	0	0	0	0	0	1	1	1	0	0	1	1	?	?	?	?	?	?	?	?
*Fratercula cirrhata*	1	0	1	?	1	0	1	0	0	1	0	0	0	0	0	0	3	0	1	0	0	1	0	1	1	1	0	1	1	0	0	2	0	0	0	3	0	0	0	2	2	2	1	0	0	0	0	1	0	1	0	1	0	0	0	0	0	0	0	0	1	1	1	0	0	0	1	1	1	1	1	1	2	0	-
*Alca torda*	1	0	1	0	0	2	0	0	0	0	0	1	1	1	0	0	1	1	0	1	0	0	2	0	0	1	0	0	0	0	1	0	0	0	0	1	1	1	0	2	0	1	1	0	1	1	0	1	1	0	0	2	0	0	1	1	1	1	0	0	0	0	1	1	0	0	1	1	1	1	2	1	2	0	-
*Pinguinus impennis*	1	0	1	1	0	2	0	0	0	0	0	1	1	1	0	0	1	1	0	?	?	0	2	0	0	1	0	0	0	0	1	0	0	0	0	1	1	1	0	?	0	1	?	?	?	?	?	?	?	?	?	?	?	?	?	?	?	?	?	?	?	?	?	?	?	?	?	?	?	?	?	?	?	?	?
*Alle alle*	0	0	0	0	0	1	0	0	0	0	0	1	0	1	0	0	1	0	0	0	0	0	2	0	0	1	0	0	0	0	0	1	0	0	0	0	0	0	0	2	2	0	0	?	?	?	?	?	?	?	?	?	?	?	?	?	?	?	?	?	?	?	?	?	?	?	?	1	1	1	2	1	2	0	-
*Uria aalge*	0	0	1	0	0	2	0	0	0	0	0	1	0	1	0	0	1	0	0	0	0	0	2	0	0	1	0	0	0	0	1	0	0	0	0	1	1	1	0	2	0	1	1	0	1	0	0	1	1	0	0	2	1	0	1	1	1	1	0	0	0	0	1	0	0	1	1	1	1	1	0	1	2	0	-
*Uria lomvia*	0	0	1	0	0	2	0	0	0	0	0	1	1	1	0	0	1	0	0	0	0	0	2	0	0	1	0	0	0	0	1	0	0	0	0	1	1	1	0	2	0	2	1	0	1	0	0	1	1	0	0	2	1	0	1	1	1	1	0	0	0	0	1	0	0	1	1	?	?	?	?	?	?	?	?
*Cepphus carbo*	0	0	0	1	0	1	0	0	0	0	0	0	0	0	0	0	2	0	0	0	0	0	0	0	0	1	0	0	1	0	0	1	0	0	1	?	?	?	?	?	2	?	1	?	?	?	?	?	?	?	?	?	?	?	?	?	?	?	?	?	?	?	?	?	?	?	?	?	?	?	?	?	?	?	?
*Cepphus columba*	0	0	0	1	0	1	0	0	0	0	0	0	0	0	1	0	2	0	0	0	0	0	0	0	0	1	0	0	1	0	0	1	0	0	1	3	0	0	0	2	2	2	1	0	1	0	0	1	0	0	1	2	1	0	1	1	1	1	0	0	0	0	0	0	0	1	1	1	1	1	1	1	2	0	-
*Cepphus grylle*	0	0	0	1	0	1	0	0	0	0	0	0	0	0	1	0	1	0	0	0	0	0	0	0	0	1	0	0	1	0	0	1	0	0	1	3	0	0	0	2	2	2	1	0	1	0	0	1	0	0	1	2	1	0	1	1	1	1	0	0	0	0	0	0	0	1	1	?	?	?	?	?	?	?	?
*Brachyramphus brevirostris*	0	0	0	2	0	1	0	0	0	0	0	0	0	0	0	0	2	0	0	0	0	0	1	1	0	1	0	0	0	1	1	0	1	1	0	2	0	1	0	3	2	1	?	?	?	?	?	?	?	?	?	?	?	?	?	?	?	?	?	?	?	?	?	?	?	?	?	?	?	?	?	?	?	?	?
*Brachyramphus marmoratus*	0	0	0	2	0	1	0	0	0	0	0	0	0	0	0	0	2	0	0	0	0	0	1	1	0	1	0	0	2	1	1	4	1	1	0	2	0	1	0	4	2	0	1	0	1	1	0	1	0	0	0	2	1	0	1	1	1	0	1	1	0	0	1	1	1	1	1	1	1	1	1	1	2	0	-
*Brachyramphus perdix*	0	0	0	2	0	1	0	0	0	0	0	0	0	0	0	0	2	0	0	0	0	0	1	1	0	1	0	0	2	1	1	0	1	1	0	?	?	?	?	?	2	0	1	?	?	?	?	?	?	?	?	?	?	?	?	?	?	?	?	?	?	?	?	?	?	?	?	?	?	?	?	?	?	?	?
*Synthliboramphus antiquus*	0	0	0	2	0	1	0	0	0	0	0	0	0	0	0	0	1	0	0	0	0	0	1	1	0	1	0	0	0	0	0	1	0	0	1	3	0	0	1	2	1	2	2	0	1	1	1	0	0	0	0	2	1	1	1	1	1	1	0	0	0	0	1	1	1	1	1	1	1	1	0	1	2	0	-
*Synthliboramphus wumizusume*	0	0	0	2	0	1	0	0	0	0	0	0	0	0	0	0	1	0	0	0	0	0	1	1	0	1	0	0	0	0	0	1	0	0	1	?	?	?	?	?	1	0	0	?	?	?	?	?	?	?	?	?	?	?	?	?	?	?	?	?	?	?	?	?	?	?	?	?	?	?	?	?	?	?	?
*Synthliboramphus craveri*	0	0	0	2	0	1	0	0	0	0	0	0	0	0	0	0	1	0	0	0	0	0	1	1	0	1	0	0	0	0	0	1	0	0	1	3	0	0	1	?	1	1	1	?	?	?	?	?	?	?	?	?	?	?	?	?	?	?	?	?	?	?	?	?	?	?	?	?	?	?	?	?	?	?	?
*Synthliboramphus hypoleucus*	0	0	0	2	0	1	0	0	0	0	0	0	0	0	0	0	1	0	0	0	0	0	1	1	0	1	0	0	0	0	0	1	0	0	1	2	1	0	1	2	1	1	?	?	?	?	?	?	?	?	?	?	?	?	?	?	?	?	?	?	?	?	?	?	?	?	?	?	?	?	?	?	?	?	?
*Stercorarius longicaudus*	0	0	0	?	0	0	0	0	0	0	0	0	0	0	0	1	1	0	0	?	1	2	1	1	0	1	1	0	0	1	0	0	0	1	0	3	1	1	1	3	2	2	2	?	?	?	?	?	?	?	?	?	?	?	?	?	?	?	?	?	?	?	?	?	?	?	?	1	0	-	1	1	1	1	0
*Stercorarius skua*	0	0	0	?	0	0	0	0	0	0	0	0	0	0	1	1	1	0	1	?	1	2	0	0	0	1	1	0	2	1	0	0	0	1	1	3	1	0	1	?	2	1	1	0	0	?	0	1	0	?	?	2	?	0	0	0	0	0	0	?	0	0	0	1	1	0	1	1	0	-	0	0	1	1	0
*Anous tenuirostris*	0	0	0	?	0	0	0	0	0	0	0	0	0	0	0	1	0	1	1	-	?	0	2	0	0	1	1	0	1	0	?	4	0	0	0	?	?	?	?	?	?	2	2	?	?	?	?	?	?	?	?	?	?	?	?	?	?	?	?	?	?	?	?	?	?	?	?	?	?	?	?	?	?	?	?
*Chlidonias leucoptera*	0	0	0	?	0	0	0	0	0	0	0	0	0	0	0	1	0	0	-	0	?	2	0	0	0	0	1	0	1	0	?	3	0	1	1	?	?	?	?	4	?	0	0	?	?	?	?	?	?	?	?	?	?	?	?	?	?	?	?	?	?	?	?	?	?	?	?	?	?	?	?	?	?	?	?
*Sterna niloteca*	0	0	0	?	0	0	0	0	0	0	0	0	0	1	0	1	1	0	0	-	1	2	1	0	0	1	1	0	0	0	?	0	0	1	1	3	0	0	0	0	?	0	0	0	0	?	0	0	0	1	1	?	?	0	0	1	0	1	?	0	0	0	0	1	0	1	0	1	0	-	1	0	1	1	1
*Larosterna inca*	0	0	0	?	0	0	0	0	0	1	0	0	0	1	1	1	1	0	-	-	1	1	0	1	0	1	1	0	3	0	?	1	1	0	?	?	?	?	?	3	?	1	?	?	?	?	?	?	?	?	?	?	?	?	?	?	?	?	?	?	?	?	?	?	?	?	?	1	0	-	1	1	1	1	0
*Sterna maxima*	3	0	0	?	0	0	0	0	0	1	0	0	0	0	0	0	1	1	0	-	1	2	2	1	0	1	1	1	0	0	0	0	0	0	0	1	1	1	0	0	1	1	1	0	0	?	0	0	0	1	1	?	?	0	0	1	0	1	?	0	0	0	0	1	0	1	0	?	?	?	?	?	?	?	?
*Phaetusa simplex*	0	0	0	?	0	0	0	0	0	0	0	0	0	1	0	1	0	0	0	-	1	1	3	0	0	1	1	0	0	0	?	0	A	1	1	?	?	?	?	?	?	1	1	?	?	?	?	?	?	?	?	?	?	?	?	?	?	?	?	?	?	?	?	?	?	?	?	1	0	-	1	0	1	1	2
*Sternula superciliaris*	0	0	0	?	0	0	0	0	0	0	0	0	0	0	0	1	1	0	0	?	1	3	2	0	0	1	1	0	0	0	?	0	0	1	1	?	?	?	?	?	?	0	?	?	?	?	?	?	?	?	?	?	?	?	?	?	?	?	?	?	?	?	?	?	?	?	?	?	?	?	?	?	?	?	?
*Sterna anaethetus*	0	0	0	?	0	0	0	0	0	0	0	0	0	0	0	1	1	0	0	-	1	2	2	1	0	1	1	0	0	0	?	0	0	0	0	?	?	?	?	3	?	1	1	?	?	?	?	?	?	?	?	?	?	?	?	?	?	?	?	?	?	?	?	?	?	?	?	?	?	?	?	?	?	?	?
*Gygis alba*	0	0	0	?	0	0	0	0	0	0	0	0	0	1	0	0	1	0	-	-	1	1	1	0	0	1	1	1	0	0	?	4	1	0	0	?	?	?	?	4	?	1	1	?	?	?	?	?	?	?	?	?	?	?	?	?	?	?	?	?	?	?	?	?	?	?	?	1	0	-	1	0	1	1	2
*Rynchops niger*	1	0	0	?	0	0	0	0	0	0	0	0	0	1	0	0	1	1	0	?	1	2	0	0	0	1	1	1	0	0	0	0	0	0	0	3	0	0	0	1	2	1	1	0	0	?	0	0	0	?	0	1	?	0	0	0	0	0	0	?	0	0	1	1	0	0	0	1	0	-	0	0	1	1	0
*Creagrus furcatus*	0	0	0	?	0	0	0	0	0	0	0	0	0	1	0	0	1	0	0	?	1	1	0	0	0	1	1	0	0	0	?	0	0	0	0	?	?	?	?	?	0	1	1	?	?	?	?	?	?	?	?	?	?	?	?	?	?	?	?	?	?	?	?	?	?	?	?	1	0	-	0	0	1	1	2
*Rhodostethia rosea*	0	0	0	?	0	0	0	0	0	0	0	0	0	1	0	1	3	0	1	-	1	3	0	0	0	1	1	1	-	0	?	0	0	0	1	?	?	?	?	4	?	0	0	?	?	?	?	?	?	?	?	?	?	?	?	?	?	?	?	?	?	?	?	?	?	?	?	1	0	-	1	0	1	1	0
*Larus argentatus*	0	0	0	?	0	0	1	0	0	0	0	0	0	0	0	0	1	0	0	0	1	0	0	1	0	1	1	1	0	0	0	3	0	0	0	1	0	1	0	4	0	2	2	0	0	?	0	0	0	?	1	2	?	0	0	0	0	0	0	?	0	0	0	1	0	1	1	1	0	-	0	0	1	1	2
*Larus marinus*	0	0	0	?	0	0	1	0	0	0	0	0	0	0	0	0	1	0	0	0	1	0	0	1	0	1	1	1	0	0	0	3	0	0	0	0	0	1	0	3	0	2	2	?	?	?	?	?	?	?	?	?	?	?	?	?	?	?	?	?	?	?	?	?	?	?	?	?	?	?	?	?	?	?	?
*Pagophila eburnea*	0	0	0	?	0	0	0	0	0	0	0	0	0	1	0	0	1	0	-	-	1	2	2	1	0	1	1	1	0	0	?	0	0	0	1	?	?	?	?	3	?	2	2	?	?	?	?	?	?	?	?	?	?	?	?	?	?	?	?	?	?	?	?	?	?	?	?	1	0	-	0	0	1	1	2
*Xema sabini*	0	0	0	?	0	0	0	0	0	0	0	0	0	1	0	1	1	0	0	-	1	1	1	0	0	1	1	0	0	0	?	0	A	0	1	?	?	?	?	4	2	2	1	?	?	?	?	?	?	?	?	?	?	?	?	?	?	?	?	?	?	?	?	?	?	?	?	1	0	-	1	0	1	1	0
*Rissa tridactyla*	0	0	0	?	0	0	0	0	0	0	0	0	0	1	0	1	1	0	0	0	1	2	2	0	0	1	1	1	0	0	?	3	0	0	1	?	0	1	0	4	?	2	2	?	?	?	?	?	?	?	?	?	?	?	?	?	?	?	?	?	0	0	0	1	0	1	1	1	0	-	0	0	1	1	0
*Cusorius temmneckii*	0	0	0	?	0	0	0	0	0	0	0	0	0	0	0	1	1	0	1	-	?	2	3	0	0	0	0	0	1	0	?	0	1	1	2	?	?	?	?	2	?	0	0	?	?	?	?	?	?	?	?	?	?	?	?	?	?	?	?	?	?	?	?	?	?	?	?	?	?	?	?	?	?	?	?
*Glareola maldivarum*	0	0	0	1	0	0	0	0	0	0	0	0	0	0	0	0	0	0	1	-	?	1	2	0	0	0	1	0	2	0	?	0	0	1	1	?	?	?	?	?	?	0	0	?	?	?	?	?	?	?	?	?	?	?	?	?	?	?	?	?	?	?	?	?	?	?	?	?	?	?	?	?	?	?	?
*Stiltia isabella*	0	0	0	?	0	0	0	0	0	0	0	0	0	0	0	1	1	1	1	0	?	3	1	1	0	0	1	0	2	0	?	0	0	1	1	?	?	?	?	4	2	0	0	?	?	?	?	?	?	?	?	?	?	?	?	?	?	?	?	?	?	?	?	?	?	?	?	0	-	-	-	1	1	1	1
*Rhinoptilus chalcopterus*	0	0	0	?	0	0	0	0	0	0	0	0	0	0	0	0	1	0	-	-	?	0	0	0	0	0	0	0	0	0	?	0	1	1	1	?	?	?	?	2	?	0	0	?	?	?	?	?	?	?	?	?	?	?	?	?	?	?	?	?	?	?	?	?	?	?	?	0	-	-	-	1	1	1	0
*Bartramia longicauda*	0	0	0	?	0	0	0	0	0	0	0	0	0	0	0	0	1	0	0	-	?	3	3	0	0	0	1	0	0	0	?	3	0	1	1	?	?	?	?	4	?	0	0	?	?	?	?	?	?	?	?	?	?	?	?	?	?	?	?	?	?	?	?	?	?	?	?	1	1	1	1	1	1	1	0
*Numenius minutus*	0	0	0	?	0	0	0	0	0	0	0	0	0	0	0	0	1	0	1	-	?	0	1	0	0	0	1	0	2	1	?	3	0	1	1	?	?	?	?	?	?	0	0	?	?	?	?	?	?	?	?	?	?	?	?	?	?	?	?	?	?	?	?	?	?	?	?	?	?	?	?	?	?	?	?
*Tryngites subruficollis*	0	0	0	?	0	0	0	0	0	0	0	0	0	0	0	0	1	0	?	?	?	0	3	0	0	0	1	0	2	0	?	0	1	1	1	?	?	?	?	1	?	0	0	?	?	?	?	?	?	?	?	?	?	?	?	?	?	?	?	?	?	?	?	?	?	?	?	1	0	-	0	1	1	1	0
*Hydrophasianus chirurgus*	0	0	0	?	0	0	0	0	0	0	0	0	0	1	0	0	0	0	1	-	?	3	1	0	0	0	1	0	1	0	?	3	1	1	1	?	?	?	?	4	?	0	0	?	?	?	?	?	?	?	?	?	?	?	?	?	?	?	?	?	?	?	?	?	?	?	?	?	?	?	?	?	?	?	?
*Charadrius vociferus*	0	0	0	?	0	0	1	0	0	0	0	0	0	1	0	1	1	0	0	?	?	0	0	0	0	0	0	1	0	0	0	0	1	1	1	1	1	0	0	4	2	0	0	?	?	?	?	?	?	?	?	?	?	?	?	?	?	?	?	?	?	?	?	?	?	?	?	1	1	0	0	1	1	1	1
*Charadrius wilsonia*	0	0	0	?	0	0	0	0	0	0	0	0	0	0	0	1	1	1	0	?	?	0	0	0	0	0	0	1	0	0	0	0	1	0	1	0	0	0	1	4	1	0	0	?	?	?	?	?	?	?	?	?	?	?	?	?	?	?	?	?	?	?	?	?	?	?	?	?	?	?	?	?	?	?	?
LACM 15410	?	?	?	?	?	?	?	?	?	?	?	?	?	?	?	?	?	?	?	?	?	?	?	?	?	?	?	?	?	?	?	?	?	?	?	?	?	?	?	?	?	?	?	?	?	?	?	?	?	?	?	?	?	?	?	?	?	?	?	?	?	?	?	?	?	?	?	?	?	?	?	?	?	?	?
LACM 128870	?	?	?	?	?	?	?	?	?	?	?	?	?	?	?	?	?	?	?	?	?	?	?	?	?	?	?	?	?	?	?	?	?	?	?	?	?	?	?	?	?	?	?	?	?	?	?	?	?	?	?	?	?	?	?	?	?	?	?	?	?	?	?	?	?	?	?	?	?	?	?	?	?	?	?
LACM 15425	?	?	?	?	?	?	?	?	?	?	?	?	?	?	?	?	?	?	?	?	?	?	?	?	?	?	?	?	?	?	?	?	?	?	?	?	?	?	?	?	?	?	?	?	?	?	?	?	?	?	?	?	?	?	?	?	?	?	?	?	?	?	?	?	?	?	?	?	?	?	?	?	?	?	?
LACM 23739	?	?	?	?	?	?	?	?	?	?	?	?	?	?	?	?	?	?	?	?	?	?	?	?	?	?	?	?	?	?	?	?	?	?	?	?	?	?	?	?	?	?	?	?	?	?	?	?	?	?	?	?	?	?	?	?	?	?	?	?	?	?	?	?	?	?	?	?	?	?	?	?	?	?	?
USNM 243765	?	?	?	?	?	?	?	?	?	?	?	?	?	?	?	?	?	?	?	?	?	?	?	?	?	?	?	?	?	?	?	?	?	?	?	?	?	?	?	?	?	?	?	?	?	?	?	?	?	?	?	?	?	?	?	?	?	?	?	?	?	?	?	?	?	?	?	?	?	?	?	?	?	?	?
LACM 107028	?	?	?	?	?	?	?	?	?	?	?	?	?	?	?	?	?	?	?	?	?	?	?	?	?	?	?	?	?	?	?	?	?	?	?	?	?	?	?	?	?	?	?	?	?	?	?	?	?	?	?	?	?	?	?	?	?	?	?	?	?	?	?	?	?	?	?	?	?	?	?	?	?	?	?
SDSNH 24262	?	?	?	?	?	?	?	?	?	?	?	?	?	?	?	?	?	?	?	?	?	?	?	?	?	?	?	?	?	?	?	?	?	?	?	?	?	?	?	?	?	?	?	?	?	?	?	?	?	?	?	?	?	?	?	?	?	?	?	?	?	?	?	?	?	?	?	?	?	?	?	?	?	?	?
SDSNH 59047	?	?	?	?	?	?	?	?	?	?	?	?	?	?	?	?	?	?	?	?	?	?	?	?	?	?	?	?	?	?	?	?	?	?	?	?	?	?	?	?	?	?	?	?	?	?	?	?	?	?	?	?	?	?	?	?	?	?	?	?	?	?	?	?	?	?	?	?	?	?	?	?	?	?	?
SDSNH 28152	?	?	?	?	?	?	?	?	?	?	?	?	?	?	?	?	?	?	?	?	?	?	?	?	?	?	?	?	?	?	?	?	?	?	?	?	?	?	?	?	?	?	?	?	?	?	?	?	?	?	?	?	?	?	?	?	?	?	?	?	?	?	?	?	?	?	?	?	?	?	?	?	?	?	?
SDSNH 42532	?	?	?	?	?	?	?	?	?	?	?	?	?	?	?	?	?	?	?	?	?	?	?	?	?	?	?	?	?	?	?	?	?	?	?	?	?	?	?	?	?	?	?	?	?	?	?	?	?	?	?	?	?	?	?	?	?	?	?	?	?	?	?	?	?	?	?	?	?	?	?	?	?	?	?
SDSNH 21295	?	?	?	?	?	?	?	?	?	?	?	?	?	?	?	?	?	?	?	?	?	?	?	?	?	?	?	?	?	?	?	?	?	?	?	?	?	?	?	?	?	?	?	?	?	?	?	?	?	?	?	?	?	?	?	?	?	?	?	?	?	?	?	?	?	?	?	?	?	?	?	?	?	?	?
LACM 103940	?	?	?	?	?	?	?	?	?	?	?	?	?	?	?	?	?	?	?	?	?	?	?	?	?	?	?	?	?	?	?	?	?	?	?	?	?	?	?	?	?	?	?	?	?	?	?	?	?	?	?	?	?	?	?	?	?	?	?	?	?	?	?	?	?	?	?	?	?	?	?	?	?	?	?
SDSNH 25236	?	?	?	?	?	?	?	?	?	?	?	?	?	?	?	?	?	?	?	?	?	?	?	?	?	?	?	?	?	?	?	?	?	?	?	?	?	?	?	?	?	?	?	?	?	?	?	?	?	?	?	?	?	?	?	?	?	?	?	?	?	?	?	?	?	?	?	?	?	?	?	?	?	?	?
*Mancalla californiensis*	?	?	?	?	?	?	?	?	?	?	?	?	?	?	?	?	?	?	?	?	?	?	?	?	?	?	?	?	?	?	?	?	?	?	?	?	?	?	?	?	?	?	?	?	?	?	?	?	?	?	?	?	?	?	?	?	?	?	?	?	?	?	?	?	?	?	?	?	?	?	?	?	?	?	?
*Mancalla cedrosensis*	?	?	?	?	?	?	?	?	?	?	?	?	?	?	?	?	?	?	?	?	?	?	?	?	?	?	?	?	?	?	?	?	?	?	?	?	?	?	?	?	?	?	?	?	?	?	?	?	?	?	?	?	?	?	?	?	?	?	?	?	?	?	?	?	?	?	?	?	?	?	?	?	?	?	?
*Mancalla lucasi*	?	?	?	?	?	?	?	?	?	?	?	?	?	?	?	?	?	?	?	?	?	?	?	?	?	?	?	?	?	?	?	?	?	?	?	?	?	?	?	?	?	?	?	?	?	?	?	?	?	?	?	?	?	?	?	?	?	?	?	?	?	?	?	?	?	?	?	?	?	?	?	?	?	?	?
*Mancalla vegrandis*	?	?	?	?	?	?	?	?	?	?	?	?	?	?	?	?	?	?	?	?	?	?	?	?	?	?	?	?	?	?	?	?	?	?	?	?	?	?	?	?	?	?	?	?	?	?	?	?	?	?	?	?	?	?	?	?	?	?	?	?	?	?	?	?	?	?	?	?	?	?	?	?	?	?	?
*Miomancalla howardi*	?	?	?	?	?	?	?	?	?	?	?	?	?	?	?	?	?	?	?	?	?	?	?	?	?	?	?	?	?	?	?	?	?	?	?	?	?	?	?	?	?	?	?	?	?	?	?	?	?	?	?	?	?	?	?	?	?	?	?	?	?	?	?	?	?	?	?	?	?	?	?	?	?	?	?
*Miomancalla wetmorei*	?	?	?	?	?	?	?	?	?	?	?	?	?	?	?	?	?	?	?	?	?	?	?	?	?	?	?	?	?	?	?	?	?	?	?	?	?	?	?	?	?	?	?	?	?	?	?	?	?	?	?	?	?	?	?	?	?	?	?	?	?	?	?	?	?	?	?	?	?	?	?	?	?	?	?
Mancallinae SST	?	?	?	?	?	?	?	?	?	?	?	?	?	?	?	?	?	?	?	?	?	?	?	?	?	?	?	?	?	?	?	?	?	?	?	?	?	?	?	?	?	?	?	?	?	?	?	?	?	?	?	?	?	?	?	?	?	?	?	?	?	?	?	?	?	?	?	?	?	?	?	?	?	?	?
							3	1	0								3	2	0								3	3	0								3	4	0																		
*Aethia cristatella*	?	?	?	?	?	?	?	?	?	?	?	?	?	?	?	?	?	?	?	?	?	?	?	?	?	?	?	?	?	?	?	?	?	?	?	?	?	?	?	?	?	?	?	?															
*Aethia pygmaea*	?	?	?	?	?	?	?	?	?	?	?	?	?	?	?	?	?	?	?	?	?	?	?	?	?	?	?	?	?	?	?	?	?	?	?	?	?	?	?	?	?	?	?	?															
*Aethia psittacula*	0	1	0	1	0	0	-	0	2	2	1	1	0	-	1	0	-	1	1	0	-	0	-	1	0	0	-	1	1	1	0	1	1	0	-	2	1	1	1	0	1	0	1	0															
*Aethia pusilla*	0	1	0	1	1	1	0	0	3	3	1	1	0	-	1	0	-	1	0	1	0	1	2	1	1	1	0	1	1	1	0	1	1	0	-	2	1	1	1	0	1	0	1	1															
*Ptychoramphus aleuticus*	0	1	0	1	0	0	-	0	3	3	1	1	0	-	1	0	-	1	1	1	1	1	2	1	0	1	0	1	1	1	0	1	1	0	-	2	1	1	1	1	1	0	1	1															
*Cerorhinca monocerata*	0	1	0	1	1	0	-	0	3	3	1	1	0	-	1	0	-	1	1	1	1	1	2	1	0	1	0	0	1	1	0	1	1	0	-	2	1	1	1	1	1	0	1	0															
*Fratercula arctica*	0	1	0	1	1	0	-	0	3	3	1	1	0	-	1	0	-	1	1	1	1	1	2	1	0	1	0	0	1	1	0	1	1	0	-	2	1	1	1	1	1	0	1	0															
*Fratercula corniculata*	?	?	?	?	?	?	?	?	?	?	?	?	?	?	?	?	?	?	?	?	?	?	?	?	?	?	?	?	?	?	?	?	?	?	?	?	?	?	?	?	?	?	?	?															
*Fratercula cirrhata*	2	1	0	1	1	0	-	0	3	3	1	1	0	-	1	0	-	1	1	1	1	1	2	1	1	1	0	0	1	1	1	1	1	0	-	2	1	1	1	1	1	0	1	0															
*Alca torda*	0	1	0	1	1	0	-	0	3	3	1	1	0	-	1	0	-	1	1	1	1	1	2	1	0	1	0	1	1	1	0	1	1	0	-	2	1	1	1	0	1	0	1	0															
*Pinguinus impennis*	?	?	?	?	?	?	?	?	?	?	?	?	?	?	?	?	?	?	?	?	?	?	?	?	?	?	?	?	?	?	?	?	?	?	?	?	?	?	?	?	?	?	?	?															
*Alle alle*	0	1	0	1	1	0	-	0	3	3	1	1	0	-	1	0	-	1	0	1	0	1	2	1	1	1	0	1	1	1	1	1	1	0	-	2	1	1	1	1	1	0	1	1															
*Uria aalge*	0	1	1	1	1	0	-	0	3	3	1	1	0	-	1	0	-	1	1	1	1	0	-	1	0	1	0	0	1	1	0	1	1	0	-	2	1	1	1	1	1	0	1	1															
*Uria lomvia*	?	?	?	?	?	?	?	?	?	?	?	?	?	?	?	?	?	?	?	?	?	?	?	?	?	?	?	?	?	?	?	?	?	?	?	?	?	?	?	?	?	?	?	?															
*Cepphus carbo*	?	?	?	?	?	?	?	?	?	?	?	?	?	?	?	?	?	?	?	?	?	?	?	?	?	?	?	?	?	?	?	?	?	?	?	?	?	?	?	?	?	?	?	?															
*Cepphus columba*	2	1	0	1	1	0	-	0	3	3	1	1	0	-	1	0	-	1	1	1	1	1	2	1	0	1	0	0	1	1	1	1	1	0	-	2	1	1	1	1	1	0	1	0															
*Cepphus grylle*	?	?	?	?	?	?	?	?	?	?	?	?	?	?	?	?	?	?	?	?	?	?	?	?	?	?	?	?	?	?	?	?	?	?	?	?	?	?	?	?	?	?	?	?															
*Brachyramphus brevirostris*	?	?	?	?	?	?	?	?	?	?	?	?	?	?	?	?	?	?	?	?	?	?	?	?	?	?	?	?	?	?	?	?	?	?	?	?	?	?	?	?	?	?	?	?															
*Brachyramphus marmoratus*	0	1	3	1	1	0	-	0	3	3	1	1	0	-	1	0	-	1	1	0	-	0	-	1	1	1	0	1	1	1	0	1	1	0	-	0	1	1	1	0	1	0	1	1															
*Brachyramphus perdix*	?	?	?	?	?	?	?	?	?	?	?	?	?	?	?	?	?	?	?	?	?	?	?	?	?	?	?	?	?	?	?	?	?	?	?	?	?	?	?	?	?	?	?	?															
*Synthliboramphus antiquus*	0	1	2	1	1	0	-	0	3	3	1	1	0	-	1	0	-	1	0	1	0	1	1	1	1	1	0	1	1	1	0	1	1	0	-	2	1	1	1	0	1	0	1	1															
*Synthliboramphus wumizusume*	?	?	?	?	?	?	?	?	?	?	?	?	?	?	?	?	?	?	?	?	?	?	?	?	?	?	?	?	?	?	?	?	?	?	?	?	?	?	?	?	?	?	?	?															
*Synthliboramphus craveri*	?	?	?	?	?	?	?	?	?	?	?	?	?	?	?	?	?	?	?	?	?	?	?	?	?	?	?	?	?	?	?	?	?	?	?	?	?	?	?	?	?	?	?	?															
*Synthliboramphus hypoleucus*	?	?	?	?	?	?	?	?	?	?	?	?	?	?	?	?	?	?	?	?	?	?	?	?	?	?	?	?	?	?	?	?	?	?	?	?	?	?	?	?	?	?	?	?															
*Stercorarius longicaudus*	2	1	2	1	0	1	0	0	0	0	1	0	1	1	1	0	-	1	1	1	1	0	-	1	0	1	0	0	1	1	0	1	1	1	1	1	1	1	1	0	1	0	1	0															
*Stercorarius skua*	1	1	2	1	0	1	0	0	0	0	1	0	1	1	1	0	-	0	-	0	-	0	-	0	-	0	-	-	1	1	0	1	1	1	1	1	1	1	1	2	1	0	1	1															
*Anous tenuirostris*	?	?	?	?	?	?	?	?	?	?	?	?	?	?	?	?	?	?	?	?	?	?	?	?	?	?	?	?	?	?	?	?	?	?	?	?	?	?	?	?	?	?	?	?															
*Chlidonias leucoptera*	?	?	?	?	?	?	?	?	?	?	?	?	?	?	?	?	?	?	?	?	?	?	?	?	?	?	?	?	?	?	?	?	?	?	?	?	?	?	?	?	?	?	?	?															
*Sterna niloteca*	0	0	-	-	-	1	0	0	0	0	1	0	1	0	0	0	-	0	-	0	-	0	-	0	-	0	-	-	1	0	-	1	1	1	1	0	0	0	0	-	0	0	0	-															
*Larosterna inca*	0	1	2	1	0	1	0	0	0	0	1	0	1	0	0	0	-	1	1	1	0	0	-	1	0	1	0	0	1	0	-	1	1	1	1	1	1	1	1	0	1	0	0	-															
*Sterna maxima*	?	?	?	?	?	?	?	?	?	?	?	?	?	?	?	?	?	?	?	?	?	?	?	?	?	?	?	?	?	?	?	?	?	?	?	?	?	?	?	?	?	?	?	?															
*Phaetusa simplex*	0	0	-	-	-	1	0	0	0	0	1	0	1	1	0	0	-	0	-	0	-	0	-	0	-	0	-	-	1	0	-	0	-	1	1	-	1	1	0	-	1	0	0	-															
*Sternula superciliaris*	?	?	?	?	?	?	?	?	?	?	?	?	?	?	?	?	?	?	?	?	?	?	?	?	?	?	?	?	?	?	?	?	?	?	?	?	?	?	?	?	?	?	?	?															
*Sterna anaethetus*	?	?	?	?	?	?	?	?	?	?	?	?	?	?	?	?	?	?	?	?	?	?	?	?	?	?	?	?	?	?	?	?	?	?	?	?	?	?	?	?	?	?	?	?															
*Gygis alba*	0	0	-	-	-	1	0	0	0	0	1	0	1	0	0	0	-	0	-	0	-	0	-	0	-	0	-	-	1	0	-	0	-	1	1	-	1	1	0	-	1	0	0	-															
*Rynchops niger*	0	0	-	0	-	1	0	0	0	0	1	0	1	0	0	0	-	0	-	0	-	0	-	0	-	0	-	-	1	0	-	1	0	1	1	1	0	0	0	-	1	0	0	-															
*Creagrus furcatus*	1	0	-	-	-	1	0	0	0	0	1	0	1	2	0	1	3	0	-	0	-	0	-	0	-	0	-	-	1	0	-	1	0	1	0	0	0	0	0	-	1	0	0	-															
*Rhodostethia rosea*	2	0	-	-	-	1	1	0	0	0	0	0	1	0	0	1	1	0	-	0	-	0	-	0	-	0	-	-	1	0	-	1	0	1	0	2	0	1	1	1	1	0	0	-															
*Larus argentatus*	1	0	-	0	-	1	1	0	0	0	0	-	0	-	0	1	1	0	-	0	-	0	-	0	-	0	-	-	1	0	-	1	0	0	0	0	0	1	1	0	1	0	0	?															
*Larus marinus*	?	?	?	?	?	?	?	?	?	?	?	?	?	?	?	?	?	?	?	?	?	?	?	?	?	?	?	?	?	?	?	?	?	?	?	?	?	?	?	?	?	?	?	?															
*Pagophila eburnea*	1	0	-	-	-	1	1	0	0	0	1	0	0	-	0	1	1	0	-	0	-	0	-	0	-	0	-	-	1	0	-	1	0	1	0	0	0	0	0	-	1	0	0	-															
*Xema sabini*	0	0	-	-	-	1	0	1	0	0	1	0	1	0	0	0	-	0	-	0	-	0	-	0	-	0	-	-	1	0	-	1	1	1	0	2	0	1	1	0	0	0	0	-															
*Rissa tridactyla*	2	0	-	-	-	1	1	0	0	0	1	0	0	-	0	1	1	0	-	0	-	0	-	0	-	0	-	-	1	0	-	1	1	1	0	2	0	0	0	-	1	0	0	-															
*Cusorius temmneckii*	?	?	?	?	?	?	?	?	?	?	?	?	?	?	?	?	?	?	?	?	?	?	?	?	?	?	?	?	?	?	?	?	?	?	?	?	?	?	?	?	?	?	?	?															
*Glareola maldivarum*	?	?	?	?	?	?	?	?	?	?	?	?	?	?	?	?	?	?	?	?	?	?	?	?	?	?	?	?	?	?	?	?	?	?	?	?	?	?	?	?	?	?	?	?															
*Stiltia isabella*	0	1	0	1	1	1	1	0	1	0	1	0	0	-	0	1	0	1	2	1	2	1	2	1	1	1	1	0	0	1	0	1	1	1	0	2	1	1	1	1	1	1	0	-															
*Rhinoptilus chalcopterus*	2	1	0	1	1	1	0	0	0	0	1	1	1	1	0	0	-	1	2	1	2	1	2	1	1	1	1	0	0	1	0	1	1	1	2	2	1	1	1	1	1	0	0	-															
*Bartramia longicauda*	0	1	0	1	1	1	0	0	0	0	1	0	1	0	0	0	-	1	0	1	0	1	0	1	1	1	1	0	0	1	1	1	1	1	1	0	1	1	1	1	1	0	0	-															
*Numenius minutus*	?	?	?	?	?	?	?	?	?	?	?	?	?	?	?	?	?	?	?	?	?	?	?	?	?	?	?	?	?	?	?	?	?	?	?	?	?	?	?	?	?	?	?	?															
*Tryngites subruficollis*	1	1	3	1	1	1	0	1	0	0	1	0	1	1	0	1	2	1	2	1	2	1	2	1	1	1	1	1	2	1	0	1	1	1	1	3	1	1	1	1	1	1	0	-															
*Hydrophasianus chirurgus*	?	?	?	?	?	?	?	?	?	?	?	?	?	?	?	?	?	?	?	?	?	?	?	?	?	?	?	?	?	?	?	?	?	?	?	?	?	?	?	?	?	?	?	?															
*Charadrius vociferus*	0	1	0	1	1	1	0	1	0	0	1	0	1	0	0	1	2	1	2	1	2	1	1	1	1	1	1	0	0	1	0	1	1	1	1	2	1	1	1	1	1	1	0	-															
*Charadrius wilsonia*	?	?	?	?	?	?	?	?	?	?	?	?	?	?	?	?	?	?	?	?	?	?	?	?	?	?	?	?	?	?	?	?	?	?	?	?	?	?	?	?	?	?	?	?															
LACM 15410	?	?	?	?	?	?	?	?	?	?	?	?	?	?	?	?	?	?	?	?	?	?	?	?	?	?	?	?	?	?	?	?	?	?	?	?	?	?	?	?	?	?	?	?															
LACM 128870	?	?	?	?	?	?	?	?	?	?	?	?	?	?	?	?	?	?	?	?	?	?	?	?	?	?	?	?	?	?	?	?	?	?	?	?	?	?	?	?	?	?	?	?															
LACM 15425	?	?	?	?	?	?	?	?	?	?	?	?	?	?	?	?	?	?	?	?	?	?	?	?	?	?	?	?	?	?	?	?	?	?	?	?	?	?	?	?	?	?	?	?															
LACM 23739	?	?	?	?	?	?	?	?	?	?	?	?	?	?	?	?	?	?	?	?	?	?	?	?	?	?	?	?	?	?	?	?	?	?	?	?	?	?	?	?	?	?	?	?															
USNM 243765	?	?	?	?	?	?	?	?	?	?	?	?	?	?	?	?	?	?	?	?	?	?	?	?	?	?	?	?	?	?	?	?	?	?	?	?	?	?	?	?	?	?	?	?															
LACM 107028	?	?	?	?	?	?	?	?	?	?	?	?	?	?	?	?	?	?	?	?	?	?	?	?	?	?	?	?	?	?	?	?	?	?	?	?	?	?	?	?	?	?	?	?															
SDSNH 24262	?	?	?	?	?	?	?	?	?	?	?	?	?	?	?	?	?	?	?	?	?	?	?	?	?	?	?	?	?	?	?	?	?	?	?	?	?	?	?	?	?	?	?	?															
SDSNH 59047	?	?	?	?	?	?	?	?	?	?	?	?	?	?	?	?	?	?	?	?	?	?	?	?	?	?	?	?	?	?	?	?	?	?	?	?	?	?	?	?	?	?	?	?															
SDSNH 28152	?	?	?	?	?	?	?	?	?	?	?	?	?	?	?	?	?	?	?	?	?	?	?	?	?	?	?	?	?	?	?	?	?	?	?	?	?	?	?	?	?	?	?	?															
SDSNH 42532	?	?	?	?	?	?	?	?	?	?	?	?	?	?	?	?	?	?	?	?	?	?	?	?	?	?	?	?	?	?	?	?	?	?	?	?	?	?	?	?	?	?	?	?															
SDSNH 21295	?	?	?	?	?	?	?	?	?	?	?	?	?	?	?	?	?	?	?	?	?	?	?	?	?	?	?	?	?	?	?	?	?	?	?	?	?	?	?	?	?	?	?	?															
LACM 103940	?	?	?	?	?	?	?	?	?	?	?	?	?	?	?	?	?	?	?	?	?	?	?	?	?	?	?	?	?	?	?	?	?	?	?	?	?	?	?	?	?	?	?	?															
SDSNH 25236	?	?	?	?	?	?	?	?	?	?	?	?	?	?	?	?	?	?	?	?	?	?	?	?	?	?	?	?	?	?	?	?	?	?	?	?	?	?	?	?	?	?	?	?															
*Mancalla californiensis*	?	?	?	?	?	?	?	?	?	?	?	?	?	?	?	?	?	?	?	?	?	?	?	?	?	?	?	?	?	?	?	?	?	?	?	?	?	?	?	?	?	?	?	?															
*Mancalla cedrosensis*	?	?	?	?	?	?	?	?	?	?	?	?	?	?	?	?	?	?	?	?	?	?	?	?	?	?	?	?	?	?	?	?	?	?	?	?	?	?	?	?	?	?	?	?															
*Mancalla lucasi*	?	?	?	?	?	?	?	?	?	?	?	?	?	?	?	?	?	?	?	?	?	?	?	?	?	?	?	?	?	?	?	?	?	?	?	?	?	?	?	?	?	?	?	?															
*Mancalla vegrandis*	?	?	?	?	?	?	?	?	?	?	?	?	?	?	?	?	?	?	?	?	?	?	?	?	?	?	?	?	?	?	?	?	?	?	?	?	?	?	?	?	?	?	?	?															
*Miomancalla howardi*	?	?	?	?	?	?	?	?	?	?	?	?	?	?	?	?	?	?	?	?	?	?	?	?	?	?	?	?	?	?	?	?	?	?	?	?	?	?	?	?	?	?	?	?															
*Miomancalla wetmorei*	?	?	?	?	?	?	?	?	?	?	?	?	?	?	?	?	?	?	?	?	?	?	?	?	?	?	?	?	?	?	?	?	?	?	?	?	?	?	?	?	?	?	?	?															
Mancallinae_SST	?	?	?	?	?	?	?	?	?	?	?	?	?	?	?	?	?	?	?	?	?	?	?	?	?	?	?	?	?	?	?	?	?	?	?	?	?	?	?	?	?	?	?	?															

## Appendix 5. Genbank accession numbers and authorship of molecular sequences used in this analysis

Key to lowercase letters in parentheses following accession numbers which denote authorship of sequences: **a** Baker et al. 2007 **b** Pereira and Baker 2008 **c** Paton and Baker 2006 **d** Bridge et al. 2005 **e** Moum et al. 2002 **f** Whittingham et al. 2000 **g** Yamamoto et al. 2005 **h** Moum et al. 1994 **i** Hebert et al. 2004 **j** Kerr et al. 2007 **k** Friesen et al. 1996 **l** Liebers et al. 2004 **m** Cohen et al. 1997 **n** Fain and Houde 2007 **o** Paton et al. 2003 **p** Groth and Barrowclough 1999.

**Table:**

Taxa	ND2	ND5	ND6	CO1	cyt b	12S rDNA	16S rDNA	RAG-1
*Aethia cristatella*	EF373219 (a)	---	X73928 (h)	EF380315 (b)	U37087 (k)	EF373064 (b)	EF380278 (b)	EF373165 (a)
*Aethia psittacula*	EF373235 (a)	---	X73925 (h)	EF380327 (b)	U37296 (k)	EF373077 (a)	EF380290 (b)	EF373179 (a)
*Aethia pusilla*	EF380337 (b)	---	X73926 (h)	EF380316 (b)	U37104 (k)	EF380303 (b)	EF380279 (b)	EF380266 (b)
*Aethia pygmaea*	EF380338 (b)	---	X73927 (h)	EF380317 (b)	U37286 (k)	EF380304 (b)	EF380280 (b)	EF380267 (b)
*Alca torda*	EF373220 (a)	AJ242683 (e)	X73916 (h)	EF380318 (b)	U37288 (k)	EF373065 (a)	EF380281 (b)	AY228788 (o)
*Alle alle*	EF373221 (a)	AJ242684 (e)	X73915 (h)	EF380319 (b)	U37287 (k)	AJ242684 (e)	EF380282 (b)	EF373166 (a)
*Anous tenuirostris*	EF373223 (a)	---	---	---	EF373119 (a)	EF373066 (a)	---	EF373168 (a)
*Bartramia longicauda*	EF373226 (a)	---	---	AY666283 (i)	EF373122 (a)	EF373069 (a)	---	EF373171 (a)
*Brachyramphus brevirostris*	EF373227 (a)	---	X73922 (h)	EF380321 (b)	U37289 (k)	EF380306 (b)	EF380284 (b)	EF373172 (a)
*Brachyramphus marmoratus*	EF380340 (b)	---	X73923 (h)	EF380322 (b)	U37290 (k)	EF380306 (b)	EF380285 (b)	EF380269 (b)
*Brachyramphus perdix*	EF380341 (b)	---	---	EF380323 (b)	U37291 (k)	EF380307 (b)	EF380286 (b)	EF380270 (b)
*Cepphus carbo*	EF380342 (b)	---	---	EF380324 (b)	U37292 (k)	EF380308 (b)	EF380287 (b)	EF380271 (b)
*Cepphus columba*	EF373229 (a)	---	X73918 (h)	EF380325 (b)	U37293 (k)	X76349 (h)	DQ674610 (n)	EF373173 (a)
*Cepphus grylle*	---	AJ242688 (e)	X73917 (h)	DQ433470 (j)	U37294 (k)	AJ242688 (e)	---	---
*Cerorhinca monocerata*	EF373230 (a)	---	---	EF380326 (b)	U37295 (k)	EF373072 (a)	EF380289 (b)	EF373174 (a)
*Charadrius vociferous*	DQ385082 (c)	DQ385150 (c)	---	DQ385167 (c)	DQ385218 (c)	DQ385269 (c)	DQ385286 (c)	AF143736 (p)
*Charadrius wilsonia*	---	---	---	AY666175 (j)	---	---	---	---
*Childonias leucopterus*	EF373231 (a)	---	---	---	EF373124 (a)	EF373073 (a)	---	EF373175 (a)
*Creagrus furcatus*	EF373234 (a)	---	---	---	EF373127 (a)	EF373076 (a)	---	EF373178 (a)
*Cursorius temminckii*	DQ385090 (c)	DQ385158 (c)	---	DQ385175 (c)	DQ385226 (c)	DQ385277 (c)	DQ385294 (c)	AY228780 (o)
*Fratercula arctica*	DQ385092 (c)	DQ385160 (c)	X73929 (h)	DQ385177 (c)	U37297 (k)	DQ385279 (c)	DQ385296 (c)	AY228787 (o)
*Fratercula cirrhata*	EF380343 (b)	---	X73931 (h)	EF380329 (b)	U37298 (k)	EF380309 (b)	EF380291 (b)	EF380273 (b)
*Fratercula corniculata*	EF380344 (b)	---	X73930 (h)	EF380328 (b)	U37299 (k)	EF380310 (b)	EF380292 (b)	EF380272 (b)
*Gelochelidon niloteca*	AY631383 (d)	---	---	DQ434167 (j)	AY631311 (d)	AY631347 (d)	---	EF373184 (a)
*Glareola maldivarum*	EF373241 (a)	---	---	---	EF373133 (a)	EF373083 (a)	---	---
*Gygis alba*	EF373242 (a)	---	---	---	AY631290 (d)	EF373084 (a)	---	EF373185 (a)
*Hydrophasianus chirurgis*	EF373243 (a)	AF146627 (f)	---	---	EF373135 (a)	EF373085 (a)	---	EF373186 (a)
*Larosterna inca*	AY631364 (d)	---	---	---	AY631292 (d)	AY631328 (d)	---	EF373190 (a)
*Larus argentatus*	---	---	---	DQ433743 (j)	AJ508101 (l)	---	---	---
*Larus marinus*	EF373246 (a)	---	---	DQ433757 (j)	AJ508140 (l)	EF373088 (a)	---	AY228799 (o)
*Numenius minutus*	EF373253 (a)	---	---	---	EF373145 (a)	EF373095 (a)	---	EF373195 (a)
*Pagophila eburnea*	EF373255 (a)	---	---	DQ433862 (j)	EF373147 (a)	EF373097 (a)	---	EF373198 (a)
*Phaetusa simplex*	AY631365 (d)	---	---	---	AY631293 (d)	AY631329 (d)	---	EF373200 (a)
Taxa	ND2	ND5	ND6	CO1	cyt b	12S rDNA	16S rDNA	RAG-1
*Pinguinus impennis*	---	AJ242685 (e)	AJ242685 (e)	---	AJ242685 (e)	AJ242685 (e)	---	---
*Ptychoramphus aleuticus*	EF373261 (a)	---	X73924 (h)	EF380330 (b)	U37302 (k)	EF373103 (a)	EF380293 (b)	EF373204 (a)
*Rhinoptilus chalcopterus*	EF373263 (a)	---	---	---	EF373154 (a)	EF373105 (a)	---	EF373205 (a)
*Rhodostethia rosea*	EF373264 (a)	---	---	DQ434048 (j)	EF373155 (a)	EF373106 (a)	---	EF373206 (a)
*Rissa tridactyla*	DQ385093 (c)	DQ385161 (c)	---	DQ385178 (c)	DQ385229 (c)	DQ385280 (c)	DQ385297 (c)	AY228785 (o)
*Rynchops niger*	DQ385094 (c)	DQ385162 (c)	---	DQ385179 (c)	DQ385230 (c)	DQ385281 (c)	DQ385298 (c)	AY228784 (o)
*Stercorarius longicaudus*	EF373267 (a)	---	---	DQ434147 (j)	U76820 (m)	EF373109 (a)	---	EF373208 (a)
*Stercorarius skua*	DQ385091 (c)	DQ385159 (c)	---	DQ385176 (c)	DQ385227 (c)	DQ385278 (c)	DQ385295 (c)	AY228783 (o)
*Sterna anaethetus*	AY631368 (d)	---	---	DQ433203 (j)	AY631296 (d)	AY631332 (d)	---	---
*Sterna maxima*	AY631381 (d)	---	---	DQ434165 (j)	AY631309 (d)	DQ674571 (n)	DQ674609 (n)	---
*Sternula superciliaris*	AY631388 (d)	---	---	---	AY631316 (d)	AY631352 (d)	---	EF373210 (a)
*Stiltia isabella*	EF373268 (a)	---	---	---	EF373159 (a)	EF373110 (a)	---	EF373211 (a)
*Synthliboramphus antiquus*	EF373269 (a)	AP009042 (g)	X73920 (h)	EF380331 (b)	U37303 (k)	EF373111 (a)	EF380294 (b)	EF373212 (a)
*Synthliboramphus craveri*	EF380345 (b)	---	---	EF380332 (b)	U37304 (k)	EF380311 (b)	EF380295 (b)	EF380274 (b)
*Synthliboramphus hypoleucus*	---	---	X73921 (h)	DQ434184 (j)	U37305 (k)	---	---	---
*Synthliboramphus wumizusume*	EF380346 (b)	---	X73919 (h)	EF380333 (b)	U37306 (k)	EF380312 (b)	EF380296 (b)	EF380275 (b)
*Tryngites subruficollis*	EF373272 (a)	---	---	AY666178 (i)	EF373162 (a)	EF373114 (a)	---	EF373215 (a)
*Uria aalge*	EF380348 (b)	AJ242686 (e)	X73913 (h)	EF380334 (b)	U37307 (k)	DQ485794 (n)	DQ485832 (n)	EF380276 (b)
*Uria lomvia*	EF373273 (a)	AJ242687 (e)	X73914 (h)	EF380336 (b)	U37308 (k)	AJ242687 (e)	EF380299 (b)	EF373216 (a)
*Xema sabini*	EF373275 (a)	---	---	---	EF373164 (a)	EF373116 (a)	---	EF373217 (a)

## Appendix 6. Geologic setting

Mancallinae material described herein comes from four Miocene and Pliocene aged marine deposits (Domning and Deméré 1984; Ingle 1979; Wagner et al. 2001). Congruent with the habitat of extant alcids (del Hoyo et al 1996), three of these deposits (San Mateo Formation, Niguel Formation, San Diego Formation) are interpreted as the result of shallow to moderate depth marine facies (Vedder 1960; Kern and Wicander 1974; Vedder 1972; Ingle 1979; Wagner et al. 2001) associated with cold-water upwelling ocean systems. The upper siltstone facies of the Capistrano Formation, from which Mancallinae fossils have been recovered, contains transported remains of neritic mollusks and microfossils that are mixed with the remains of bathyal species (Kern and Wicander 1974), suggesting a shallow water origin for Mancallinae fossils from the Capistrano Formation. As with other vertebrate fossil assemblages fromnutrient-rich cold-water systems (e.g., Pliocene Yorktown Formation assemblage; Ray 1987; Ray and Bohaska 2001), a diverse assemblage of vertebrates including marine mammals and seabirds are documented from marine deposits such as the Pliocene San Diego formation in southern California (Barnes et al. 1981).

**San Mateo Formation:** The San Mateo Formation is composed of sandstones, siltstones, and conglomerates that interfinger with the latest Miocene and earliest Pliocene aged member of the Capistrano Formation (Tan and Kennedy 1996), and is interpreted as the result of shallow marine deposition (Vedder 1972). The San Mateo Formation is exposed in natural and quarry exposures near Lawrence Canyon in San Diego County, California, and has yielded two distinct vertebrate assemblages including sharks, fish, birds, and marine and terrestrial mammals (Barnes et al. 1981; Domning and Deméré 1984; Howard 1982).

The vertebrate assemblages of the San Mateo Fm. were discussed by Barnes et al. (1981), who designated the lower assemblage the San Luis Rey River Local Fauna (SLRRLF), and the upper assemblage the Lawrence Canyon Local Fauna (LCLF). Based on marine vertebrates and terrestrial mammals, the age of the younger LCLF has been proposed to be latest Miocene or earliest Pliocene (~5.0 Ma), and correlative with the Late Hemphillian North American Land Mammal Age (NALMA; Domning and Deméré 1984). Mancallinae fossils, including the humerus (SDSNH 24584) referred to *Mancalla howardi*, have been recovered from the older SLRRLF. Age estimates for the SLRRLF based upon terrestrial mammal and marine bird fossils range from approximately 6.7-10.0 Ma (i.e., Late Miocene or Turtonian equivalent; Barnes et al. 1981, Domning and Deméré 1984).

**Capistrano Formation:** The Capistrano Fm. is composed of sandstones and siltstones that have been correlated with upper portions of the San Mateo Fm. in northern San Diego County (Elliot 1975; Domning and Deméré 1984), and interpreted as the result of marine deep-sea fan deposition on the basis of microfaunal analysis and abundant turbidites (Ingle 1979; Vedder 1972). The Capistrano Fm. spans the Late Miocene-Early Pliocene boundary (Deméré and Berta 2005), and has accordingly been subdivided into upper and lower units. The age of the lower unit is estimated at 5.6-6.4 Ma (i.e., Late Miocene or Late Messinian; Barron 1986). Although no refined estimates are known for the uppermost siltstone unit from which the holotype of *Miomancalla howardi* was recovered, microfaunal analysis of the Capistrano Fm. has identified diatoms with ages as young as 4.9Ma (Early Pliocene or Early Zanclian; Deméré and Berta 2005).

**Niguel Formation:** The Niguel Formation is composed of a mixed sequence of marine and non-marine siltstones, sandstones, and conglomerates (Ingle 1979). Microfaunal and molluscan analysis indicates deposition at relatively shallow depth (i.e., <200m; Vedder 1960) during the Late Pliocene and Early Pleistocene (Late Piacenzian-Early Calabrian; Vedder 1960; Ingle 1979), with sea-surface temperatures similar to those offshore southern California today (i.e., nutrient rich cold water system; Ingle 1979).

**San Diego Formation:** The San Diego Formation predominantly consists of Pliocene and Pleistocene marine sandstones with minor amounts of conglomerates and claystones, which are, interpreted as shore-face and shallow depth shelf facies deposits (Deméré 1983; Wagner et al. 2001). Based upon microfaunal analysis and correlation with mammalian and molluscan assemblages of known age, the age of San Diego Fm. sediments are estimated to range from 3.6-1.5Ma (i.e., Middle Pliocene to Early Pleistocene; Piacenzian-Early Calabrian; Wagner et al. 2001). The San Diego Fm. was divided into 7 stratigraphic sub-units by Wagner et al. (2001). *Mancalla* fossils occur throughout the San Diego Fm. (T. Deméré pers. comm.), Paleomagnetic analysis indicates that sub-unit two can be correlated with the Gilbert Chron C2Ar and Gauss Chron C2An.3n boundary, which has been assigned an age of 3.6Ma (Wagner et al. 2001). The distribution of *Mancalla* species with respect to sub-units within the San Diego Fm. has not been evaluated.
